# Association between psychological depression and physical health in Chinese empty-nesters during the COVID-19 pandemic

**DOI:** 10.3389/fpsyt.2025.1473783

**Published:** 2025-02-18

**Authors:** Shiyi Wang, Xiaojing Fan, Fang Li, Zhongliang Zhou

**Affiliations:** ^1^ School of Public Policy and Administration, Xi’an Jiaotong University, Xi’an, Shaanxi, China; ^2^ Psychiatric Department, Xi’an Union Hospital, Xi’an, Shaanxi, China

**Keywords:** empty-nesters, psychological depression, physical health, COVID-19, healthy aging

## Abstract

**Objectives:**

With the aging of the population becoming increasingly serious, the phenomenon of empty-nesters is also becoming more and more serious. The psychological problems of the empty-nester elderly are becoming more and more prominent, which may affect their physical health. This study aimed to quantify the association between psychological depression and physical health in the background of the COVID-19 pandemic.

**Methods:**

A total of 7,835 empty-nesters over 60 years old were selected from the China Health and Retirement Longitudinal Study in 2020. Depressive symptoms were applied to measure psychological health. The outcome variables of physical health were measured by self-rated health (SRH), chronic disease, and limited basic activities of daily living (BADLs). Binary logistic regression models with odds ratios and 95% confidence intervals (CI) were applied to explore the association between psychological and physical health.

**Results:**

Empty-nesters with depressive symptoms were 72% less likely to rate their health as good than empty-nesters without depressive symptoms (95%CI: 0.242–0.328). The rates of chronic disease for empty-nesters with depressive symptoms were 1.45 (95%CI: 1.300–1.622) times higher than those without depressive symptoms, and those with depressive symptoms were more likely to have limited BADLs than elderly without depressive symptoms (OR = 3.125, 95%CI: 2.757–3.543).

**Conclusion:**

We found that among empty-nesters in China, depressive symptoms were negatively associated with good self-rated health and positively associated with chronic diseases and limitations in BADLs.

## 1 Introduction

Globally, all regions and areas are experiencing population aging. Population aging is an inevitable consequence of the demographic transition, which occurs due to a shift from higher to lower levels of fertility and mortality, which ultimately leads to a significant increase in the proportion of older persons in the total population. According to the World Population Ageing 2023 report, developed countries are expected to enter an advanced stage of population aging, with the proportion of older persons rising from 20% in 2023 to 28% in 2050 (1). According to the Statistical Yearbook of the China Statistics Bureau, at the end of 2022, 19.86% of the total population was aged 60 and older (2).

Empty-nesters are older adults who either live alone or live only with their spouse, without their children in the household (2). Data from the National Bureau of Statistics shows that the proportion of empty-nesters among China’s elderly population will be more than half by the end of 2023. The psychological and physical health status of empty-nesters has been a widespread academic concern. They are vulnerable to various diseases and health problems (5, 6), which will influence economic development and social stability. Psychological health is closely linked to physical health. Studies by international scholars have shown that the migration of adult children is associated with higher levels of depression and poorer self-rated health (SRH) among empty-nesters (3). Pack et al. concluded that empty-nesters are at a higher risk of poor psychological health compared to other non-empty-nesters of the same age and identified the key factors influencing their mental health (4). Previous studies have shown important links between psychological and physical health. Psychological health significantly influences physical health outcomes (7). Due to the long-term absence of companionship and emotional support from their children, empty-nesters are more prone to physical and psychological problems such as organic dysfunction, lower quality of life, higher risk of death and depression, anxiety, and loneliness (8, 9). A meta-analysis of English and Chinese literature found that nearly half of the Chinese empty-nesters are depressed (10).

The emergence of the COVID-19 pandemic in 2020 spread across most of the population of the world with significant health consequences and mortality among those who become infected. In addition to the direct impacts of COVID-19, the pandemic has resulted in environmental changes such as social restrictions, blockades, closure of schools and businesses, loss of livelihoods, and reduced economic activity, which have the potential to cause a significant impact on people’s psychological health (11). Another review (12) shows that older people are more likely to suffer psychological health issues associated with COVID-19 and that the older population reported stress, depression, anxiety, and loneliness during the pandemic, which is similar to the Canadian study (13), especially for those empty-nesters who need more support (14).

However, previous research on the health of the elderly has mainly focused on attention to physical health or psychological health. With limited studies systematically addressing the interplay between the two, particularly the impact of psychological health on physical health outcomes. A study (15) discovered a higher prevalence of depression among empty-nesters but overlooked the effects of depression on physical health outcomes. Similarly, some scholars have examined social exclusion and health outcomes among empty-nesters and non-empty-nester individuals (16) but lack an in-depth analysis of how psychological health influences physical health. Furthermore, during the COVID-19 pandemic, the interactions between psychological and physical health have become increasingly relevant (17), yet systematic investigations remain scarce. These gaps underscore the need for this study, which aims to study the associations between psychological health and physical health outcomes in elderly populations. Consequently, our team attempted to bridge this gap by analyzing the results of a large national survey to answer the two questions listed below:

- What is the current status of empty-nesters in the elderly population in China in 2020?
- What physical health indicators of empty-nesters are affected by psychological health and how strong is the impact?

## 2 Methods

### 2.1 Data source

The China Health and Retirement Longitudinal Study (CHARLS) was initiated and conducted by the National Development Research Institute of Peking University to promote the study of population aging and health issues, with a target population of 45 years old and above. The baseline national wave of CHARLSwas launched in 2011 and was conducted in 150 counties, 450 villages, and committees in 28 provinces. The project utilizes multi-stage sampling with PPS sampling methodology at both the district and village sampling stages. It also uses the first electronic mapping software (CHARLS-GIS) technology to produce village sampling frames by the map method. The study data and questionnaire are publicly available (https://charls.charlsdata.com/pages/Data/2020-charls-wave5/zh-cn.html).

### 2.2 Empty-nesters

Empty-nesters are elderly people living alone or elderly couples without children to care for them (2). In this study, empty-nesters were screened based on the following four questions in the CHARLS (**Supplementary Table S1**):

“How many months have you lived with your children in the past year?” (CA014)

“How often do you see your children when you do not live with them?” (CA015)

“For the first half-year, how many days did you live by yourself?” (BA018)

“For the first half-year, how many days did you live with just your spouse/partner (no one else but your spouse)?” (BA019)

The respondents were classified as elderly people who met one of two criteria as empty-nesters: (1) do not live with their children (over 1 month) and (2) elderly people living alone (over 1 month).

### 2.3 Sample size

This study utilized data obtained from the most recent 2020 survey, which included a total of 19,395 respondents, and after combining and matching the data, there were 19,349 respondents. The research topic focused on empty-nesters aged 60 years and above, and the sample size was selected through the following process: ①8,718 respondents under the age of 60 were excluded and ②2,796 non-empty-nesters were excluded according to the definition of empty-nesters. Lastly, 7,835 empty-nesters aged 60 and above were obtained as the subjects of this study to quantitatively explore the extent of the association between psychological health and physical health.

## 3 Indicators

### 3.1 Outcome variable—physical health

This study used three indicators to conceptualize the physical health of empty-nesters: SRH, diagnosed chronic diseases or not, and limited basic activities of daily living (BADLs) or not. SRH is one of the most frequently employed health measures in social science research based on a simple question such as: “In general, how would you rate your health?” (18). In CHARLS face-to-face survey, the respondents were asked “What do you think of your health? Is it very good, good, average, not good, or very bad?”, with rating values of 1, 2, 3, 4, and 5, respectively. The higher the score, the worse they feel about themselves. According to previous studies (19), the five-category variable was divided into two-category variables in our analysis: good SRH or not. The answers including “average”, “poor”, and “very poor” were grouped as not good SRH, and the answers including “very good” and “good” were grouped as good SRH. Diagnosed chronic diseases or not in CHARLS was based on the respondents’ reports on the following item: “Have you been diagnosed with [conditions listed below … ] by a doctor?” The CHARLS questionnaire looked at 15 chronic diseases (hypertensive disease, dyslipidemia, diabetes mellitus, cancer and other malignant tumors, chronic lung disease, liver disease, heart disease, stroke, kidney disease, gastric or digestive disorders, emotional and psychiatric problems, memory-related disorders, Parkinson’s disease, arthritis or rheumatism, asthma). This outcome variable was a binary variable, whereby 0 denoted “having no chronic disease” and 1 denoted “having one of these chronic diseases.” BADLs reflect six items measuring dressing, bathing, eating, getting out of bed, toileting, and continence. Each item consisted of four response options (1—no difficulty, 2—difficult but achievable, 3—some difficulties and need help, and 4—unable to complete). We recoded the outcome as a binary variable, and the respondents who had any difficulties in these activities were categorized as suffering from limited BADLs (20). Limited BADLs were defined as those respondents scoring 2, 3, or 4 on any item of the BADLs scale.

### 3.2 Independent variables

The core independent variable was psychological health. Depressive symptoms were selected as the psychological health indicator. Depressive symptoms were measured using the 10-item version of the Center for Epidemiologic Studies Depression scale (CESD-10), which is a comprehensive and credible assessment tool (21, 22). The CESD-10 contains 10 questions, including feeling troubled, feeling difficulty concentrating, feeling depressed, feeling overwhelmed by the effort, feeling hopeful, feeling afraid, sleeping disordered, feeling happy, feeling lonely, and feeling unable to continue living—each with four options about the past week: (1) rarely or none of the time, <1 day; (2) some of the time, 1 to 2 days; (3) much or a moderate amount of the time, 3 to 4 days; and (4) most or all of the time, 5–7 days, where positive emotions are scored inversely. The total score ranges from 0 to 30, with higher scores indicating higher levels of depressive symptoms. It has been shown that Cronbach’s coefficient of.87 for CES-D 10 has high validity (23). In the study, we categorized the participants whose score was <10 into the no depressive symptoms group and those with score ≥10 into the depressive symptoms group (24).

Based on prior literature (25, 26), we included demographic factors such as age, gender, marital status, and resident type. Children’s financial support (no support, low support, or high support) was evaluated by asking the respondents the following question: “In the past year, how much financial support did you receive from your children when they did not live with you?” The results were categorized as follows: children’s support less than 25% took the value 0 and was called “no support”, those between 25% and 75% were “low support”, and those above 75% was “high support”. “Education levels” consist of the following four layers: illiteracy, primary school and below, middle school, and high school and above. In this study, per capita household consumption expenditure was used to measure the economic level of the study population because it had been confirmed by studies that it was more accurate than household income (27). We divided per capita household consumption into quintiles. Health-related information included sleeping time at night, smoking status, and alcohol consumption.

### 3.3 Statistical analyses

For the treatment of control variables, the educational attainment variable, which had a high number of missing values, was matched to the 2011, 2013, 2015, and 2018 data. For the remaining small number of missing values in the independent variables, these were treated as system-missing and excluded from the analysis. Chi-square test was used to compare the depressive symptoms of empty-nesters with and without chronic disease, self-rated good health or not, and limited BADLs or not. Binary logistic regression model was used to explore the specific association of psychological health on the physical health of the empty-nesters. Considering that age, gender, marital status, residence, children’s support, education, per capita household expenditure, sleeping time, smoking status, and alcohol consumption may affect the physical health of the elderly, we incorporated these factors as covariates. The results were expressed as odds ratios (OR) and their 95% confidence intervals (95%CI). In all of the results, *p* <.05 was used as the criterion for statistically significant differences. All of the data analyses were performed using StataMP17.0.

## 4 Results

### 4.1 Distribution of basic information

The characteristics of the respondents are displayed in **Table 1**. The 7,835 respondents included 3,671 (46.85%) men and 4,165 (53.15%) women. Among them, 2,551 (32.60%) lived in urban areas and 5,274 (67.40%) lived in rural areas. A total of 28.40% of the empty-nesters were aged 60–65 years, 28.65% were aged 65–70 years, 19.18% were aged 70–75 years, and 23.77% were over 75 years old. A total of 42.02% of the empty-nesters had chronic diseases, and 78.78% of the empty-nesters stated that their SRH was not good. A total of 44.79% of the empty-nesters had depressive symptoms. The basic characteristics of the selected subjects, categorized by subgroups of different depressive symptoms, are presented in **Supplementary Table S2**. The proportion of empty-nesters who were female (53.99%), unmarried (54.40%), illiterate (57.77%), and lacked proper sleep (57.91%) had a higher proportion of depressive symptoms.

**Table 1:** Basic characteristics of empty-nesters (*n* = 7,835).

Variables	Groups	*N* (%)	Variables	Groups	*N* (%)
**Socio-demographic**			**Health status**		
Gender	Male	3,671 (46.85)	Sleeping time	6–8	2,750 (35.10)
	Female	4,165 (53.15)		<6	3,060 (39.06)
Age (years)	60–65	2,225 (28.40)		≥8	2,025 (25.85)
	65–70	2,245 (28.65)	Smoking status	Current	1,857 (23.72)
	70–75	1,503 (19.18)		Former	1,249 (15.95)
	≥75	1,862 (23.77)		Never	4,724 (60.33)
Marital status	Married	5,309 (67.76)	Alcohol consumption	No	5,336 (68.15)
	Unmarried	2,526 (32.24)		Yes	2,494 (31.85)
Residential area	Urban	2,551 (32.60)	**Psychological health**		
	Rural	5,274 (67.40)	Depressive symptoms	No	3,533 (55.21)
Children’s support	No support	2,270 (28.97)		Yes	2,866 (44.79)
	Low support	3,418 (43.62)	**Physical health**		
	High support	2,147 (27.40)	Self-rated health	Very poor	511 (7.37)
Educational level	Illiteracy	2,419 (32.86)		Poor	1,524 (21.98)
	≤Primary school	3,219 (43.73)		Average	3,427 (49.43)
	Middle school	1,070 (14.54)		Good	776 (11.19)
	≥High school	653 (8.87)		Very good	695 (10.02)
Per capita household expenditure	Low	1,565 (20.00)	Chronic diseases	No	4,543 (57.98)
	Lower	1,565 (20.00)		Yes	3,292 (42.02)
	Middle	1,566 (20.01)	BADLs	Normal	5,398 (68.94)
	Higher	1,564 (19.99)		Limited	2,432 (31.06)
	Highest	1,565 (20.00)			

Bold values indicate variable category headings.

### 4.2 Univariate analyses

**Figure 1:**
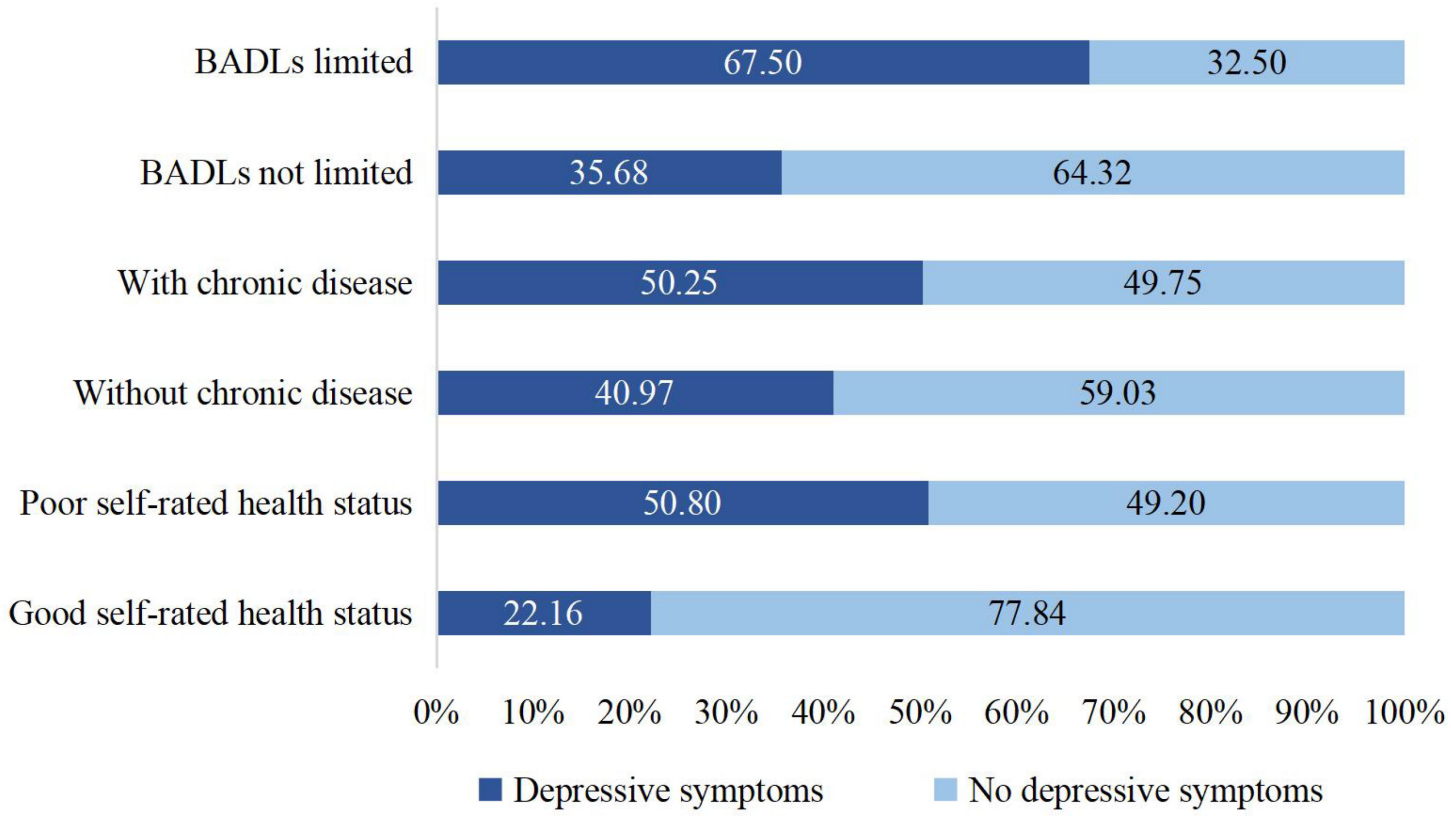
Distribution of depressive symptoms by different physical symptoms.

There were significant differences in depressive symptoms among empty-nesters based on their SRH. Empty-nesters with good SRH reported less severe depressive symptoms compared to those with poorer SRH (*χ*² = 354.26, *p* <.001). The severity of depressive symptoms among empty-nesters with chronic diseases was greater than that among those without chronic diseases (*χ*² = 53.91, *p* <.001). Additionally, there was a significant association between BADLs and depressive symptoms, and those who had limited BADLs were more likely to have depressive symptoms (*χ*² = 535.23, *p* <.001) (**Figure 1**).

### 4.3 Multivariate analyses

**Figure 2:**
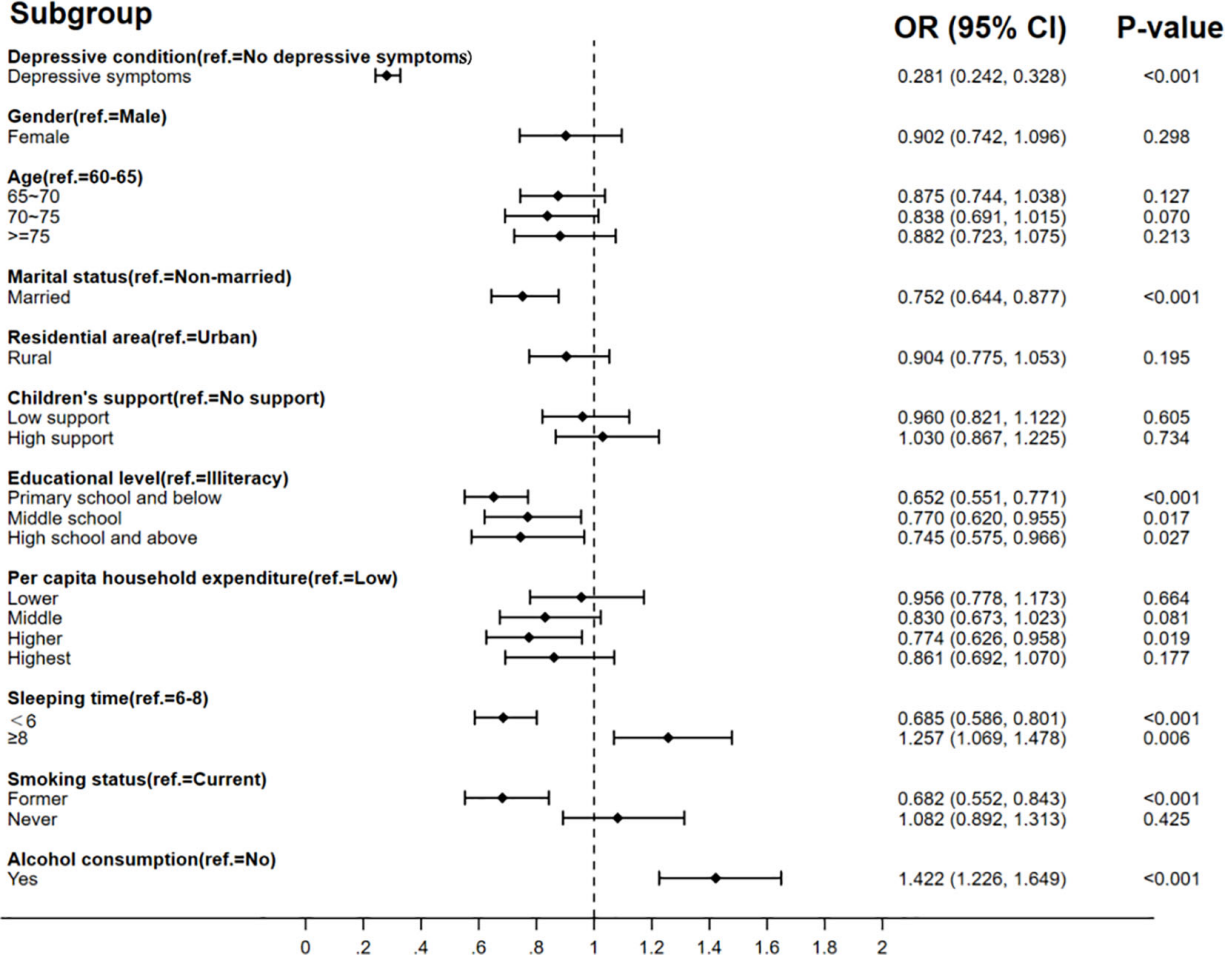
Association between depressive symptoms and SRH.

**Figure 3:**
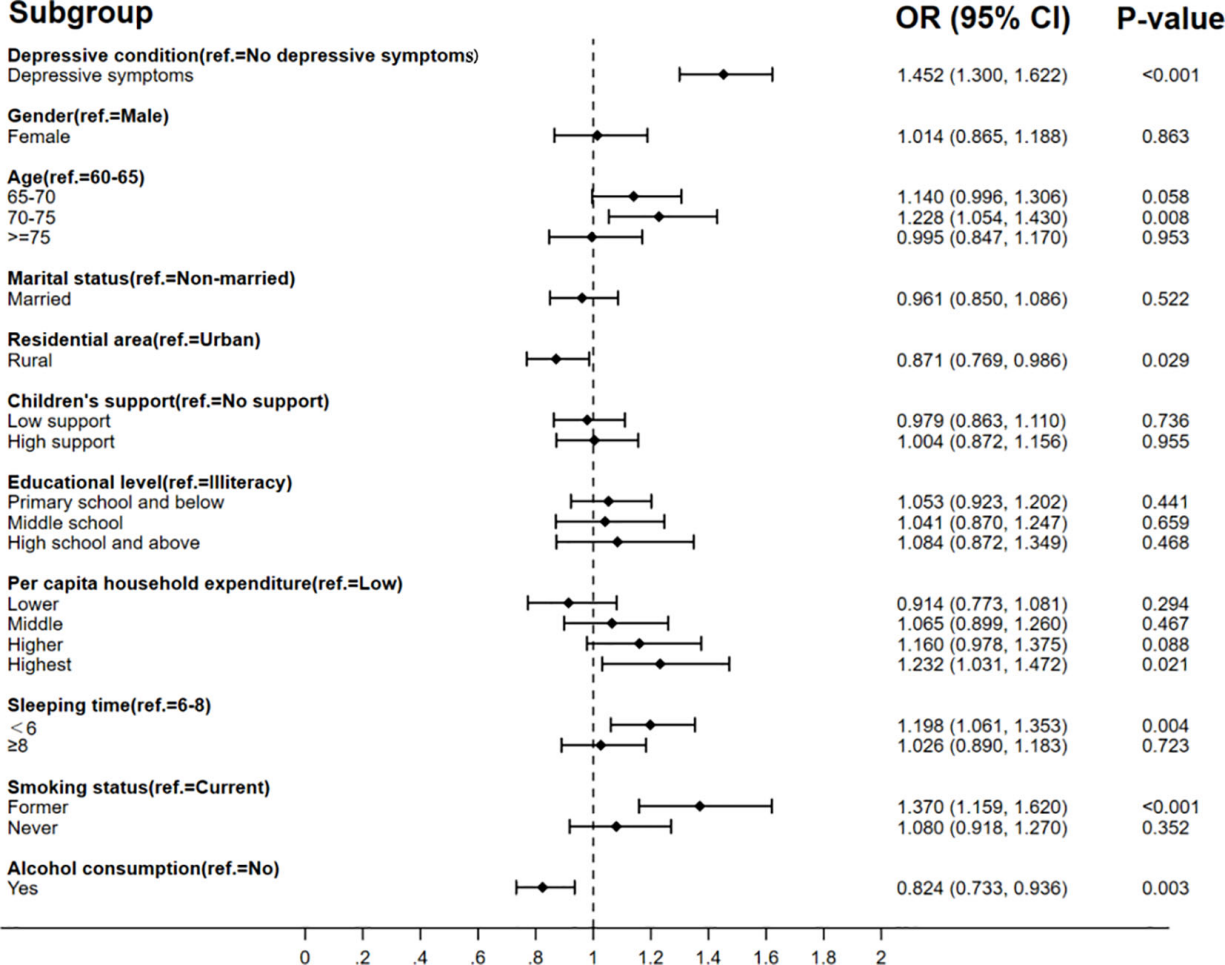
Association between depressive symptoms and chronic disease.

**Figure 4:**
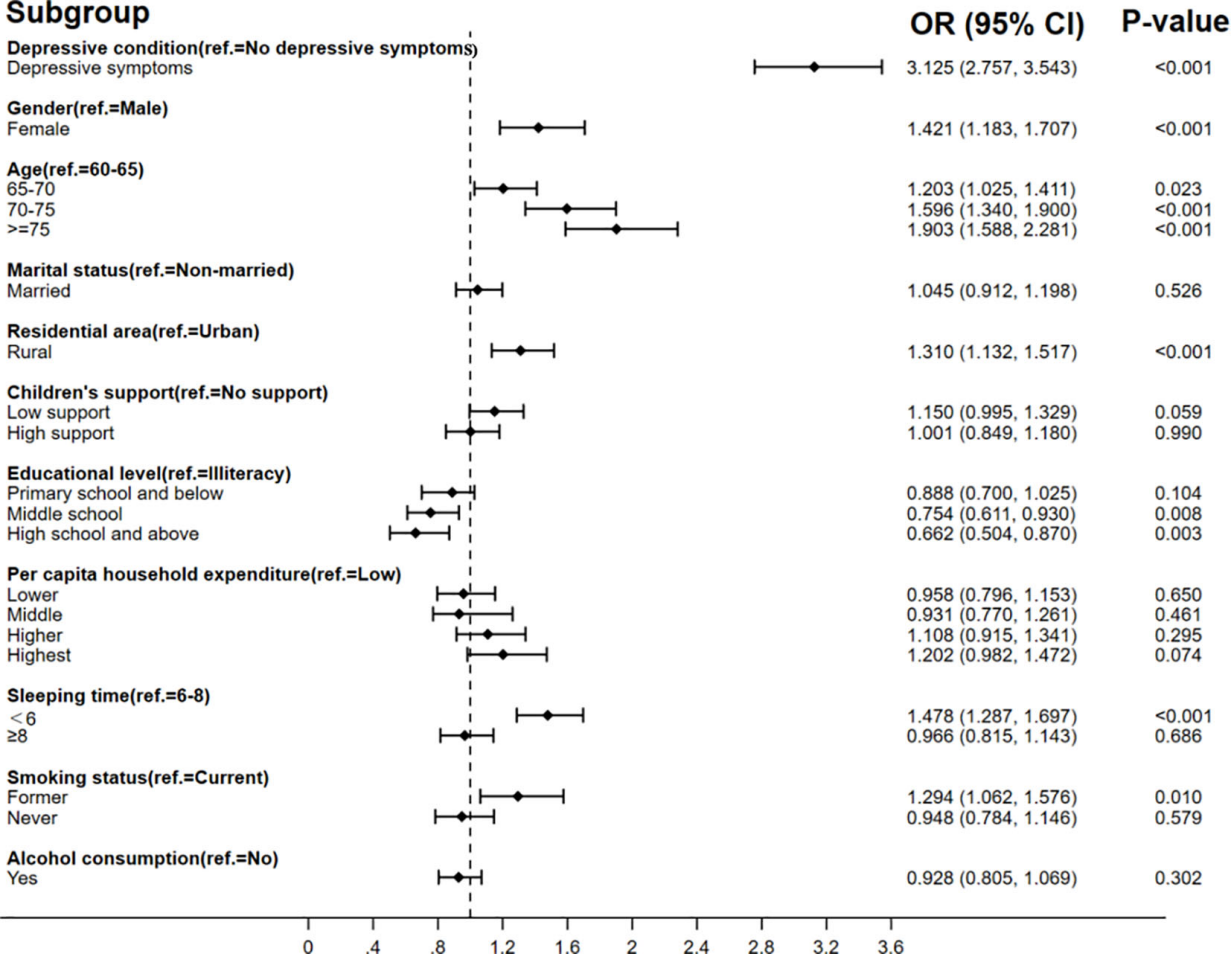
Association between depressive symptoms and limited BADLs.

The binary logistic regression model in **Figure 2** shows that there was a positive association between depressive symptoms and poor SRH after controlling for the variables of gender, age, marital status, residence, children’s support, education level, per capita household expenditure, sleeping time, smoking status, and alcohol consumption. Older adults with depressive symptoms were 72% less likely to consider themselves in good health compared to those without depressive symptoms (OR = 0.281, 95%CI: 0.242–0.328). The binary logistic regression model in **Figure 3** also presents different depressive symptoms as significantly connected with chronic disease or not. The rate of chronic disease was 1.452 (95%CI: 1.300–1.622) times higher in empty-nesters with depressive symptoms compared to those without depressive symptoms. The results indicated an association between depressive symptoms and limited BADLs in elderly individuals in **Figure 4** (OR = 3.125, 95%CI: 2.757–3.543).

The results also found that different factors influence SRH, chronic disease symptoms, and limited BADLs. It found that SRH is also influenced by marital status, education level, hours of sleep, and alcohol consumption. Factors influencing the prevalence of chronic diseases included residence, hours of sleep, smoking status, and alcohol consumption. Gender, age, residence, education level, hours of sleep, and smoking status could affect limited BADLs.

We use the simple random deletion method and replacement probit model in this paper to test the robustness. The results of the robustness test are shown in **Supplementary Tables S3**, **S4**. In the supplementary table, it is shown that depressive symptoms are significantly associated with the physical health of empty-nesters (*p* <.05). This is consistent with the results of the logistic regression model study and indicates that the findings above are robust and reliable.

## 5 Discussion

Empty-nesters are one of the vulnerable groups in Chinese society, and examining the impact of psychological health on the physical health of this group during a pandemic can help provide insights into their unique health needs and provide an important basis to improve China’s health system. Our study examines the relationship between the psychological health of empty-nesters and their physical health during the pandemic based on national data. The findings provide strong data support for policy makers aiming to optimize the health services for empty-nesters and improve their overall health. In addition, the study provides policy implications to respond to public health emergencies, emphasizing greater attention to the psychological health of the population during crisis management.

The COVID-19 pandemic has had a significant impact on the psychological and physical health of elderly individuals. Studies indicate that the elderly are more vulnerable to the psychological effects of isolation, leading to increased rates of anxiety, depression, and loneliness during the pandemic (28, 29). This study, using the cross-sectional data, analyzed and summarized the extent to which depressive symptoms affect physical health from different perspectives. Our study results showed that 73.7% of the population aged 60 and above are elderly people who do not live with their children, which suggests an increased need to raise awareness for the health concerns of this group. Health not only refers to normal biological mechanisms but also to healthy psychological status, self-esteem, and cognitive well-being. In our study, 44.79% of empty-nesters suffer from depressive symptoms, which was lower than the rates in the world reported by Liu Q et al. (49.86%) and higher than the rates in China reported by Zhou L (39.65%) (30, 31). About half of the empty-nesters suffered from chronic diseases—specifically, 42.02% of the elderly were suffering from chronic diseases, which was higher than those in Brazil (37.3%) (32). We found that only 21.22% of empty-nesters consider their health to be good. It was notable that it was lower than the results among the elderly Korean Americans (66.8%), the elderly in Canada (72.5%), and those 40–59-year-old Brazilians (59.3%) (33).

Our study also found that empty-nesters who experienced depressive symptoms reported poorer SRH. According to Beck’s cognitive model of depression (34), the link between depression and worsening SRH can be explained by how individuals with depression tend to focus on negative stimuli and interpret their surroundings in a pessimistic light. People suffering from depression often exhibit a biased attention toward negative events and develop negative self-perceptions. In the case of older adults with depression, they typically emphasize physical complaints while downplaying the emotional aspects of their psychological health issues (35).

The rates of chronic disease were 1.45 times higher in empty-nesters with depressive symptoms compared to those without depressive symptoms. According to previous studies, depression is associated with an increased prevalence of chronic diseases (36), and it is also one of the predictors of 30- and 90-day readmissions in older adults with asthma and chronic obstructive pulmonary disease (37). The first and most important factor in how chronic illnesses cause or exacerbate depression is the burden of the illness and its impact on people’s quality of life (38, 39). Some authors suggest that antidepressant medications may contribute to the patient’s risk of becoming overweight and developing diabetes (40).

Similar to previous research, depressive symptoms are a necessary predictor of BADLs disability (41). Some researchers have found that individuals who feel depressed are more likely to report difficulties with BADLs abilities (42). Depressive symptoms can influence BADLs difficulties through a variety of potential mechanisms—for example, depression has been associated with increased markers of inflammation, causing and exacerbating inflammatory processes that increase the risk of subsequent BADLs impairment (43). Somatic symptoms of depression, such as fatigue and pain, may also lead to a decline in physical functioning in older adults, impairing BADLs ability (44, 48). In addition, depressive symptoms can accelerate the development of BADLs disorders through social isolation and psychological mechanisms (45, 46).

Interestingly, our study found that married empty-nesters reported worse SRH compared to unmarried empty-nesters. A study found that unmarried elderly hypertensive patients have better SRH compared with married elderly hypertensive patients, possibly because of reduced long-term family conflict (47). In addition, unmarried empty-nesters tended to be more cautious about maintaining their health since they did not have a spouse or children by their side. Meanwhile, our research found that rural empty-nesters had fewer chronic diseases compared to those living in urban areas. Analyzing the reasons, for chronic diseases through self-reported statistics, the rural elderly rarely goes to the hospital for diagnosis, and even if they suffer from chronic diseases, they are not aware of them. Limited BADLs also differs by gender, and women are found to be more likely to have limited BADLs than men (49).

As for health behaviors, our study found an association between SRH, chronic disease, and BADLs ability among empty-nesters and their lack of sleep. Our results indicated that a longer sleep duration is associated with a lower likelihood of poor SRH among empty-nesters. This is similar to the findings of previous studies, but there is no evidence of a relationship between too much sleep and chronic disease. Previous studies have shown a bidirectional link between short sleep duration and chronic disease multimorbidity (50). We found that alcohol consumption is associated with better SRH. The reason may be that regular drinkers have developed the habit of drinking moderately in their lives in a way that relieves stress and keeps them happy. Studies have shown that moderate alcohol consumption is associated with better health outcomes (51, 52). In our study, empty-nesters who quit smoking reported worse SRH than those who were still smoking, which was consistent with another study in China (53). It reveals that people who quit smoking due to illness are less likely to report good SRH.

Based on our research findings, empty-nesters with depressive symptoms significantly impact their physical health. Psychological health deserves more attention, especially during the COVID-19 pandemic. Therefore, we recommend that the Chinese government take more focused actions to improve the well-being of this group. The civil administration should, firstly, implement national campaigns to raise awareness about the importance of psychological health and the benefits of regular physical activity for the elderly, particularly those living alone, such as subsidized local fitness classes or home-based exercise resources. Additionally, family members should be encouraged to provide more emotional and practical support by maintaining regular contact with elderly parents (54). To address the challenges faced by elderly individuals with geographically distant children, the government could consider providing biannual subsidies to help cover transportation costs for family visits. Secondly, creating community-driven initiatives or providing family caregiver training could help families better understand the signs of depression and the importance of psychological support. Finally, social support networks are essential to address depressive symptoms. The government should invest in building more community spaces and leisure facilities that cater specifically to older adults, such as social clubs or group exercise sessions, to reduce isolation and promote social interaction (55, 56). For those with limited access to on-site medical care or those living in rural areas, healthcare professionals in local advanced hospitals should provide regular telehealth services for both physical health check-ups and psychological counseling, which could be scheduled bi-weekly or monthly.

### 5.1 Limitation

There were several limitations in this study to address in further research. Firstly, this was a cross-sectional study, so the findings highlight associations rather than causative conclusions. It is difficult to establish a causal association among depressive symptoms, SRH, chronic disease, and BADLs, for which further cohort studies are needed to confirm. Moreover, this study only relied on the CSED-10 scale to assess the respondents’ psychological health over a week, which did not allow for accurate inferences about an individual’s long-term status. Longitudinal data could be used in the future to look at the impact on physical health of people with depressive symptoms. Last but not least, the respondents’ self-reports of health status may be distorted by social expectations and recall biases. To reduce these biases, future studies should use more objective measures of health and collect data from a wider range of sources.

## 6 Conclusion

Our study found that depressive symptoms are an important factor associated with physical health among empty-nesters. Compared to empty-nesters with no depressive symptoms, empty-nesters with depressive symptoms are found to have a higher risk of poor SRH, chronic diseases, and limited BADLs. Our results highlighted that psychological depression is significantly associated with physical health. It is still essential to pay attention to psychological depression and avoid health problems caused by negative psychological health.

## Data Availability

Publicly available datasets were analyzed in this study. This data can be found here: https://charls.pku.edu.cn/.
