# Supplementary material for: Association between psychological depression and physical health in Chinese empty-nesters during the COVID-19 pandemic
Source: Front Psychiatry. 2025 Feb 18;16:1473783. doi: 10.3389/fpsyt.2025.1473783 (PMC11876410; doi:10.3389/fpsyt.2025.1473783)
Supplement: Supplementary file 1 [file Table1.pdf]

Table 1. Target population selection criteria

Question number	Contents	Answer	Selected
(CA014)	"How many months have you lived with your children in the past year?"	1-12	[0-1]
(CA015)	"How often do you see your children when yo u don't live with the m?"	Classified variable	More than one month
(BA018)	"For the first half of this year, how many days did you live by yourself?"	Numeric variable	[30-182]
(BA019)	"For the first half of the year, how many days did you live with just your spouse/partner (no one else but your spouse)?"	Numeric variable	[30-182]

Noted: The full questionnaire has been placed in Supplement material 2.

Table 2. Distribution of basic characteristics of empty-nesters among different depressive symptoms（N=7835）

Variables	Depressive symptoms				$χ^2$	*p*
	No		Yes			
	n	%	n	%		
Gender					236.05	<0.001
Male	2007	65.12	1075	34.88		
Female	1526	46.01	1791	53.99		
Age (years)					17.81	<0.001
60-65	1175	58.75	825	41.25		
65-70	1056	54.72	874	45.28		
70-75	659	51.61	618	48.39		
≥75	643	53.94	549	46.06		
Marital status					97.41	<0.001
Married	2688	59.13	1858	40.87		
Non-married	845	45.60	1008	54.40		
Residential area					138.84	<0.001
Urban	1382	65.65	723	34.35		
Rural	2146	50.06	2141	49.94		
Children's support					28.67	<0.001
No support	1091	58.19	784	41.81		
Low support	1443	51.44	1362	48.56		
High support	999	58.12	720	41.88		
Educational level					280.17	<0.001

Illiteracy	739	42.23	1011	57.77		
≤Primary school	1475	53.73	1270	46.27		
Middle school	663	67.86	314	32.14		
≥High school	439	75.43	143	24.57		
Per capita household expenditure					67.39	<0.001
Low	591	49.46	604	50.54		
Lower	635	49.65	644	50.35		
Middle	718	55.88	567	44.12		
Higher	760	57.40	564	42.60		
Highest	868	63.05	484	36.95		
Sleeping time					310.54	<0.001
6-8	1538	65.45	812	34.55		
<6	1098	42.09	1511	57.91		
≥8	897	62.29	543	37.71		
Smoking status					102.45	<0.001
Current	981	61.78	607	38.22		
Former	657	64.22	366	35.78		
Never	1895	50.03	1893	49.97		
Alcohol consumption					112.26	<0.001
No	2144	50.52	2100	49.48		
Yes	1389	64.45	766	35.55		

Table 3. Association analysis of psychological health and physical health among empty-nesters after simple random deletion method（N=7405）

**Variables**	**Physical health**											
	Good Self-rated health				Chronic disease				BADLs			
	OR	95%CI		*P*	OR	95%CI		*P*	OR	95%CI		*P*
		Lower	Upper			Lower	Upper			Lower	Upper	
**Psychological health**												
Depressive symptoms												
No	1.000				1.000				1.000			
Yes	0.288	0.246	0.337	<0.001	1.471	1.312	1.649	<0.001	3.135	2.754	3.568	<0.001
**Control variables**												
Gender												
Male	1.000				1.000				1.000			
Female	0.892	0.730	1.089	0.261	1.016	0.863	1.196	0.851	1.451	1.201	1.754	<0.001
Age (years)												
60-65	1.000				1.000				1.000			
65-70	0.880	0.742	1.044	0.143	1.120	0.974	1.287	0.113	1.148	0.974	1.354	0.100
70-75	0.854	0.701	1.039	0.115	1.196	1.022	1.399	0.026	1.519	1.268	1.819	<0.001
≥75	0.896	0.732	1.097	0.288	0.993	0.841	1.172	0.934	1.822	1.512	2.195	<0.001
Marital status												
Non-married	1.000				1.000				1.000			
Married	0.750	0.641	0.878	<0.001	0.977	0.861	1.108	0.717	1.042	0.905	1.199	0.565
Residential area												
Urban	1.000				1.000				1.000			
Rural	0.906	0.774	1.061	0.219	0.866	0.762	0.984	0.027	1.327	1.141	1.543	<0.001
Children's support												
No support	1.000				1.000				1.000			
Low support	0.958	0.816	1.125	0.603	0.996	0.874	1.134	0.946	1.176	1.012	1.366	0.034
High support	1.013	0.848	1.209	0.889	1.003	0.868	1.159	0.968	1.030	0.869	1.220	0.734
Educational level												
Illiteracy	1.000				1.000				1.000			

≤Primary school	0.651	0.548	0.772	<0.001	1.032	0.902	1.182	0.645	0.922	0.795	1.068	0.279
Middle school	0.759	0.608	0.947	0.015	1.046	0.870	1.258	0.631	0.788	0.634	0.978	0.031
≥High school	0.777	0.596	1.014	0.063	1.079	0.862	1.351	0.506	0.683	0.515	0.905	0.008
Per capita household expenditure												
Low	1.000				1.000				1.000			
Lower	0.986	0.800	1.216	0.897	0.927	0.780	1.102	0.391	1.009	0.833	1.222	0.925
Middle	0.813	0.656	1.009	0.060	1.094	0.920	1.301	0.312	0.978	0.803	1.190	0.821
Higher	0.759	0.610	0.945	0.014	1.149	0.964	1.369	0.121	1.162	0.954	1.417	0.136
Highest	0.840	0.672	1.050	0.126	1.205	1.004	1.446	0.045	1.250	1.015	1.541	0.036
Sleeping time												
6-8	1.000				1.000				1.000			
<6	0.699	0.596	0.820	<0.001	1.186	1.047	1.344	0.007	1.520	1.318	1.752	<0.001
≥8	1.214	1.027	1.434	0.023	0.992	0.857	1.149	0.916	0.987	0.828	1.175	0.879
Smoking status												
Current	1.000				1.000				1.000			
Former	0.675	0.542	0.839	<0.001	1.400	1.179	1.662	<0.001	1.300	1.061	1.593	0.011
Never	1.091	0.894	1.330	0.393	1.069	0.905	1.264	0.432	0.941	0.773	1.145	0.544
Alcohol consumption												
No	1.000				1.000				1.000			
Yes	1.437	1.235	1.673	<0.001	0.820	0.723	0.929	0.002	0.931	0.805	1.078	0.338

Table 4. Association analysis of psychological health and physical health among empty-nesters by probit model regression（N=7835）

**Variables**	Good Self-rated health			Chronic disease			BADLs		
	Coefficient	Robust std.err.	*P*	Coefficient	Robust std.err.	*P*	Coefficient	Robust std.err.	*P*
Depressive symptoms									
Yes	-0.712	0.042	<0.001	0.230	0.035	<0.001	0.677	0.037	<0.001
Gender									
Female	-0.054	0.058	0.356	0.010	0.050	0.844	0.209	0.054	<0.001
Age (years)									
65-70	-0.077	0.048	0.113	0.081	0.042	0.058	0.106	0.047	0.025
70-75	-0.101	0.056	0.072	0.127	0.048	0.008	0.279	0.053	<0.001
≥75	-0.067	0.058	0.248	-0.003	0.051	0.960	0.381	0.054	<0.001
Marital status									
Married	-0.167	0.045	<0.001	-0.026	0.039	0.508	0.022	0.042	0.592
Residential area									
Rural	-0.050	0.044	0.259	-0.086	0.039	0.029	0.165	0.043	<0.001
Children's support									
Low support	-0.021	0.046	0.645	-0.013	0.039	0.738	0.081	0.043	0.063
High support	0.018	0.050	0.716	0.003	0.044	0.949	0.004	0.049	0.936
Educational level									
≤Primary school	-0.241	0.049	<0.001	0.033	0.042	0.430	-0.074	0.044	0.094
Middle school	-0.143	0.064	0.026	0.025	0.057	0.653	-0.166	0.063	0.009
≥High school	-0.163	0.077	0.035	0.051	0.069	0.457	-0.231	0.079	0.004
Per capita household expenditure									

Lower	-0.018	0.060	0.763	-0.055	0.052	0.300	-0.019	0.056	0.729
Middle	-0.109	0.061	0.075	0.039	0.053	0.462	-0.037	0.057	0.522
Higher	-0.152	0.062	0.014	0.092	0.054	0.087	0.065	0.058	0.263
Highest	-0.086	0.064	0.178	0.129	0.056	0.021	0.110	0.061	0.072
Sleeping time									
<6	-0.210	0.045	<0.001	0.112	0.038	0.004	0.230	0.042	<0.001
≥8	0.135	0.048	0.005	0.016	0.045	0.725	-0.023	0.050	0.646
Smoking status									
Former	-0.219	0.062	<0.001	0.195	0.052	<0.001	0.142	0.059	0.016
Never	0.041	0.057	0.482	0.047	0.051	0.358	-0.041	0.056	0.466
Alcohol consumption									
Yes	0.200	0.043	<0.001	-0.116	0.038	0.003	-0.045	0.042	0.287
