# Supplementary material for: Association between psychological depression and physical health in Chinese empty-nesters during the COVID-19 pandemic
Source: Front Psychiatry. 2025 Feb 18;16:1473783. doi: 10.3389/fpsyt.2025.1473783 (PMC11876410; doi:10.3389/fpsyt.2025.1473783)
Supplement: Supplementary file 2 [file DataSheet1.pdf]

China Health and Retirement Longitudinal Study

Wave 5 (2020) Questionnaire

VersionID: 20231106

Nov. 2023

National School of Development
Institute of Social Science Survey
Peking University

Contents

CV Coverscreen 1

CV1. Household of Two or One Person 2

CV2. Marriage and Separation 2

CV3. Marital Status of Main Respondent 2

Auxiliary Variable Definition 3

B Demographic Backgrounds 7

B.Proxy Mode Confirmation 8

BA.Demographics 8

Auxiliary Variable Definition 11

C Family Information 15

C. Proxy Mode Confirmation 16

CA. Children Information 16

CB. Household Member Information 20

CC. Family Connections During the Chinese New Year 21

Auxiliary Variable Definition 22

D Health Status and Functioning 27

D.Proxy Mode Confirmation 28

DA.Health Status (1) 28

DA.Health Status (2) 30

DA.Health Status (3) 32

DB. Functional Limitations and Helpers (1) 34

DB. Functional Limitations and Helpers (2) 38

DB. Functional Limitations and Helpers (3) 39

DC.Cognition and Depression (1) 41

DC.Cognition and Depression (2) 43

Auxiliary Variable Definition 49

F Work and Retirement 55

F. Proxy Mode Confirmation 56

FA. Work Status (1): General 56
FA. Work Status (2): Change 57
FB. Self-Employed Agricultural Work 58
FC. Employed Work (1): General 59
FC. Employed Work (2): Wages 62
FD. Non-Agricultural Self-Employed Work 64
FE. Side Jobs 64
FF. Job Search 64
FG. COVID Impact 66
FH. Retirement 67
Auxiliary Variable Definition 68

G Income and Expenditure **73**
G1 Household Income and Expenditure 74
G1. Proxy Mode Confirmation 74
GB.Other Household Member's Wage Income and Individual-based Transfers 74
GC.Household Agricultural Income and Expenditure 76
GD.Self-Employed Activities 77
GE. Household Public Transfer Income 79
GF.Household Living Expenditure 81
I.Housing Characteristics 84
G2 Individual Income 88
G2.Proxy Mode Confirmation 88
GA.Individual Income 88
Auxiliary Variable Definition 91

V COVID **95**
V. Proxy Mode Confirmation 96
VA. Awareness 96
VB. Infection and Quarantine 97
VC. Activities 99
VD. Restrictions in the Residence Area 104
Auxiliary Variable Definition 105

EX Exit **107**
EXB. Demographics 108
EXC. Family 110
EXD. Health Status and Funtioning (1) 111

EXD. Health Status and Funtioning (2) 114
EXD. Health Status and Funtioning (3) 118
EXE. Health Care and Insurance(1) 119
EXE. Health Care and Insurance(2) 121
EXF. Work and Retirement 124
EXFN. Pension 125
EXG. Incomes, Expenditures and Assets 125
EXK. Funeral 129
EXV. COVID 130
VA. Cause of Death 133
Auxiliary Variable Definition 133

Appendix Function Descriptions **141**
A. Functions in the Questions 142
B. Functions in Auxiliary Variables 142

This page intentionally left blank

CV Coverscreen

CV1. Household of Two or One Person

CV001 Of people on the following list, who is answering the cover screen?

1. [ZRName1]
2. [ZRName2]
3. Informant, name: ______ (**CV001_1**), relationship with [ZRName1] ______ (**CV001_2**)

CV002 Is [ZRName1] alive?

【**IWER**: If [ZRName1] is the one who answers the cover screen, choose option 1 (alive) without asking questions. Use four digits to represent the year.】

1. Alive
2. Died, date of death was: Year ______ [hc$([2011, 2020], int, \varnothing)$, sc$([\text{ZIWYear}, 2020], int, \varnothing)$] (**CV002_1**) Month ______ [hc$([1, 12], int, -1)$] (**CV002_2**) Day ______ [hc$([1, 31], int, -1)$] (**CV002_3**)

CV003 Is [ZRName2] still alive?

【**IWER**: If [ZRName2] is the one who answers the cover screen, choose option 1 without asking questions. Use four digits to represent the year.】

1. Alive
2. Died, date of death was: Year ______ [hc$([2011, 2020], int, \varnothing)$, sc$([\text{ZIWYear}, 2020], int, \varnothing)$] (**CV003_1**) Month ______ [hc$([1, 12], int, -1)$] (**CV003_2**) Day ______ [hc$([1, 31], int, -1)$] (**CV003_3**)

CV2. Marriage and Separation

CV004 Are [ZRName1] and [ZRName2] still in a marital relationship or living together as spouses?

1. [ZRName1] and [ZRName2] are still in marital relationship or living together as spouses
2. [ZRName1] and [ZRName2] have divorced
3. [ZRName1] and [ZRName2] have being separated for a long time and will not live together as spouses in the future

CV005 Are you more familiar with [ZRName1] or [ZRName2]?

1. [ZRName1]
2. [ZRName2]

CV3. Marital Status of Main Respondent

CV006 What is [XMainR]'s current marital status?

1. Married and living with spouse
2. Married, but temporarily not living with spouse due to work or other reasons
3. Separated and no longer living together as spouses
4. Divorced
5. Widowed
6. Never married

CV007 Does [XMainR] currently have a partner living together as a spouse?

1. Yes, his/her name: ______ (**CV007_1**)
2. No

CV008 What is the name of [XMainR]'s spouse? ______

Auxiliary Variable Definition

XRLive1 Whether the respondent1 is alive or not

```
if (equal("CV002", "1")) {
    add("XRLive1", "1")
}
if (equal("CV002", "2")) {
    add("XRLive1", "2")
}
```

XRLive2 Whether the respondent2 is alive or not

```
if (equal("CV003", "1")) {
    add("XRLive2", "1")
}
if (equal("CV003", "2")) {
    add("XRLive2", "2")
}
```

XBothAlive Combination of alive or dead conditions of two respondents

```
if equal("CV002","1") && (equal("CV003","1")) {
    add("XBothAlive", "1")
}
if equal("CV002","2") && (equal("CV003","2")) {
    add("XBothAlive", "2")
}
if equal("CV002","1") && (equal("CV003","2")) {
    add("XBothAlive", "3")
}
if equal("CV002","2") && (equal("CV003","1")) {
    add("XBothAlive", "4")
}
```

XMaritalStatus Marrital status of two-person household

```
if (equal("CV004", "1")) {
    add("XMaritalStatus", "1")
}
if (equal("CV004", "2") || equal("CV004", "3")) {
    add("XMaritalStatus", "2")
}
```

XMainR Name of XMainR

```
if (equal("CV_HType", "1") && equal("XBothAlive", "3")) {
    add("XMainR", value("ZRName1"))
    add("XRType1", "1")
}
if (equal("CV_HType", "1") && equal("XBothAlive", "4")) {
    add("XMainR", value("ZRName2"))
    add("XRType2", "1")
}
if (equal("CV_HType", "2") && equal(" XRLive1", "1")) {
    add("XMainR", "ZRName1")
    add("XRType1", "1")
}

if (equal("XMaritalStatus", "1")) {
    add("XMainR", "ZRName1")
    add("XRType1", "1")
```

```
        add("XMainRS", "ZRName2")
        add("XRType2", "1")
    }

    if (((equal("CV004", "2") || equal("CV004", "3")) && equal("CV001", "1")) ||
        ((equal("CV004", "2") || equal("CV004", "3")) && equal("CV001", "3") && equal("CV005", "1"))) {
        add("XMainR", "ZRName1")
        add("XRType1", "1")
        add("XRSplited2", "1")
    } else {
        add("XRSplited2", "0")
    }

    if (((equal("CV004", "2") || equal("CV004", "3")) && equal("CV001", "2")) ||
        ((equal("CV004", "2") || equal("CV004", "3")) && equal("CV001", "3") && equal("CV005", "2"))) {
        add("XMainR", "ZRName2")
        add("XRType2", "1")
        add("XRSplited1", "1")
    } else {
        add("XRSplited1", "0")
    }
```

XMainRS Name of XMainR's spouse

```
see above
```

XRType1 Type of ZRName1

```
see above
```

XRType2 Type of ZRName2

```
see above
```

XRSplited1 New household of ZRName1

```
if (((equal("CV004", "2") || equal("CV004", "3")) && equal("CV001", "2")) || ((equal("CV004", "2") ||
↪   equal("CV004", "3")) && equal("CV001", "3") && equal("CV005", "2"))) {
    add("XMainR", "ZRName2")
    add("XRType2", "1")
    add("XRSplited1", "1")
} else {
    add("XRSplited1", "0")
}
```

XRSplited2 New household of ZRName2

```
if (((equal("CV004", "2") || equal("CV004", "3")) && equal("CV001", "1")) || ((equal("CV004", "2") ||
↪   equal("CV004", "3")) && equal("CV001", "3") && equal("CV005", "1"))) {
    add("XMainR", "ZRName1")
    add("XRType1", "1")
    add("XRSplited2", "1")
} else {
    add("XRSplited2", "0")
}
```

XRDeathYear1 The year of ZRName1's death

```
add("XRDeathYear1", value("CV002_1"))
```

XRDeathMonth1 The month of ZRName1's death

```
add("XRDeathMonth1", value("CV002_2"))
```

XRDeathDate1 The day of ZRName1's death

```
add("XRDeathDate1", value("CV002_3"))
```

XRDeathYear2 The year of ZRName2's death

```
add("XRDeathYear2", value("CV003_1"))
```

XRDeathMonth2 The month of ZRName2's death

```
add("XRDeathMonth2", value("CV003_2"))
```

XRDeathDate2 The day of ZRName2's death

```
add("XRDeathDate2", value("CV003_3"))
```

ZRName1 Name of respondent

ZRName2 Name of respondent

CV_HType Type of household. Value 1 means two person household, value 2 means one person household.

ZIWTime Last interview date.

This page intentionally left blank

B Demographic Backgrounds

B.Proxy Mode Confirmation

proxy_2 Interviewer record: Enter proxy mode?

1. Yes
2. No

BA.Demographics

BA001 Interviewer record the [XRName] 's gender

1. Male
2. Female

BA002 We recorded [XRName] 's gender as [XR18Gender] in the last interview, this time, your gender is [XR20Gender], please confirm [XRName] 's gender again

1. Male
2. Female

BA003 [XRName] 's actual date of birth according to solar calendar: ______ [hc$([1900, 2000], int, \varnothing)$] (**BA003_1**) Year ______ [hc$([1, 12], int, -1)$] (**BA003_2**) Month ______ [hc$([1, 31], int, -1)$] (**BA003_3**) Day

【**IWER**: Mark the year using four digits. Please enter "-1" if R cannot answer the month and day.】

BA004 Interviewer records the current address:

______ (**BA004_1**) province/city/county/district
______ (**BA004_2**) township/village/neighborhood
______ (**BA004_3**) building/house number

BA005 interviewer records the type of current address.

1. Family housing
2. Workplace
3. Other, please specify: ______ (**BA005_1**)

BA006 Where does [XRName] live now? Excluding temporary business trip/travel or visit relatives and friends.

1. Address of interview: [XRSurveyAdd]
2. Not address of interview: Chinese mainland ______ (**BA006_1**) province/city/county/district ______ (**BA006_2**) township/village/neighborhood ______ (**BA006_3**) building/house number
3. Hong Kong, China
4. Macao, China
5. Taiwan, China
6. Abroad: ______ (**BA006_4**)

BA007 What is the type of [XRName]'s residential address? [XRResidenceFull]

1. Family housing
2. Nursing home
3. Hospital

4. Other, please specify ______ (**BA007_1**)

BA008 [XRResidenceQuestion] ?

1. The center of city/town
2. Combination zone between urban and rural areas
3. Village
4. Special area

BA009 What is the type of [XRName]'s HuKou?

1. Agricultural HuKou
2. Non-agricultural HuKou
3. Unified residence HuKou
4. Do not have HuKou

BA010 What's the highest level of education [XRName] has received? Excluding adult education.

1. Illiterate
2. Not completing primary school
3. Sishu/Old-style private school
4. Primary school
5. Middle school
6. High school
7. Vocational school
8. Two-/Three-year college/associate degree
9. Four-Year college/bachelor's degree
10. Master's degree
11. Doctoral degree/Ph.D.

BA010_1 Is [XRName] literate?

1. Yes
2. No

BA011 What is [XRName]'s marital status?

1. Married and living with spouse
2. Married, but temporarily not living with spouse due to work or other reasons
3. Separated and no longer living together as spouses
4. Divorced
5. Widowed
6. Never married

BA012 Does [XRName] currently have a partner living together as a spouse?

1. Yes
2. No

BA013 Last year, how many months has [XRName] lived with his/her spouse? ______ [$\text{hc}([0, 12], real, \varnothing)$] Months

BA014 How often are social pension insurance benefits distributed?

【**IWER**: If R only know the receiving frequency, please record the minimum time to receive pension insurance benefits.】

1. One month
2. One quarter
3. Half a year
4. One year
5. More than one year: ______ [hc((1, 50), *real*, ∅)] (**BA014_1**) Year
6. Participate in social endowment insurance but haven't reached the age of receiving it
7. Didn't participate in any social endowment insurance

BA015 What type of social pension insurance did [XRName] participate in?

【**IWER**:If R don't know the type of social pension insurance, please consult his/her children or village cadres.】

1. Pension insurance for employees of state organs or public institutions
2. Worker's basic social endowment insurance
3. Social endowment insurance for non-working urban residents
4. New social endowment insurance for rural residents
5. Social endowment insurance for urban and rural residents
6. Other, please specify: ______ (**BA015_1**)

BA016 [XRName] What is the annual payment amount of social health insurance?

【**IWER**: Please enter "-1" if R cannot answer. Please record the last payment amount if the annual payment amount varies.】

1. Annual payment amount ______ [hc((0, 100000], *real*, −1)] (**BA016_1**) Yuan
2. Have health insurance but no payment required
3. No social health insurance

BA017 What's the type of social health insurance?

【**IWER**: If R don't know the type of social health insurance, please consult his/her children or village cadres.】

1. Urban employee medical insurance (yi-bao)
2. Urban and rural resident medical insurance
3. Urban resident medical insurance
4. New cooperative medical insurance (he-zuo-yi-liao)
5. Government medical insurance (gong-fei)
6. Other, please specify: ______ (**BA017_1**)

BA018 How many days does [XRName] live alone during the first half of this year? ______ [hc([0, 182], *int*, ∅)]

BA019 In the first half of this year, how many days does [XRName] live with his/her spouse only? ______ [hc([0, 182], *int*, ∅)]

BA020 In the first half of this year, [XRName] doesn't live with [XRLiveCovid] for [DayNumber] days, what extent is this caused by the COVID-19 epidemic? ______ [hc([0, 100], *int*, ∅)] %

【**IWER**: To what extent did the R live alone or with their spouses because of the COVID-19 epidemic.】

Auxiliary Variable Definition

XR20Gender Gender of respondent

```
if (equal("BA001", "1")) {
    add("XR20Gender", "male")
}
if (equal("BA001", "2")) {
    add("XR20Gender", "female")
}
```

XR18Gender Gender registered in 2018

```
if (equal("ZRGender", "1")) {
    add("XR18Gender", "male")
}
if (equal("ZRGender", "2")) {
    add("XR18Gender", "female")
}
```

XRGender Confirmed gender

```
if (!empty("ZRGender") && equal("BA001", value("ZRGender"))) {
    add("XRGender", value("BA001"))
}
if (!empty("ZRGender") && !equal("BA001", value("ZRGender"))) {
    add("XRGender", value("BA002"))
}
if (empty("ZRGender")) {
    add("XRGender", value("BA001"))
}
```

XRAge Age

```
if (empty("ZRBirthYear") || equal("XRType", "2")) {
    add("XRAge", 2020 - value("BA003_1"))
}
if (equal("XRType", "1") && !empty("ZRBirthYear")) {
    add("XRAge", 2020 - value("ZRBirthYear"))
}
```

XRResidenceFull Residential address: Province/City/County/Township

```
if (equal("BA006", "1")) {
    add("XRResidenceFull",value("BA004_1")+value("BA004_2"))
}
if (equal("BA006", "2")) {
    add("XRResidenceFull",value("BA006_1")+value("BA006_2"))
}
if (equal("BA006", "3")) {
    add("XRResidenceFull", "Hong Kong, China")
}
if (equal("BA006", "4")) {
    add("XRResidenceFull", "Macao, China")
}
if (equal("BA006", "5")) {
    add("XRResidenceFull", "Taiwan, China")
}
if (equal("BA006", "6")) {
    add("XRResidenceFull", "Abroad:"+value("BA006_4"))
}
```

XRResidenceCounty Residential address: Province, City, County

```
if (equal("BA006", "1")) {
    add("XRResidenceCounty",value("BA004_1"))
}
if (equal("BA006", "2")) {
    add("XRResidenceCounty",value("BA006_1"))
}
```

XRResidenceCommunity Residential address: County, Township, Village/Neighborhood

```
if (equal("BA006", "1")) {
    add("XRResidenceCommunity",value("BA004_2"))
}
if (equal("BA006", "2")) {
    add("XRResidenceCommunity",value("BA006_2"))
}
```

XRSurveyAdd Address of Interview

```
add("XRSurveyAdd", value("BA004_1")+value("BA004_2")+value("BA004_3"))
```

XRResidenceSurveyHome The place of residence is the place of interview, and it is a family residence.

```
if (equal("BA006", "1") && equal("BA005", "1")) {
    add("XRResidenceSurveyHome", "1")
}
if ((equal("BA006", "1") && !equal("BA005", "1")) || equal("BA006", "2") || equal("BA006", "3") ||
↪   equal("BA006", "4") || equal("BA006", "5") || equal("BA006", "6") ) {
    add("XRResidenceSurveyHome", "2")
}
```

XRResidenceQuestion Different questions of urban and rural categories.

```
if (equal("BA006", "1")) {
    add("XRResidenceQuestion", "Filled by the interviewer"+value("XRSurveyAddFull")+"is urban or rural")
}
if (equal("BA006", "2")) {
    add("XRResidenceQuestion", "When"+ value("XRName")+"living in"+value("XROtherInlandFull")+", mainly
    ↪  live in rural areas or cities")
}
```

XRSurveyAddFull Interview address: Province, City, County, Township, Village/Neighborhood

```
add("XRSurveyAddFull", value("BA004_1")+value("BA004_2"))
```

XROtherInlandFull Residential address: not interview address, Province, City, County, Township, Village/Neighborhood

```
if (equal("BA006", "2")) {
    add("XROtherInlandFull", value("BA006_1")+value("BA006_2"))
}
```

XRPartner Having a spouse or partner

```
if ((equal("BA011", "1") || equal("BA011", "2")) || ((equal("BA011", "3") || equal("BA011", "4") ||
↪   equal("BA011", "5") ||
    equal("BA011", "6")) && equal("BA012", "1"))) {
    add("XRPartner", "1")
}
if ((equal("BA011", "3") || equal("BA011", "4") || equal("BA011", "5") || equal("BA011", "6")) &&
↪   equal("BA012", "2")) {
    add("XRPartner", "2")
}
```

XRLiveCovid Different wording of the question.

```
if (equal("XRPartner", "1")) {
    add("XRLiveCovid", "Anyone except spouse")
}
if (equal("XRPartner", "2")) {
    add("XRLiveCovid", "Anyone")
}
```

DayNumber Days not living with other than spouse

```
if (equal("XRPartner", "1")) {
    add("DayNumber", value("BA018")+value("BA019"))
}
if (equal("XRPartner", "2")) {
    add("DayNumber", value("BA018"))
}
```

XRName Name of respondent

C Family Information

C. Proxy Mode Confirmation

proxy_3 Interviewer record: Enter proxy mode?

1. Yes
2. No

CA. Children Information

CA001 We would like to know the information about [XMainR] and [XMainRS]'s family members, including children, parents, and siblings. Who has a better idea about such information, [XMainR] or [XMainRS]?

1. [XMainR]
2. [XMainRS]

【**INTRO**: In the following section, we would like to ask some questions about [XFamilyR]'s children.】

CA002[i] Is [ZChildName[i]] (Gender: [XChildGenderDis[i]]) still alive?

1. Yes
2. No

CA003[i] When did [ZChildName[i]] pass away? ______ [hc([2011, 2020], int, ∅), sc([ZIWYear, 2020], int, ∅)] (**CA003_1[i]**) Year ______ [hc([1, 12], int, ∅)] (**CA003_2[i]**) Month ______ [hc([1, 31], int, −1)] (**CA003_3[i]**) Day

【**IWER**: Interviewer must fill in the year and month of death to generate logics of procedures. If R is unwilling to answer or does not remember the day of death, please input "-1" here.】

CA004[i] What's the leading cause of death for [ZChildName[i]]? ______

【**IWER**: If the cause of death is illness, please specify the kind of illness. For example, if it is cancer, the specific type of cancer should be recorded (gastric cancer, lung cancer, etc.); if it is infectious disease, please specify the disease (tuberculosis, dysentery, etc.); or if it is an accident, please give information on the accident type (car accident, fire, intoxication, etc.)】

CA005[i] When was [XChildPanAliveName[i]] born? ______ [hc([1910,ZIWYear], int, ∅), sc([1940,ZIWYear], int, ∅)] Year

【**IWER**: Mark the year using four digits. If R does not remember the birth year of his/her child clearly, you could calculate it from information about his/her child's age now, his/her child's death year and the age of death, or age of R when his/her child was born.】

CA006[i] What's [XChildPanAliveName[i]]'s gender?

1. Male
2. Female

CA007[i] Without taking continuing education into account, what's [XChildPanAliveName[i]]'s highest achieved education?

1. No formal education (illiterate)
2. Did not finish elementary school
3. Sishu/home school

4. Elementary school
5. Middle school
6. High school
7. Vocational school
8. Two/Three-Year College/Associate degree
9. Four-Year College/Bachelor's degree
10. Master's degree
11. Doctoral degree/Ph.D.
997. Don't know
999. Refuse

CA008[i] Is [XChildAliveName[i]] a student or does [XChildAliveName[i]] work? "Work" means being a farmer, doing work for salary, self-employed or assisting in family business without getting paid.

1. Working
2. Student
3. A student working part-timely or full-time worker while studying part-timely
4. Neither a student nor does he/she work, please specify what he/she has been doing ______ (**CA008_1[i]**)
997. Don't know
999. Refuse

CA009[i] What occupation does [XChildAliveName[i]]'s job belong to? That is, what sort of work does [XChildAliveName[i]] do? ______ (e.g.1: pastry chef in a restaurant; e.g.2: line winding assembly line worker)

CA010[i] What's [XChildPanAliveName[i]]'s current marital status?

1. Married with spouse present
2. Married but not living with spouse temporarily for reasons such as work
3. Separated
4. Divorced
5. Widowed
6. Never married
997. Don't know
999. Refuse

CA012[i] During last year, to which of the following buckets does [XChildCoupleDis[i]]'s aggregate annual income belong?

1. 0
2. Less than 2000 yuan
3. 2000-5000 yuan
4. 5000-10000 yuan
5. 10000-20000 yuan
6. 20000-30000 yuan
7. 30000-50000 yuan
8. 50000-100000 yuan
9. 100000-150000 yuan

10. 150000-200000 yuan
11. 200000-300000 yuan
12. More than 300000 yuan
997. Don't know
999. Refuse

CA013[i] What would you say about [XChildPanAliveName[i]]'s health status?

1. Very good
2. Good
3. Fair
4. Poor
5. Very poor
997. Don't know
999. Refuse

【**INTRO**: We would then like to ask some questions about interaction within [XFamilyR]'s family, including contact between, financial support and care-giving provided to and received from your/your spouse's children.】

CA014[i] During last year, how long had [XChildPanAliveName[i]] lived with [XFamilyRAndS]?

______ [hc([0, 12], *real*, ∅)] Month

【**IWER**: A short visit does not constitute "living together"; input 0 here if not living together at all and 12 if always living together.】

CA015[i] When [XChildPanAliveName[i]] is not living with [XFamilyRAndS], how often do [XFamilyRAndS] see [XChildPanAliveName[i]]?

1. Almost every day
2. 2-3 times a week
3. Once a week
4. Every two weeks
5. Once a month
6. Once every three months
7. Once every six months
8. Once a year
9. Almost never
10. Other

CA016[i] When [XChildPanAliveName[i]] is not living with [XFamilyRAndS], how often do [XFamilyRAndS] contact with [XChildPanAliveName[i]] on phone/by message/on Wechat/by mail/by email?

1. Almost every day
2. 2-3 times a week
3. Once a week
4. Every two weeks
5. Once a month
6. Once every three months
7. Once every six months

8. Once a year
9. Almost never
10. Other

【**INTRO**: From time to time, family members will provide help or support each other in various forms, and each form of support matters. And in the following section, here are some questions about the financial support [XFamilyRAndS] received from and provided to their children.】

CA017[i] During last year, what's the amount of financial support received from [XChildPanAliveName[i]] when he/she was not living with [XFamilyR]?
Money received in total ______ [hc($[0, \infty), int, -1$), sc($[0, 500000], int, -1$), ub($[-1], [100, 500, 1500, 5000, 20000]$)] (**CA017_1[i]**) Yuan, of which regular payment was ______ [hc($[0, \infty), int, -1$), sc($[0, 500000], int, -1$), ub($[-1], [100, 200, 1000, 3000, 10000]$)] (**CA017_2[i]**) Yuan (Regular payment includes providing living expenses, paying for water, electricity or telephone bill, paying for mortgage/rent or other forms of regular expenses);
In-kind payment received worth in total ______ [hc($[0, \infty), int, -1$), sc($[0, 500000], int, -1$), ub($[-1], [100, 500, 1500, 5000, 20000]$)] (**CA017_3[i]**) Yuan, of which regular payment was ______ [hc($[0, \infty), int, -1$), sc($[0, 500000], int, -1$), ub($[-1], [100, 200, 1000, 3000, 10000]$)] (**CA017_4[i]**) Yuan (for example, buying food, clothes or other stuff regularly for you).
【**IWER**: Please include the financial support received from [XChildPanAliveName[i]]'s children.
Regular payments are payments occurring monthly, quarterly, semi-annually, annually, in cash or in-kind, at some fixed points of time.
The amount of regular payment should not exceed the total amount of financial support received.
If nothing received, input 0 here; if an answer of "don't know" is given or no answer is provided, input "-1" here.】

CA018[i] During last year, what's the amount of financial support provided to [XChildPanAliveName[i]] when he/she was not living with [XFamilyR]?
Money provided in total ______ [hc($[0, \infty), int, -1$), sc($[0, 500000], int, -1$), ub($[-1], [100, 500, 1500, 5000, 20000]$)] (**CA018_1[i]**) Yuan, of which regular payment was ______ [hc($[0, \infty), int, -1$), sc($[0, 500000], int, -1$), ub($[-1], [100, 200, 1000, 3000, 10000]$)] (**CA018_2[i]**) Yuan (Regular payment includes providing living expenses, paying for water, electricity or telephone bill, paying for mortgage/rent or other forms of regular expenses);
In-kind payment provided worth in total ______ [hc($[0, \infty), int, -1$), sc($[0, 500000], int, -1$), ub($[-1], [100, 500, 1500, 5000, 20000]$)] (**CA018_3[i]**) Yuan, of which regular payment was ______ [hc($[0, \infty), int, -1$), sc($[0, 500000], int, -1$), ub($[-1], [100, 200, 1000, 3000, 10000]$)] (**CA018_4[i]**) Yuan (For example, buying food, clothes or other stuff regularly for you).
【**IWER**: Please include the financial support provided to [XChildPanAliveName[i]]'s children.
Regular payments are payments occurring monthly, quarterly, semi-annually, annually, in cash or in-kind, at some fixed points of time.
The amount of regular payment should not exceed the total amount of financial support provided.
If nothing provided, input 0 here; if an answer of "don't know" is given or no answer is provided, input "-1" here.】

CA019[i] Is the relationship between [XFamilyRAndS] and [XChildPanAliveName[i]] affected by the COVID-19 pandemic this year?

1. Yes, our relationship got better

2. Yes, our relationship got worse
3. No influence

CA020[i] Does the pandemic after the Chinese New Year affect [XChildCoupleDis[i]]'s income level? Is their income increased, decreased, or the same as before?

【**IWER**: If an answer of "don't know" is given or no answer is provided, input "-1" here; conditions permitting, the interviewer can ask R's children about the influence of the pandemic on their income.】

1. Increased, by approximately ______ [hc($(0,\infty)$, int, -1), sc($(0,100]$, int, -1)] (**CA020_1[i]**) percent
2. Decreased, by approximately ______ [hc($(0,100]$, int, -1)] (**CA020_2[i]**) percent
3. No influence

997. Don't know
999. Refuse

CB. Household Member Information

【**INTRO**: We would like to know some information on your household members in addition to [XFamilyRAndS]. By definition here, a household member is someone who lives together with [XFamilyRAndS], and with whom [XFamilyRAndS] would share family income and expenses.】

CB001 Of all people listed here, which are [XFamilyRAndS]'s household members? (Select All That Apply)

【**IWER**: By definition here, a household member is someone who lives together with R, and with whom R share family income and expenses. During on-site interview, a particular difficulty is to determine whether a child is a household member of the family R or not. A trick that could be used during on-site interview is to inquire R whether the child has been financially independent from him/her.】

1-25. [XChildAliveName[i]]
99. None of people in above list

[conflict($99, [99]^c$)]

【**INTRO**: [XIntroCB002]】

CB002 [XHHOtherDis] is there anyone else who is [XFamilyRAndS]'s household member? Please check the box and fill in the name.

1-10. ______ (**CB002_1[i]**)
99. No other household member

[conflict($99, [99]^c$)]

CB003[i] Household member [XHHOtherMemberName[i]]'s gender is?

1. Male
2. Female

CB004[i] How old is [XHHOtherMemberName[i]]? ______ [hc($[0,120]$, int, -1), sc($[0,100]$, int, -1)] Age

【**IWER**: If an answer of "don't know" is given or no answer is provided, input "-1" here.】

CB005[i] [XHHOtherMemberName[i]] is [XFamilyR]'s?

1. Daughter-in-law/son-in-law
2. Grandchild
3. Brother-in-law/sister-in-law
4. Father
5. Mother
6. Mother-in-law
7. Father-in-law
8. Child
9. Sibling
10. Other kind of relative, please specify ______ (**CB005_1[i]**)

CB006[i] Which child of [XFamilyR] has [XHHOtherMemberName[i]] been married with?

【**IWER**: Load names of all children alive. If this household member is the spouse of a deceased child, please specify the child's name in the text box.】

1-25. [XChildAliveName[i]]
99. Other, please specify the name ______ (**CB006_1[i]**)

CB007[i] Who is [XHHOtherMemberName[i]]'s parent?

【**IWER**: Load names of all children alive. If this household member is the child of a deceased child, please specify the deceased child's name in the text box.】

1-25. [XChildAliveName[i]]
99. Other, please specify the name ______ (**CB007_1[i]**)

CC. Family Connections During the Chinese New Year

【**INTRO**: In the following section, we would like to ask some questions about the impact of the COVID-19 pandemic on the reunion of [XFamilyR] with other family members.】

CC001 During the past Chinese New Year, were any of [XFamilyR]'s children or grandchildren unable to return home due to the COVID-19 pandemic?

1. Yes, there were ______ [hc$([1,15], int, \varnothing)$, sc$([1,8], int, \varnothing)$] (**CC001_1**) children and grandchildren unable to return home for the New Year
2. No

CC002 During the past Chinese New Year, were there any relatives and friends unable to come and visit [XFamilyR] due to the COVID-19 pandemic?

1. Yes
2. No

CC003 Compared with previous years, did [XFamilyRAndS] receive less money from the younger generation, relatives, and friends during the Chinese New Year?

1. Yes, about ______ [hc$((0,100000], int, \varnothing)$, sc$((0,50000], int, \varnothing)$] (**CC003_1**) Yuan less than previous years
2. No

CC004 During the Chinese New Year, the older generation usually gives out red envelopes

(Hong Bao) to the younger generation. Did [XFamilyRAndS] spend less money on giving out red envelopes (Hong Bao) this New Year compared with previous years?

1. Yes, about ______ [hc($(0,100000], int, \varnothing$), sc($(0,50000], int, \varnothing$)] (**CC004_1**) Yuan less than previous years
2. No

CC005 Did [XFamilyRAndS] mostly live together in January of the Lunar calendar (Zhengyue) this year?

1. Yes
2. No

CC006[i] With whom did [XCouLiveList[i]] live in January of the Lunar calendar (Zhengyue) this year? (Select All That Apply)

【**IWER**: Please include short visits】

1-25. [XChildPanAliveName[i]]
26-35. [XHHOtherMemPreload[i]]
99. None of people in above list

[conflict($99, [99]^c$)]

CC007[i] In addition to people mentioned before, who else lived with [XCouLiveList[i]] in January of the Lunar calendar (Zhengyue) this year? (Select All That Apply)

【**IWER**: Please include short visits.】

1. ______ (**CC007_1[i]**)
2. ______ (**CC007_2[i]**)
3. ______ (**CC007_3[i]**)
4. ______ (**CC007_4[i]**)
5. ______ (**CC007_5[i]**)
6. ______ (**CC007_6[i]**)
7. ______ (**CC007_7[i]**)
8. ______ (**CC007_8[i]**)
9. ______ (**CC007_9[i]**)
10. ______ (**CC007_10[i]**)
99. No other people

[conflict($99, [99]^c$)]

CC008[i] Compared with previous years, were there more or fewer people living with [XCouLiveList[i]] in January of the Lunar calendar (Zhengyue) this year?

1. More people
2. Fewer people
3. No difference

Auxiliary Variable Definition

XFamilyR Name of the family respondent

```
if (!empty("XMainRS")) {
    if (equal("CA001", "1")) {
        add("XFamilyR", value("XMainR"))
```

```
            add("XFamilyS", value("XMainRS"))
        } else if (equal("CA001", "2")) {
            add("XFamilyR", value("XMainRS"))
            add("XFamilyS", value("XMainR"))
        }
    } else {
        add("XFamilyR", value("XMainR"))
        add("XFamilyS", "")
    }
```

XFamilyS Name of spouse of the family respondent

```
see above
```

XFamilyRAndS Automatically preload [Name of the family respondent] and [Name of spouse of the family respondent] in corresponding questions. If the respondent has a spouse, show ”[Name of the family respondent] and [Name of spouse of the family respondent]”, otherwise only show the name of the family respondent.

```
if (!empty("XFamilyS")) {
    add("XFamilyRAndS", value("XFamilyR")+" and "+value("XFamilyS"))
} else {
    add("XFamilyRAndS", value("XFamilyR"))
}
```

XChildNum Number of children whose names are not null

```
add("XChildNum", "0")
for (var i = 1; i <= value("ZChildNum"); i++) {
    if (!empty("ZChildName[i]")) {
        add("XChildNum", value("XChildNum")+1)
    }
}
```

XChildGenderDis Show preloaded gender in corresponding questions

```
if (equal("ZChildGender[i]", "1")) {
    add("XChildGenderDis[i]", "Male")
} else if (equal("ZChildGender[i]", "2")) {
    add("XChildGenderDis[i]", "Female")
} else {
    add("XChildGenderDis[i]", "Missing")
}
```

XChildAlive Whether the child is still alive when interviewed

```
if (equal("CA002[i]", "1")) {
    add("XChildAlive[i]", "1")
} else if (equal("CA002[i]", "2")) {
    add("XChildAlive[i]", "0")
}
```

XChildAliveName Name of child alive when interviewed, null for deceased children

```
if (equal("XChildAlive[i]", "1")) {
    add("XChildAliveName[i]", value("ZChildName[i]"))
}
```

XChildAliveNum Number of children alive

```
add("XChildAliveNum", "0")
for (var i = 1; i <= value("ZChildNum"); i++) {
    if (!empty("ZChildName[i]") && equal("XChildAlive[i]", "1")) {
        add("XChildAliveNum", value("XChildAliveNum")+1)
    }
}
```

XChildPanAliveName Name of child alive when the pandemic started, null for deceased children

```
if (equal("XChildPanAlive[i]", "1")) {
    add("XChildPanAliveName[i]", value("ZChildName[i]"))
    add("XChildPanAliveNum", value("XChildPanAliveNum")+1)
}
```

XChildPanAliveNum Number of children alive when the pandemic started

```
see above
```

XChildPanAlive Whether the child was still alive when the pandemic started

```
if (equal("XChildAlive[i]", "1")) {
    add("XChildPanAlive[i]", "1")
} else if (greater("2019", "CA003_1[i]", true)) || (equal("CA003_1[i]", "2020") && equal("CA003_2[i]",
↪  "1")) {
    add("XChildPanAlive[i]", "0")
} else if (equal("CA003_1[i]", "2020") && greater("CA003_2[i]", "1", false)) {
    add("XChildPanAlive[i]", "1")
}
```

XChildGender Child's gender

```
if (!empty("ZChildGender[i]")) {
    add("XChildGender[i]", value("ZChildGender[i]"))
} else {
    add("XChildGender[i]", value("CA006[i]"))
}
```

XChildBirth Child's year of birth

```
if (!empty("ZChildBirth[i]")) {
    add("XChildBirth[i]", value("ZChildBirth[i]"))
} else {
    add("XChildBirth[i]", value("CA005[i]"))
}
```

XChildEdu Child's highest degree

```
if (!empty("ZChildEdu[i]")) {
    add("XChildEdu[i]", value("ZChildEdu[i]"))
} else {
    add("XChildEdu[i]", value("CA007[i]"))
}
```

XChildCoupleDis If the child has a spouse, show "[Child's name] and his/her spouse"

```
if (equal("CA010[i]", "1") || equal("CA010[i]", "2")) {
    add("XChildCoupleDis[i]", value("XChildPanAliveName[i]")+"and his/her spouse")
} else if (equal("CA010[i]", "3") || equal("CA010[i]", "4") || equal("CA010[i]", "5") || equal("CA010[i]",
↪  "6") || equal("CA010[i]", "997") || equal("CA010[i]", "999")) {
    add("XChildCoupleDis[i]", value("XChildPanAliveName[i]"))
}
```

XIntroCB002 If the respondent has no child alive when interviewed, show INTRO before CB002

```
if (greater("XChildAliveNum", "0")) {
    add("XIntroCB002", "")
} else {
    add("XIntroCB002", "We would like to know some information on household members in addition to
    ↪  "+pre("XFamilyRAndS")+"; a household member is someone who lives together with
    ↪  "+pre("XFamilyRAndS")+" and with whom "+pre("XFamilyRAndS")+" would share family income and
    ↪  expenses")
}
```

XHHOtherDis If need to ask whether the child is a household member, show "In addition to the selected household members, "

```
if (equal("XChildAliveNum", "0")) {
    add("XHHOtherDis", "")
} else if (greater("XChildAliveNum", "0")) {
```

```
    add("XHHOtherDis", "In addition to the selected household members, ")
}
```

XHHMemberNum Number of household members, minimum 0, maximum 25

```
for (var i1 = 1; i1 <=25 ; i1++) {
    if (selected("CB001", i1)) {
        add("XHHMemberNum", value("XHHMemberNum")+1)
        add("XHHMemberName["+value("XHHMemberNum")+"]", value("XChildAliveName["+i1+"]"))
        add("XHHMemberAge["+value("XHHMemberNum")+"]", 2020-value("XChildBirth["+i1+"]"))
    }
}
for (var i1 = 1; i1 <=10 ; i1++) {
    if (selected("CB002", i1)) {
        add("XHHMemberNum", value("XHHMemberNum")+1)
        add("XHHMemberName["+value("XHHMemberNum")+"]", value("CB002_1[i1]"))
    }
}
```

XHHMemberName Household member's name

```
see above
```

XHHMemberAge Household member's age

```
for (var i1 = 1; i1 <=25 ; i1++) {
    if (selected("CB001", i1)) {
        add("XHHMemberAge["+value("XHHMemberNum")+"]", 2020-value("XChildBirth["+i1+"]"))
    }
}
if (!equal("CB004[i]", "-1")) {
    add("XHHOtherAgeIter", value("XHHOtherAgeIter")+1)
    add("XHHMemberAge["+value("XHHOtherAgeIter")+"]", value("CB004[i]"))
} else {
    add("XHHOtherAgeIter", value("XHHOtherAgeIter")+1)
    add("XHHMemberAge["+value("XHHOtherAgeIter")+"]", "")
}
```

XHHOtherMemberNum Number of other household members, maximum 10

```
add("XHHOtherMemberNum", "0")
for (var i1 = 1; i1 <=10 ; i1++) {
    if (selected("CB002", i1)) {
        add("XHHOtherMemberNum", value("XHHOtherMemberNum")+1)
        add("XHHOtherMemberName["+value("XHHOtherMemberNum")+"]", value("CB002_1[i1]"))
    }
}
```

XHHOtherMemberName Other household member's name

```
see above
```

XHHOtherAgeIter Intermediate variable for looping age calculation for household member

```
if (equal("XChildAliveNum", "0")) {
    add("XHHOtherAgeIter", "0")
} else if (selected("CB001", 99)) {
    add("XHHOtherAgeIter", "0")
} else {
    add("XHHOtherAgeIter", count("CB001"))
}
```

XAffectReunion Wether family Chinese New Year reunion was affected by COVID-19

```
if (equal("CC001", "1") || equal("CC002", "1")) {
    add("XAffectReunion", "1")
} else {
    add("XAffectReunion", "0")
}
```

XCouLiveList Generate name list according to whether the household respondent lives with their spouse

```
if (empty("XFamilyS")) {
    add("XCouLiveList[1]", value("XFamilyR"))
} else if (!empty("XFamilyS") && equal("CC005", "2")) {
    add("XCouLiveList[1]", value("XFamilyR"))
    add("XCouLiveList[2]", value("XFamilyS"))
} else {
    add("XCouLiveList[1]", value("XFamilyRAndS"))
}
```

XHHOtherMemPreload Generate the list for other household members starting from i=26

```
for (var k = 1; k <= 10; k++) {
    add("XHHOtherMemPreload["+(k+25)+"]", value("XHHOtherMemberName[k]"))
}
```

ZChildName Name of respondent's child

D Health Status and Functioning

D.Proxy Mode Confirmation

proxy_5 Interviewer record: Enter proxy mode?

1. Yes
2. No

DA.Health Status (1)

【**INTRO**: Next, I have some questions about [XRName]'s health.】

DA001 Would you say your health is very good, good, fair, poor or very poor?

【**IWER**: Interviewer should read all the following options】

1. Very good
2. Good
3. Fair
4. Poor
5. Very poor

997. Don't know

DA002[i] Compared to [ZIWTime], is [XRName]'s [XChroDisType[i]] better, about the same, or worse?

1. Better
2. Worse
3. About the same

99. Did't have the disease in LAST IW

DA002_1[i] In [ZIWTime], [XRName] was known to have [XChroDisType[i]]. Compared to [ZIWTime], is [XRName]'s [XChroDisType[i]] better, about the same, or worse?

1. Better
2. Worse
3. About the same

99. Did't have the disease in LAST IW

DA003[i] Have [XRName] been diagnosed with [XChroDisType[i]] by a doctor?

1. Yes
2. No

DA004[i] Do you know if [XRName] have [XChroDisType[i]]?

1. Yes, I know R have this disease
2. No, I don't R have this disease
3. I Don't know if R have this disease

DA005 In the last month, have [XRName] visited a public hospital, private hospital, public health center, clinic, or health worker's or doctor's practice, or been visited by a health worker or doctor for outpatient care? (Not including physical examination)

1. Yes
2. No

DA006 How many times did [XRName] visit/been visited during the last month? ______ [hc((0, 99), int, ∅), sc([1, 20), int, ∅)] Times

DA007 Have [XRName] received inpatient care in the past year?

【**IWER**: past year refers to the past one year counted from today】

1. Yes
2. No

DA008 How many times have [XRName] received inpatient care during the past year? ______ [hc((0, 99), int, ∅), sc([1, 20), int, ∅)] Times

【**IWER**: Don't count inpatient care not received in the past year. Past year means within a year counted down from today】

DA009 During the outbreak of the COVID-19 pandemic period, was there any time when [XRName] needed medical (including dental) care, but delayed getting it, or did not get it at all because of the impact of the pandemic?

1. Yes
2. No

DA010 Please Specify what type(s) of care of health service did [XRName] delay or did not get during the pandemic? (Select All That Apply)

【**IWER**: Please read out all options and tick all that apply】

1. Major surgery requring hospitalization
2. Minor Surgery as an outpatient or day case
3. Seeing outpatient doctor
4. Getting prescription medications
5. Dentalcare
6. Other, Please specify ______ (**DA010_1**)

DA011 Why did [XRName] want to see the outpatient doctor during the pademic?Is it beacause of the newly emerged symptoms or diseases, the treatment of existing diseases, or the regular pysical screening examinations? (Select All That Apply)

1. Newly emerged symptoms or diseases
2. Treat existing diseases
3. Regular physical screening examinations

DA012 Why did [XRName] delay or not get that care? (Select All That Supply, Do Not Read Out the Options)

1. Couldn't get an appointment or the hospital cancelled regular appointment
2. The hospital resheduled regular arrangements
3. I decided it could wait
4. I was afraid to got to hospital
5. Other, please specify ______ (**DA012_1**)

DA013 Since [ZIWTime], when did [XRName] take the last physical examination? (Not include CAHRLS physical examination)

【**IWER**: Mark the year using four digits.Take down the month as its acutal number. For example, write January as "1" not "01", December as "12".

If do not remember month, enter “-1”】

1. ______ [hc([1900, 2020], *int*, ∅), sc([1920, 2020], *int*, ∅)] (**DA013_1**) Year ______ [hc([1, 12], *int*, −1)] (**DA013_2**) Month
2. Have never take any other physical examination since last survey

DA014 When did [XRName] take the last physical examination? (Not inluding the examination needed by a doctor in hospital when seeking medical care)

【**IWER**: Mark the year using four digits.Take down the month as its acutal number. For example, write January as “1” not “01”, December as “12”.
If do not remember month, enter “-1”】

1. ______ [hc([1900, 2020], *int*, ∅), sc([1920, 2020], *int*, ∅)] (**DA014_1**) Year ______ [hc([1, 12], *int*, −1)] (**DA014_2**) Month
2. Had never take physical examination yet

DA.Health Status (2)

DA019 Since [ZIWTime], Have [XRName] ever been in a traffic accident or any other kind of major accidental injury and received medical treatment?

1. Yes
2. No

DA020 Have [XRName] ever been in a traffic accident or any other kind of major accidental injury and received medical treatment?

1. Yes
2. No

DA021 Does injury caused by the accident limit [XRName]’s daily activities?

1. Yes
2. No

DA022 Since [ZIWTime],have [XRName] fallen down?

1. Yes
2. No

DA023 Have [XRName] fallen down?

1. Yes
2. No

DA024 How many times have the fallen down seriously enough to need medical treatment?
______ [hc([0, 99], *int*, ∅), sc([0, 20), *int*, ∅)] Times

DA025 Since [ZIWTime], have [XRName] ever fractured the hip?

【**IWER**: Hipbone refers to the bone besides the human waist, which cnsists of the left part and right part. The bone is divided into the ilium, ischial and pubic bones, and cartilage connections in childhood. In adulthood, the cartilage betweeen ossifies and becomes a whole, that is hipbon】

1. Yes
2. No

DA026 Have [XRName] ever fractured the hip?

【**IWER**: Hipbone refers to the bone besides the human waist, which cnsists of the left part and right part. The bone is divided into the ilium, ischial and pubic bones, and cartilage connections in childhood. In adulthood, the cartilage betweeen ossifies and becomes a whole, that is hipbone】

1. Yes
2. No

DA027 Are [XRName] often troubled with any body pains? Is if none, a little, somewhat, quite a bit, or very?

【**IWER**: The pain in all parts of R's body】

1. None
2. A little
3. Somewhat
4. Quite a bit
5. Very

DA028 On what part of your body do you feel pain? Please list all parts of body you are currently feeling pain.

1. Head (Headache)
2. Shoulder
3. Arm
4. Wrist
5. Fingers
6. Chest
7. Stomach (Stomachache)
8. Back
9. Waist
10. Buttocks
11. Leg
12. Knees
13. Ankle
14. Toes
15. Neck
16. Other, please specify ______ (**DA028_1**)

DA029 Next I'll ask a question about your subjective life expectancy, this question reflect your anticipation about your own health status. Suppose there are 5 steps, where the lowest step represents the smallest chance and the highest step represents the highest chance, on what step do you think is your chance in reaching the age of [XFAgePossibility]? Is it almost impossible, not very likely, maybe, very likely, or almost certain?

1. Almost impossible
2. Not very likely
3. Maybe
4. Very likely
5. Almost certain

997. Don't Know

DA.Health Status (3)

DA030 During the past month, how many hours of actual sleep did [XRName] get at night (average hours for one night)? (This may be shorter than the number of hours [XRName] spend in bed.) ______ [$\mathtt{hc}([0,24], real, -1)$] Hours

【**IWER**: Please enter "-1" if R cannot answer】

DA031 During the past month, how long did [XRName] take a nap after lunch? ______ [$\mathtt{hc}([0,300], int, -1)$] Minutes

【**IWER**: If R didn't take a nap, please record for 0. Please enter "-1" if R cannot answer】

【**INTRO**: Now we would like to ask about the amount of time [XRName] spend on different types of physical activities in a usual week.】

DA032[i] Now, think about all the [XPsyActType[i]] that [XRName] do in a usual week.Think only about those physical activities that [XRName] did for at least 10 minutes at a time. During a usual week,did [XRName] do any [XPsyActType[i]] for at least 10 minutes continuously?

1. Yes
2. No

DA033[i] During a usual week, on how many days did [XRName] do [XPsyActType[i]] for at least 10 minutes? ______ [$\mathtt{hc}([1,7], int, \varnothing)$] Days

DA034[i] How much time did [XRName] usually spend doing [XPsyActType[i]] on one of those days?

1. <2 hours
2. >=2 hours

DA035[i] How much time did [XRName] usually spend doing [XPsyActType[i]] on one of those days?

1. <30 hours
2. >=30 hours

DA036[i] How much time did [XRName] usually spend doing [XPsyActType[i]] on one of those days?

1. <4 hours
2. >=4 hours

DA037[i] What's the purpose for doing [XPsyActType[i]], for entertainment, job demand or exercise in doing these physical activities?

1. Job demands
2. Entertainments
3. Exercise
4. Other, please specify ______ (**DA037_1[i]**)

DA038 Have [XRName] done any of these activities in the last month? (Select All That Apply)

1. Interacted with friends
2. Played Ma-jong, played chess, played cards, or went to community club
3. Provided help to family, friends, or neighbors who do not live with you

4. Went to a sport, social, or other kind of club
5. Took part in a community-related organization
6. Done voluntary or charity work, of cared for a sick or disabled adult who does not live with you
7. Attended an educational or training course
8. Other, please specify ______ (**DA038_1**)
9. None of the above

[conflict(9, $[9]^c$)]

DA039[i] How often in the last month did [XRName] do these activities [XSocType[i]] ? Almost daily, almost every week, or not regularly?

1. Almost daily
2. Almost every week
3. Not regularly

DA040 Have [XRName] used the internet in the past month? including chat, watch news, watch videos,play games, financial management and others

1. Yes
2. No

DA041 Which types of devices does [XRName] use to access the Internet? (Select All That Apply)

1. Desktop computer
2. Laptop computer
3. Tablet computer (such as IPAD)
4. Cellphone
5. Other devices, please specify ______ (**DA041_1**)

DA042 What does [XRName] usually do on the Internet?(Select All That Apply)

1. Chat
2. Watch news
3. Watch videos
4. Play games
5. Financial management
6. Others, please specify ______ (**DA042_1**)

DA043 Does [XRName] use mobile payments, such as Alipay and WeChat pay?

1. Yes
2. No

DA044 Does [XRName] use WeChat?

1. Yes
2. No

DA045 Do [XRName] post WeChat moments?

1. Yes
2. No

DA046 Have [XRName] ever chewed tobacco, smoked a pipe, smoked self-rolled cigarettes, or

smoked cigarettes/cigars?
1. Yes
2. No

DA047 Does [XRName] still have the habit or have totally quit?
1. Still have
2. Quit
3. Never smoked

DA048 Which products did/does [XRName] normally use?
1. Smoking a pipe
2. Smoking self-rolled cigarettes
3. Filtered cigarette
4. Unfiltered cigarette
5. Cigar
6. Water cigarettes

DA049 At what age did [XRName] totally quit smoking?
【**IWER**: Please enter "-1" if R cannot answer】
1. Age ______ [hc$([0,120],int,-1)$, sc$((0,100],int,-1)$] (**DA049_1**) Age
2. Year ______ [hc$([1900,2020],int,-1)$, sc$([1920,2020],int,-1)$] (**DA049_2**) Year

DA050_1 In one day about how many cigarettes does [XRName] consume now? ______ [hc$([0,200],int,\varnothing)$] Cigarettes

DA050_2 In one day about how many cigarettes did [XRName] consume before totally quitting? ______ [hc$([0,200],int,-1)$] Cigarettes
【**IWER**: Please enter "-1" if R cannot answer】

DA051 Did [XRName] drink any alcoholic beverages, such as beer, wine, or liquor in the past year? How often?
1. Drink more than once a month
2. Drink but less than once a month
3. None of these

DA052 How often did [XRName] drink per month in the last year?
1. Once a month
2. 2-3 times a month
3. Once a week
4. 2-3 times a week
5. 4-6 times a week
6. Once a day
7. Twice a day
8. More than twice a day

DB. Functional Limitations and Helpers (1)

【**INTRO**: Next we want to know about [XRName]'s daily activities.Please tell me whether [XRName] have difficulties performing any of the following tasks because of physical, mental, emotional or memory problems.Exclude any difficulties that are expected to last less than three months】

DB001 Because of health and memory problems, does [XRName] have any difficulty with dressing? Dressing includes taking clothes out from a closet, putting them on, buttoning up, and fastening a belt.

1. Don't have any difficulty
2. Have difficulty but can still do it
3. Have difficulty and need help
4. Can not do it

DB002 Does anyone ever help [XRName] dress?

1. Yes
2. No

DB003 Because of health and memory problems, does [XRName] have any difficulty with bathing or showering?

1. Don't have any difficulty
2. Have difficulty but can still do it
3. Have difficulty and need help
4. Can not do it

DB004 Does anyone ever help [XRName] bathe?

1. Yes
2. No

DB005 Because of health and memory problems, does [XRName] have any difficulty with eating, such as cutting up your food? (Definition: By eating, we mean eating food by oneself when it is ready)

1. Don't have any difficulty
2. Have difficulty but can still do it
3. Have difficulty and need help
4. Can not do it

DB006 Does anyone ever help [XRName] eat?

1. Yes
2. No

DB007 Does [XRName] have any difficulty with getting into or out of bed?

1. Don't have any difficulty
2. Have difficulty but can still do it
3. Have difficulty and need help
4. Can not do it

DB008 Does anyone ever help [XRName] get in or out of bed?

1. Yes
2. No

DB009 Because of health and memory problems, does [XRName] have any difficulties with using the toilet, including getting up and down?

1. Don't have any difficulty
2. Have difficulty but can still do it
3. Have difficulty and need help
4. Can not do it

DB010 Does anyone ever help [XRName] use the toilet?

1. Yes
2. No

DB011 Because of health and memory problems, does [XRName] have any difficulties with controlling urination and defecation? If use a catheter (conduit) or a pouch by self, then [XRName] are not considered to have difficulties.

1. Don't have any difficulty
2. Have difficulty but can still do it
3. Have difficulty and need help
4. Can not do it

DB012 Because of health and memory problems, does [XRName] have any difficulties with doing household chores? (Definition: By doing household chores, we mean house cleaning, doing dishes, making the bed, and arranging the house)

【**IWER**: If R cannot mop the floor, but can scrub, or R cannot fold heavy bedding, but is able to do light ones, then mark (3)】

1. Don't have any difficulty
2. Have difficulty but can still do it
3. Have difficulty and need help
4. Can not do it

DB013 Does anyone help [XRName] do household chores?

1. Yes
2. No

DB014 Because of health and memory problems, does [XRName] have any difficulties with preparing hot meals? (Definition: By preparing hot meals, we mean preparing ingredients, cooking, and serving food)

【**IWER**: If another person prepares ingredients or if R can cook rice, but is not able to prepare side dishes, then mark (3) 】

1. Don't have any difficulty
2. Have difficulty but can still do it
3. Have difficulty and need help
4. Can not do it

DB015 Does anyone help [XRName] prepare hot meals?

1. Yes
2. No

DB016 Because of health and memory problems, does [XRName] have any difficulties with

shopping for groceries? By shopping, we mean deciding what to buy and paying for it.

1. Don't have any difficulty
2. Have difficulty but can still do it
3. Have difficulty and need help
4. Can not do it

DB017 Does anyone help [XRName] shop for groceries?

1. Yes
2. No

DB018 Because of health and memory problems, does [XRName] have any difficulties with making phone calls?

1. Don't have any difficulty
2. Have difficulty but can still do it
3. Have difficulty and need help
4. Can not do it

DB019 Does anyone help [XRName] make telephone calls?

1. Yes
2. No

DB020 Because of health and memory problems, does [XRName] have any difficulties with taking medications? By taking medications, we mean taking the right portion of medication right on time.

1. Don't have any difficulty
2. Have difficulty but can still do it
3. Have difficulty and need help
4. Can not do it

DB021 Does anyone help [XRName] take medications?

1. Yes
2. No

DB022 Because of health and memory problems, does [XRName] have any difficulties with managing your money, such as paying your bills, keeping track of expenses, or managing assets?

1. Don't have any difficulty
2. Have difficulty but can still do it
3. Have difficulty and need help
4. Can not do it

DB023 Does anyone help [XRName] manage money?

1. Yes
2. No

DB. Functional Limitations and Helpers (2)

DB024 Who most often helps [XRName] with (dressing, bathing, eating, getting out of bed, using the toilet, controlling urination and defecation, doing chores, preparing hot meals, shopping, managing money, making phone calls, taking medications)? (Select All That Apply)

1. Spouse
2. Father, Mother, Father-in-law, Mother-in-law
3. Children, Children's spouses, Grandson, Granddaughter
4. Sibling, Brother-in-law, Sister-in-law, Sibling of spouse, Children of sibling, Brother-in-law of spouse, Sister-in-law of spouse, Children of brother-in-law, Children of sister-in-law
5. Other relative
6. Paid helper (such as nanny), in total ______ [hc$((0, 99), int, -1)$, sc$([1, 10), int, -1)$] (**DB024_1**) persons
7. Volunteer
8. Employee(s) of facility
9. Employee(s) of homed-based elderly care institutions
10. Community
11. Other, please specify ______ (**DB024_2**)

DB025 Father, mother, father-in-law, mother-in-law, Who help [XRName] most? (Select All That Apply)

1. Father
2. Mother
3. Father-in-law
4. Mother-in-law

DB026 For the children, children-in-law, grandson, granddaughter who helped [XRName], which children's family are they from? (Select All That Apply)

1-25. [XChildPanAliveName[i]]
26-35. Other child, name ______ (**DB026_1[i]**)

DB027[i] For the family members of [XHelperChild[i]], who helps [XRName] in person? (Select All That Apply)

1. [XHelperChild[i]] himself/herself
2. [XHelperChild[i]] his/her spouse
3. [XHelperChild[i]] his/her children.How many helped [XRName] in person? ______ [hc$((0, 99), int, -1)$, sc$([1, 10), int, -1)$] (**DB027_1[i]**)

DB028 For the siblings, spouse and children of siblings, siblings of your spouse, spouse and children of siblings of your spouse who helped [XRName], which siblings' family are they from? (Select All That Apply)

1-30. [XSibName[i]]
31-40. Other sibling, name ______ (**DB028_1[i]**)

DB029[i] For the family members of [XHelperSib[i]], who help [XRName] in person? (Select All

That Apply)

1. [XHelperSib[i]] himself/herself
2. [XHelperSib[i]] his/her spouse
3. [XHelperSib[i]] his/her children. how many helped [XRName] in person? ______ [hc$((0,99), int, -1)$, sc$([1,10), int, -1)$] **(DB029_1[i])**

DB030 The number of the relatives who helped [XRName] in person ______ [hc$((0,99), int, -1)$, sc$([1,10), int, -1)$]

【**IWER**: Please enter "-1" if R cannot answer】

DB030_1 What's their relationship with [XRName]? ______

DB031 The number of the others who helped [XRName] in person ______ [hc$((0,99), int, -1)$, sc$([1,10), int, -1)$]

【**IWER**: Please enter "-1" if R cannot answer】

DB031_1 What's that their relationship with [XRName]? ______

DB032 From all the helpers list below, please select the most important 7 helpers for [XRName].

1-99. [XHelper[i]]

DB033[i] During the last month, on about how many days did [XHelpList[i]] help [XRName]? ______ [hc$([0,31], int, -1)$] Days

【**IWER**: Please enter "-1" if R cannot answer】

DB034[i] On the days [XHelpList[i]] helps [XRName], about how many hours per day is that? ______ [hc$([0,24], int, -1)$] Hours

【**IWER**: less than an hour, mark 1;
Please enter "-1" if R cannot answer.】

DB035[i] Is [XHelpList[i]] living in [XRName]'s home when provinding help?

1. Yes
2. No

DB048 Because of the impact of epidemic, was there any changes in care received by [XRName]? Was the care received become more, become less or not changed?

1. Become more
2. Become less
3. Not changed

DB. Functional Limitations and Helpers (3)

DB036 Suppose that in the future, [XRName] needed help with basic daily activities like eating or dressing. Do [XRName] have relatives or friends who would be willing and able to help over a long period of time?

1. Yes
2. No

DB037 What is the relationship to [XRName] of that person or those persons? (Select All That Apply)

1. Spouse
2. Father, Mother, Father-in-law, Mother-in-law
3. Children, Children's spouses, Grandson, Granddaughter
4. Sibling, Brother-in-law, Sister-in-law, Sibling of spouse, Children of sibling, Brother-in-law of spouse, Sister-in-law of spouse, Children of brother-in-law, Children of sister-in-law
5. Other relative
6. Paid helper (such as nanny), in total ______ [hc$((0,99), int, -1)$, sc$([1,10), int, -1)$] (**DB037_1**) persons
7. Volunteer
8. Employee(s) of facility
9. Employee(s) of home-based elderly care institutions
10. Community
11. Other, please specify ______ (**DB037_2**)

DB038 Father, mother, father-in-law, mother-in-law, who will help [XRName] in future? (Select All That Apply)

1. Father
2. Mother
3. Father-in-law
4. Mother-in-law

DB039 For the children, children-in-law, grandchildren who will help [XRName] in future, which children's family are they from? (Select All That Apply)

1-25. [XChildPanAliveName[i]]
26-35. Other child, name ______ (**DB039_1[i]**)

DB040 For the siblings, spouse and children of siblings, spouse's siblings, spouse and children of spouse's siblings who will help [XRName] in future, which children's family are they from? （Select All That Apply）

1-30. [XSibName[i]]
31-40. Other sibling, name ______ (**DB040_1[i]**)

DB041 The number of other relatives who will help [XRName] in person in future ______ [hc$((0,99), int, -1)$, sc$([1,10), int, -1)$]

【**IWER**: Please enter "-1" if R cannot answer】

DB042 The number of others who will help [XRName] in person in future ______ [hc$((0,99), int, -1)$, sc$([1,10), int, -1)$]

【**IWER**: Please enter "-1" if R cannot answer】

【**INTRO**: We would like to know whether [XRName]'s health problems limit the ability to work】

DB043 Do you think the following description fits [XRName]: I cannot work because of my disability or health problems.

1. I am unable to work

2. I cannot work long hours
3. I don't have any problem

DB044 Do you think the following description fits [XRName]: I cannot do housework because of my disability or health problems.

1. I am unable to do housework
2. I cannot do housework for an extended period of time
3. I don't have any problem

DB045 Interviewer Observation: How often did [XRName] receive assistance in answering this section?

【**IWER**: If it is answered by a proxy, please record the R's reaction】

1. Never
2. A few times
3. Most or all of the time
4. The section was completed by a proxy respondent（R is absent）

DB046 Interviewer Observation: What is the proxy respondent's relationship to [XRName]?

【**IWER**: What is the proxy respondent's relationship to R? If unknown, please ask the proxy】

1. Spouse
2. Mother
3. Father
4. Mother-in-law
5. Father-in-law
6. Sibling
7. Brother-in-law, sister-in-law
8. Child
9. Spouse of child
10. Grandchild
11. Other relative
12. Helper or other non-relative

DB047 Interviewer Observation: What is the main reason for proxy ([XRName] is absent)?

【**IWER**: Please record the reason for proxy】

1. R has serious physical handicaps
2. R has serious mental handicaps
3. R has declined this interview
4. Other, please specify ______ (**DB047_1**)

DC.Cognition and Depression (1)

【**INTRO**: First I would like to ask you some questions to check your memory and concentration. Some of them may be easy and some may be hard.】

DC001 What is the year?

1. Correct
2. Error

997. Don’t Know
999. Refuse

DC002 What is the season of the year?

1. Correct
2. Error
997. Don’t Know
999. Refuse

DC003 What is the date?

【**IWER**: Lunar date is also correct. R is not allowed to check mobile phone or calendars】

1. Correct
2. Error
997. Don’t Know
999. Refuse

DC004 What is the day of the week?

1. Correct
2. Error
997. Don’t Know
999. Refuse

DC005 What is the month?

【**IWER**: Lunar date is also correct. R is not allowed to check mobile phone or calendars】

1. Correct
2. Error
997. Don’t Know
999. Refuse

DC006 How would you rate your memory at the present time? Would you say it is excellent, very good, good, fair or poor?

1. Excellent
2. Very Good
3. Good
4. Fair
5. Poor
997. Don’t Know

【**INTRO**: Next, I need you to listen carefully, and do as I say. Could you listen to me carefully now? Are you ready? Let's start.
please calculate 100 minus 7, and keep minus 7 continuously, tell me each answer you get from minus 7, until I say stop.】

DC007_1 Record answer from R:

【**IWER**: After read the introduction, do not give any others instructions during the test, and do not remind the subject what should be done. No extra instructions except saying ``continue'.
When repondent don't give a positive response, and R still don't know what to do after been reminded 3 times to continue, IWER can choose the Don't Know option.

This test allows R to use paper and pen as assistant.】

1. Record Answer ______ [] (**DC007_1_1**)
997. Don't Know
999. Refuse

DC007_2 Record answer from R:

1. Record Answer ______ [] (**DC007_2_1**)
997. Don't Know
999. Refuse

DC007_3 Record answer from R:

1. Record Answer ______ [] (**DC007_3_1**)
997. Don't Know
999. Refuse

DC007_4 Record answer from R:

1. Record Answer ______ [] (**DC007_4_1**)
997. Don't Know
999. Refuse

DC007_5 Record answer from R:

1. Record Answer ______ [] (**DC007_5_1**)
997. Don't Know
999. Refuse

DC008 Interviewer Observation: Please Indicate whether the R used paper and pencil or any other aid when completing the number subtraction?

1. Used aid
2. Did not use aid

DC009 Here is a drawing. Please copy the drawing on this paper. `transferPic("DC009")`
Please click on the blank box below, and take photo of the draw by R ______ (**DC009_photo**)

【**IWER**: Score correct if A) There are two five-sided figures which intersect to form a four-sided figure and B) All angles in the five sided figure much be preserved】

1. Correct
2. Error
3. Not assessed (Unable to complete due to physical reasons)
997. Don't Know
999. Refuse

DC.Cognition and Depression (2)

DC010_1 I am going to read a list of 10 words. Read each word out loud as I read it to you. When finishing reading, I will ask you to recall all ten words. Is this clear? `transferPic("XWordlist","9 3 1 2 10 4 5 6 8 7")`

【**IWER**: Probe if R understands this task.
Read wordlist at a slow, steady rate, approximately one word every two seconds.Ask R to repeat the word after you read it out.
Show the wordlist only when R had impaired hearing. Read the words out loud as you show the wordlist.R should repeat the word after you read it our loud, before moving to the next word.】

1. Yes
2. No

DC010_2 I am going to read a list of 10 words. Read each word out loud as I read it to you. When finishing reading, I will ask you to recall all ten words. Is this clear? `transferPic("XWordlist","9 3 1 2 10 4 5 6 8 7")`
【**IWER**:Probe if R understands this task.
Read wordlist at a slow, steady rate, approximately one word every two seconds.Ask R to repeat the word after you read it out.
Show the wordlist only when R had impaired hearing. Read the words out loud as you show the wordlist.R should repeat the word after you read it our loud, before moving to the next word.】

1. Yes
2. No

DC010_3 I am going to read a list of 10 words. Read each word out loud as I read it to you. When finishing reading, I will ask you to recall all ten words. Is this clear? `transferPic("XWordlist","9 3 1 2 10 4 5 6 8 7")`
【**IWER**:Probe if R understands this task.
Read wordlist at a slow, steady rate, approximately one word every two seconds.Ask R to repeat the word after you read it out.
Show the wordlist only when R had impaired hearing. Read the words out loud as you show the wordlist.R should repeat the word after you read it our loud, before moving to the next word.】

1. Yes
2. No

DC011 Interviewer Observation: Please record reasons for not able to complete this test? (Select All That Apply)

1. Refuse or unwilling to to
2. Cannot speak in whole life
3. Cannot speak when getting old
4. Deaf or poor hearing
5. Other, please specify ______ (**DC011_1**)

DC012 Now please tell me the words you can recall.
【**IWER**: Read wordlist at a slow, steady rate, approximately one word every two seconds.Ask R to repeat the word after you read it out.
Show the wordlist only when R had impaired hearing. Read the words out loud as you show the wordlist.R should repeat the word after you read it our loud, before moving to the next word.
PERMIT as much time as R wishes -- up to about 2 minutes】

1. [XWordlist[9]]
2. [XWordlist[3]]
3. [XWordlist[1]]

4. [XWordlist[2]]
5. [XWordlist[10]]
6. [XWordlist[4]]
7. [XWordlist[5]]
8. [XWordlist[6]]
9. [XWordlist[8]]
10. [XWordlist[7]]
11. None word being recalled
12. Refuse to recall
13. Can not understand or unable to do

[conflict(11, 12, 13, $[11, 12, 13]^c$)]

【**INTRO**: I am going to read the same list of words in a different order. Repeat each word out loud after I read it to you. Later I will ask you to recall all ten words. Are you ready?】

DC013 Now please tell me the words you can recall. transferPic("XWordlist","5 2 9 10 3 1 8 4 6 7")

【**IWER**: Read wordlist at a slow, steady rate, approximately one word every two seconds.Ask R to repeat the word after you read it out.
Show the wordlist only when R had impaired hearing. Read the words out loud as you show the wordlist.R should repeat the word after you read it our loud, before moving to the next word.
PERMIT as much time as R wishes -- up to about 2 minutes】

1. [XWordlist[5]]
2. [XWordlist[2]]
3. [XWordlist[9]]
4. [XWordlist[10]]
5. [XWordlist[3]]
6. [XWordlist[1]]
7. [XWordlist[8]]
8. [XWordlist[4]]
9. [XWordlist[6]]
10. [XWordlist[7]]
11. None word being recalled
12. Refuse to recall

[conflict(11, 12, $[11, 12]^c$)]

【**INTRO**: I am going to read the same list of words in a different order. Repeat each word out loud after I read it to you. Later I will ask you to recall all ten words. Are you ready?】

DC014 Now please tell me the words you can recall. transferPic("XWordlist","1 2 3 4 5 6 7 8 9 10")

【**IWER**: Read wordlist at a slow, steady rate, approximately one word every two seconds.Ask R to repeat the word after you read it out.
Show the wordlist only when R had impaired hearing. Read the words out loud as you show the wordlist.R should repeat the word after you read it our loud, before moving to the next word.
PERMIT as much time as R wishes -- up to about 2 minutes】

1. [XWordlist[1]]
2. [XWordlist[2]]
3. [XWordlist[3]]

4. [XWordlist[4]]
5. [XWordlist[5]]
6. [XWordlist[6]]
7. [XWordlist[7]]
8. [XWordlist[8]]
9. [XWordlist[9]]
10. [XWordlist[10]]
11. None word being recalled
12. Refuse to recall

[conflict(11, 12, $[11, 12]^c$)]

DC015 Interviewer Observation: Indicate whether any of the following apply to the administation of the word lists (Select All That Aplly)

1. An interrution occured during administration of list
2. Other situations ______ (**DC015_1**)
3. No issues occured

[conflict(3, $[3]^c$)]

【**INTRO**: The 10 items below refer to how you have felt and behaved during the last week. Every item has the same selective answers including rarely or none of the time, some, occasionally, and most or all of the time. Choose the appropriate response】

DC016 I was bothered by things that don't usually bother me.

【**IWER**: If R can not understand this question,please read the question again,don't select Don't Know without try】

1. Rarely or none of the time (<1 day)
2. Some or a little of the time (1-2 days)
3. Occasionally or a moderate amount of the time (3-4 days)
4. Most or all of the time (5-7 days)
997. Don't Know
999. Refuse

DC017 I had trouble keeping my mind on what I was doing.

【**IWER**: If R can not understand this question,please read the question again,don't select Don't Know without try】

1. Rarely or none of the time (<1 day)
2. Some or a little of the time (1-2 days)
3. Occasionally or a moderate amount of the time (3-4 days)
4. Most or all of the time (5-7 days)
997. Don't Know
999. Refuse

DC018 I felt depressed.

【**IWER**: If R can not understand this question,please read the question again,don't select Don't Know without try】

1. Rarely or none of the time (<1 day)
2. Some or a little of the time (1-2 days)

3. Occasionally or a moderate amount of the time (3-4 days)
4. Most or all of the time (5-7 days)
997. Don’t Know
999. Refuse

DC019 I felt everything I did was an effort.

【**IWER**: If R can not understand this question,please read the question again,don't select Don't Know without try】

1. Rarely or none of the time (<1 day)
2. Some or a little of the time (1-2 days)
3. Occasionally or a moderate amount of the time (3-4 days)
4. Most or all of the time (5-7 days)
997. Don’t Know
999. Refuse

DC020 I felt hopeful about the future.

【**IWER**: If R can not understand this question,please read the question again,don't select Don't Know without try】

1. Rarely or none of the time (<1 day)
2. Some or a little of the time (1-2 days)
3. Occasionally or a moderate amount of the time (3-4 days)
4. Most or all of the time (5-7 days)
997. Don’t Know
999. Refuse

DC021 I felt fearful.

【**IWER**: If R can not understand this question,please read the question again,don't select Don't Know without try】

1. Rarely or none of the time (<1 day)
2. Some or a little of the time (1-2 days)
3. Occasionally or a moderate amount of the time (3-4 days)
4. Most or all of the time (5-7 days)
997. Don’t Know
999. Refuse

DC022 My sleep was restless.

【**IWER**: If R can not understand this question,please read the question again,don't select Don't Know without try】

1. Rarely or none of the time (<1 day)
2. Some or a little of the time (1-2 days)
3. Occasionally or a moderate amount of the time (3-4 days)
4. Most or all of the time (5-7 days)
997. Don’t Know
999. Refuse

DC023 I was happy.

【**IWER**: If R can not understand this question,please read the question again,don't select Don't Know

without try】

1. Rarely or none of the time (<1 day)
2. Some or a little of the time (1-2 days)
3. Occasionally or a moderate amount of the time (3-4 days)
4. Most or all of the time (5-7 days)

997. Don’t Know
999. Refuse

DC024 I felt lonely.

【**IWER**: If R can not understand this question,please read the question again,don't select Don't Know without try】

1. Rarely or none of the time (<1 day)
2. Some or a little of the time (1-2 days)
3. Occasionally or a moderate amount of the time (3-4 days)
4. Most or all of the time (5-7 days)

997. Don’t Know
999. Refuse

DC025 I could not get “going”.

【**IWER**: If R can not understand this question,please read the question again,don't select Don't Know without try】

1. Rarely or none of the time (<1 day)
2. Some or a little of the time (1-2 days)
3. Occasionally or a moderate amount of the time (3-4 days)
4. Most or all of the time (5-7 days)

997. Don’t Know
999. Refuse

DC026 Please think about your life-as-a-whole. How satisfied are you with it? Are you completely satisfied, very satisfied, somewhat satisfied, not very satisfied, or not at all satisfied?

1. Completely satisfied
2. Very satisfied
3. Somewhat satisfied
4. Not very satisfied
5. Not at all satisfied

DC027 How satisfied are you with your relationship with children? Are you completely satisfied, very satisfied, somewhat satisfied, not very satisfied, or not at all satisfied?

【**IWER**: Only for respndents who have living offspring】

1. Completely satisfied
2. Very satisfied
3. Somewhat satisfied
4. Not very satisfied
5. Not at all satisfied
6. No child now

【**INTRO**: A few minutes ago I asked you to learn a list of ten words which you read one at a time from cards. Now I want you to try to recall as many of those 10 words as you can. OK, now tell me as many of those ten words as you can remember.】

DC028 Please select words that is being correctly recalled.

【**IWER**: PERMIT as much time as R wishes -- up to about 2 minutes.】

1. [XWordlist[1]]
2. [XWordlist[2]]
3. [XWordlist[3]]
4. [XWordlist[4]]
5. [XWordlist[5]]
6. [XWordlist[6]]
7. [XWordlist[7]]
8. [XWordlist[8]]
9. [XWordlist[9]]
10. [XWordlist[10]]
11. None word being recalled
12. Refuse to recall

[conflict(11, 12, $[11, 12]^c$)]

DC029 Interviewer Observation: Does any of the following happen during the interview? (Select All That Apply)

1. Poor eyesight
2. Poor hearing withour hearing-aid
3. Wearing hearing-aid
4. Shaking hands
5. The interview is interrupted by some stuff or noises
6. The quality of the interview is doubted due to some emotional problems of R
7. Others, please specify ______ (**DC029_1**)
8. None

[conflict(8, $[8]^c$)]

DC030 Interviewer Observation: What kind of language is used by interviewer during the interview?

1. Mandarin
2. Local dialect
3. Other, please specify ______ (**DC030_1**)

Auxiliary Variable Definition

ZDisease R had Doctor Diagnosed Disease[i] at ZIWTime

ZSelfDisease R had Self-reported Disease[i] at ZIWTime

ZSmoke R had Ever Smoked at ZIWTime

ZSibName List of Siblings' Names of R at ZIWTime

ZSibNameS List of Siblings' Names of R's Spouse at ZIWTime

ZChildNum Number of R's Children at ZIWTime

XChroDisType Types of Chronic Disease

```
add("XChroDisType", ["Hypertension", "Dyslipidemia (elevation of low density lipoprotein, triglycerides
↪ (TGs),and total cholesterol, or a low high density lipoprotein level)", "Diabetes or high blood
↪ sugar", "Cancer or malignant tumor (excluding minor skin cancers)", "Chronic lung diseases, such as
↪ chronic bronchitis , emphysema ( excluding tumors, or cancer)", "Liver disease (except fatty liver,
↪ tumors, and cancer)", "Heart attack, coronary heart disease, angina, congestive heart failure, or
↪ other heart problems", "Stroke", "Kidney disease (except for tumor or cancer)", "Stomach or other
↪ digestive diseases (except for tumor or cancer)", "Emotional, nervous, or psychiatric problems",
↪ "Memory-related disease (dementia and brain atrophy )", "Parkinson's disease", "Arthritis or
↪ rheumatism", "Asthma (Not lung diseases)"])
```

XFAgePossibility Specific Age Expected

```
if (!greater("XRAge", "65") && !equal("XRAge", "65")) {
    add("XFAgePossibility", "75")
}
if ((greater("XRAge", "65") || equal("XRAge", "65")) && !greater("XRAge", "69")) {
    add("XFAgePossibility", "80")
}
if ((greater("XRAge", "70") || equal("XRAge", "70")) && !greater("XRAge", "74")) {
    add("XFAgePossibility", "85")
}
if ((greater("XRAge", "75") || equal("XRAge", "75")) && !greater("XRAge", "79")) {
    add("XFAgePossibility", "90")
}
if ((greater("XRAge", "80") || equal("XRAge", "80")) && !greater("XRAge", "84")) {
    add("XFAgePossibility", "95")
}
if ((greater("XRAge", "85") || equal("XRAge", "85")) && !greater("XRAge", "89")) {
    add("XFAgePossibility", "100")
}
if ((greater("XRAge", "90") || equal("XRAge", "90")) && !greater("XRAge", "94")) {
    add("XFAgePossibility", "105")
}
if ((greater("XRAge", "95") || equal("XRAge", "95")) && !greater("XRAge", "99")) {
    add("XFAgePossibility", "110")
}
if (greater("XRAge", "100") || equal("XRAge", "100")) {
    add("XFAgePossibility", "115")
}
```

XPsyActType Types of Physical Activities

```
add("XPsyActType", ["Vigorous-intensity activity (Vigorous activities can cause shortness of breath.
↪ Examples of vigorous-intensity activities include carrying heavy stuff, digging, hoeing, aerobic
↪ workout, bicycling at a fast speed, riding a cargo bike/motorcycle, etc.)", "Moderate activity
↪ (Moderate activities can make you breathe faster than usual. Examples of moderate activities include
↪ carrying light stuff, bicycling at a normal speed, mopping, Tai-Chi, and speed walking)", "Mild
↪ activities such as walking (walking from one place to another place at a workplace or home, and taking
↪ a walk for leisure, sports, exercise or entertainment)"])
```

XSocType Types of Social Activities

```
if (selected("DA038","1")) {
     add("XSocType[1]", "Interacted with friends")
}
if (selected("DA038","2")) {
    add("XSocType[2]", "Played Ma-jong, played chess, played cards, or went to community club")
}
if (selected("DA038","3")) {
    add("XSocType[3]", "Provided help to family, friends, or neighbors who do not live with you")
}
if (selected("DA038","4")) {
    add("XSocType[4]", "Went to a sport, social, or other kind of club")
}
if (selected("DA038","5")) {
    add("XSocType[5]", "Took part in a community-related organization")
}
if (selected("DA038","6")) {
```

```
    add("XSocType[6]", "Done voluntary or charity work, or  Cared for a sick or disabled adult who does
     ↪  not live with you")
}
if (selected("DA038","7")) {
    add("XSocType[7]", "Attended an educational or training course")
}
if (selected("DA038","8")) {
    add("XSocType[8]", "Other social activities")
}
```

XHelperSelect Has anyone helped

```
if (equal("DB002", "1") || equal("DB004", "1") || equal("DB006", "1") || equal("DB008", "1") ||
 ↪  equal("DB010", "1") ||equal("DB013", "1") || equal("DB015", "1") || equal("DB017", "1") ||
 ↪  equal("DB019", "1") || equal("DB021", "1")  || equal("DB023", "1")) {
    add("XHelperSelect", "1")
} else {
    add("XHelperSelect", "0")
}
```

XHelperChild Names of Children Who Provide Help

```
for (var i1 = 1; i1 <= 25; i1++) {
    add("XHelperChild[i1]", value("XChildPanAliveName[i1]"))
}
for (var i1 = 26; i1 <= 35; i1++) {
    add("XHelperChild[i1]", value("DB026_1[i1]"))
}
```

XSibName Names of Siblings

```
for (var i1 = 1; i1 <= 15; i1++) {
    if (!empty("ZSibName[i1]")) {
        add("XSibName[i1]", pre("XRName")+"his/her siblings"+pre("ZSibName[i1]"))
    }
}
for (var i1 = 1; i1 <= 15; i1++) {
    if (!empty("ZSibNameS[i1]")) {
        add("XSibName["+(i1+15)+"]", pre("XRName")+"his/her spouse's siblings"+pre("ZSibNameS[i1]"))
    }
}
```

XHelperSib Names of Siblings Who Provide Help

```
for (var i1 = 1; i1 < 31; i1++) {
    add("XHelperSib[i1]", value("XSibName[i1]"))
}
for (var i1 = 31; i1 <= 40; i1++) {
    add("XHelperSib[i1]", value("DB028_1[i1]"))
}
```

XHelperNum Numbers of Helpers

```
add("XHelperNum", "0")
if (selected("DB024", "1")) {
    add("XHelperNum", value("XHelperNum")+1)
    add("XHelper["+value("XHelperNum")+"]", "Spouse")
}
if (selected("DB024", "5")) {
    add("XHelperNum", value("XHelperNum")+1)
    add("XHelper["+value("XHelperNum")+"]", "Other relative")
}

if (selected("DB024", "6")) {
    add("XHelperNum", value("XHelperNum")+1)
    add("XHelper["+value("XHelperNum")+"]", "Paid helper")
}

if (selected("DB024", "7")) {
    add("XHelperNum", value("XHelperNum")+1)
    add("XHelper["+value("XHelperNum")+"]", "Volunteer")
}
```

```
if (selected("DB024", "8")) {
    add("XHelperNum", value("XHelperNum")+1)
    add("XHelper["+value("XHelperNum")+"]", "Employee(s) of facility")
}

if (selected("DB024", "9")) {
    add("XHelperNum", value("XHelperNum")+1)
    add("XHelper["+value("XHelperNum")+"]", "Employee(s) of home-based elderly care institutions")
}

if (selected("DB024", "10")) {
    add("XHelperNum", value("XHelperNum")+1)
    add("XHelper["+value("XHelperNum")+"]", "Community")
}

if (selected("DB024", "11")) {
    add("XHelperNum", value("XHelperNum")+1)
    add("XHelper["+value("XHelperNum")+"]", " Others")
}
if (selected("DB025", "1")) {
    add("XHelperNum", value("XHelperNum")+1)
    add("XHelper["+value("XHelperNum")+"]", "Father")
}
if (selected("DB025", "2")) {
    add("XHelperNum", value("XHelperNum")+1)
    add("XHelper["+value("XHelperNum")+"]", "Mother")
}
if (selected("DB025", "3")) {
    add("XHelperNum", value("XHelperNum")+1)
    add("XHelper["+value("XHelperNum")+"]", "Father-in-law")
}
if (selected("DB025", "4")) {
    add("XHelperNum", value("XHelperNum")+1)
    add("XHelper["+value("XHelperNum")+"]", "Mother-in-law")
}
for (var i1 = 1; i1 < 26; i1++) {
    if (selected("DB026", i1) && selected("DB027[i1]", "1")) {
        add("XHelperNum", value("XHelperNum")+1)
        add("XHelper["+value("XHelperNum")+"]", value("XChildPanAliveName[i1]")+"himself/herself")
    }
    if (selected("DB026", i1) &&  selected("DB027[i1]", "2")) {
        add("XHelperNum", value("XHelperNum")+1)
        add("XHelper["+value("XHelperNum")+"]", value("XChildPanAliveName[i1]")+"his/her spouse")
    }
    if (selected("DB026", i1) &&  selected("DB027[i1]", "3")) {
        add("XHelperNum", value("XHelperNum")+1)
        add("XHelper["+value("XHelperNum")+"]", value("XChildPanAliveName[i1]")+"his/her children")
    }
}
for (var i1 = 26; i1 < 36; i1++) {
    if (selected("DB026", i1) && selected("DB027[i1]", "1")) {
        add("XHelperNum", value("XHelperNum")+1)
        add("XHelper["+value("XHelperNum")+"]", value("DB026_1[i1]")+"himself/herself")
    }
    if (selected("DB026", i1) &&  selected("DB027[i1]", "2")) {
        add("XHelperNum", value("XHelperNum")+1)
        add("XHelper["+value("XHelperNum")+"]", value("DB026_1[i1]")+"his/her spouse")
    }
    if (selected("DB026", i1) &&  selected("DB027[i1]", "3")) {
        add("XHelperNum", value("XHelperNum")+1)
        add("XHelper["+value("XHelperNum")+"]", value("DB026_1[i1]")+"his/her children")
    }
}
for (var i1 = 1; i1 < 31; i1++) {
    if (selected("DB028", i1) &&  selected("DB029[i1]", "1")) {
        add("XHelperNum", value("XHelperNum")+1)
        add("XHelper["+value("XHelperNum")+"]", value("XSibName[i1]")+"himself/herself")
    }
    if (selected("DB028", i1) &&  selected("DB029[i1]", "2")) {
        add("XHelperNum", value("XHelperNum")+1)
        add("XHelper["+value("XHelperNum")+"]", value("XSibName[i1]")+"his/her spouse")
    }
    if (selected("DB028", i1) &&  selected("DB029[i1]", "3")) {
        add("XHelperNum", value("XHelperNum")+1)
```

```
        add("XHelper["+value("XHelperNum")+"]", value("XSibName[i1]")+"his/her children")
    }
}
for (var i1 = 31; i1 < 41; i1++) {
    if (selected("DB028", i1) &&  selected("DB029[i1]", "1")) {
        add("XHelperNum", value("XHelperNum")+1)
        add("XHelper["+value("XHelperNum")+"]", value("DB028_1[i1]")+"himself/herself")
    }
    if (selected("DB028", i1) &&  selected("DB029[i1]", "2")) {
        add("XHelperNum", value("XHelperNum")+1)
        add("XHelper["+value("XHelperNum")+"]", value("DB028_1[i1]")+"his/her spouse")
    }
    if (selected("DB028", i1) &&  selected("DB029[i1]", "3")) {
        add("XHelperNum", value("XHelperNum")+1)
        add("XHelper["+value("XHelperNum")+"]", value("DB028_1[i1]")+"his/her children")
    }
}
```

XHelper Names or Identities of Helpers

```
see above
```

XSelectNum Chose More Than 7 Helpers or Not

```
add("XSelectNum", "0")
for (var i1 = 1; i1 < 99; i1++) {
    if (selected("DB032", i1)) {
        add("XSelectNum", value("XSelectNum")+1)
    }
}
```

XHelpList Main Helpers Been Select

```
for (var i1 = 1; i1 < value("XHelperNum")+1; i1++) {
    if ( greater("XHelperNum", "7") && !equal("XHelperNum", "7") && selected("DB032", i1) ) {
        add("XHelpList[i1]", value("XHelper[i1]"))
    }
}
for (var i1 = 1; i1 < value("XHelperNum")+1; i1++) {
    if ( !greater("XHelperNum", "7") ) {
        add("XHelpList[i1]", value("XHelper[i1]"))
    }
}
```

XHelperCurrent Had Helpers Current

```
if (greater("XHelperNum", "0")) {
    add("XHelperCurrent", "1")
} else {
    add("XHelperCurrent", "0")
}
```

XWordlist Wordlist For Tests

```
if (!empty("XMainR")) {
    add("XWordlist", ["River", "Book", "Eyes", "Cottage", "Pole", "Stamp", "Motorcycle", "Grass", "Egg",
    ↪  "President"])
}
if (empty("XMainR")) {
    add("XWordlist", ["Sky", "Paper", "Arm", "Building", "Stick", "Ticket", "Automobile", "Flower",
    ↪  "Milk", "Prime Minister"])
}
```

XWordRecallBR Done Immediate Wordrecall Test or Not

```
if  (!selected("DC012", "13") && !selected("DC012", "12") && !empty("DC012"))  {
    add("XWordRecallBR", "1")
} else {
    add("XWordRecallBR", "0")
}
```

This page intentionally left blank

F Work and Retirement

F. Proxy Mode Confirmation

proxy_7 IWER: Enter proxy mode?

1. Yes
2. No

FA. Work Status (1): General

【**INTRO**: Now we will ask you some questions about [XRName]'s work and retirement.】

FA001 Did [XRName] engage in agricultural work for at least 10 days in the past year for your own household? Agricultural work includes farming, forestry, fishing, animal production and selling agricultural products produced by your own household.

【**IWER**: The definition of agricultural work should be consistent in both the work module and the income module.】

1. Yes
2. No

FA002 Does [XRName] do agricultural work for own family, or earn wages from others, or both? (Select All That Apply. Don't Read The Answers)

1. For own family
2. Earn wages from others

FA004 If we consider non-agricultural work, did [XRName] work at least one hour last week in paid work, individual business or family business without getting paid?

【**IWER**: The definition of non-agricultural work should be consistent in both the work module and the income module.】

1. Yes
2. No

FA005 Is [XRName] currently engaged in any non-agricultural work but is on vacation, on sick or other leave, or in job training?

1. Yes
2. No

FA007 Can [XRName] return to the original job within a determined period of time or 6 months?

1. Yes
2. No

FA008 Does [XRName] still receive any salaries or incomes from this work?

1. Yes
2. No

FA009 [XPrefixMainJob] Is [XRName] currently engaged in more than one non-agricultural work?

【**IWER**: Gig jobs should not be double counted.】

1. Yes, at least two
2. No, only one

FA010 [XPrefixMainJob] [XRName] has more than one job, what is [XRName]'s main job, that is, the job with the longest working hours? Is this job paid work, individual business, or unpaid help for family business? We will focus on this main job now. (Don't Read The Answers)

【**IWER**: Priority of selecting the main job is given to work that has not been terminated.】

1. Non-agricultural employed
2. Non-agricultural self-employed
3. Unpaid help for family business
4. [XWorkTypeFarmEmployed]

FA011 Is this job paid work, individual business, or unpaid help for family business? We will focus on this main job now. (Don't Read The Answers)

1. Non-agricultural employed
2. Non-agricultural self-employed
3. Unpaid help for family business

FA. Work Status (2): Change

FA013 We were told at the last interview that [XRName] was not working (neither in agriculture) on [ZIWTime], so when did [XRName] start working (including agricultural work) since then?

【**IWER**: If R answers that [XRName] was working at the time of the last interview, please select "Deny the record".

If R confirms the denial, but just cannot answer the month when [XRName] started working, enter "-1" for the month.】

1. ______ [hc([ZIWYear, 2020], *int*, ∅), sc([2018, 2020], *int*, ∅)] (**FA013_1**) Year ______ [hc([1, 12], *int*, −1)] (**FA013_2**) Month

995. Deny the record ([XRName] was working at that time)
999. Refuse

FA014 When we say that [XRName] was not working at that time, we mean that he had not done any agricultural work in the previous year and had not done any non-agricultural work in the previous week before [ZIWTime]. When we say [XRName] is currently working, we mean that he did some agricultural work last year or non-agricultural work last week or is on vacation. Please confirm again, was [XRName] actually working on [ZIWTime]?

【**IWER**: If R thinks the answer that [XRName] is working is incorrect, please go back to the previous section and correct it. If R thinks our record is correct, please go back to the previous question and re-answer when [XRName] started working.】

1. Confirm that [XRName] was working

997. Don't know
999. Refuse

FA015 What was the reason for [XRName] to start to work?

1. Economic reasons, need more income
2. Recovery from poor health
3. No need for caregiving

4. Work as exercise
5. No particular reason, just want to work
6. Other, please specify ______ (**FA015_1**)

FA016 During the last interview we were informed that [XRName] was working on [ZIWTime]. As I said earlier, work in agriculture, working for pay, running a business, and helping out in a family business are all considered work. So from then until now, when did [XRName] stop working?

【**IWER**: If R answers that [XRName] was not working at the time of the last interview, please select "Deny the record". If R confirms that our record is correct, but cannot answer the month when [XRName] stopped working, enter "-1" for the month.】

1. ______ [hc([ZIWYear,XIWYear], int, ∅), sc([2018,XIWYear], int, ∅)] (**FA016_1**) Year ______ [hc([1, 12], int, −1)] (**FA016_2**) Month

995. Deny the record (not working at that time)
999. Refuse

FA017 When we say [XRName] was working on [ZIWTime], we mean that he/she had done agricultural work in the previous year or non-agricultural work in the previous week before [ZIWTime]. When we say [XRName] is not currently working, we mean that he has not done agricultural work last year and has not done non-agricultural work last week. Please confirm again that [XRName] was not working on [ZIWTime]?

【**IWER**: If R thinks the answer of not currently working is inaccurate, please go back and correct it. If R thinks our record is correct, please go back to the previous question and re-answer the time when [XRName] stopped working.】

1. Confirm that [XRName] was not working

997. Don't know
999. Refuse

FA018 Sorry that we recorded wrongly before, so [XRName] was not working on [ZIWTime], then when did [XRName] stop working? ______ [hc([1950, 2020], int, ∅)] (**FA018_1**) Year ______ [hc([1, 12], int, −1)] (**FA018_2**) Month

【**IWER**: If R cannot answer the month, enter "-1".】

FA019 What was the reason for [XRName] to stop working?

1. Forced to leave, e.g. being fired
2. Bad business or poorly paid
3. Non-pecuniary reason
4. Health issues
5. Family issues, e.g. caregiving
6. Formal retirement
7. Temporary layoff
8. Other, please specify ______ (**FA019_1**)

FB. Self-Employed Agricultural Work

【**INTRO**: Now we will ask you some questions about [XRName]'s work in household agriculture.】

FB001 Where is [XRName]'s workplace for agricultural work in the past year?

【**IWER**: If R does not know the street district and community, select as appropriate.】

1. The same village or community with the current residence: [XRResidenceFull]
2. The same county or district with the current residence, [XRResidenceCounty], but in other village or community: ______ (**FB001_1**) township/town/street district/village/community
3. Other county or district: ______ (**FB001_2**) province/city/county ______ (**FB001_3**) township/town/street district/village/community
4. Areas where none of the above options apply (Hong Kong, Macao, Taiwan, or abroad)

999. Refuse

FB002 Which of the following types of agriculture does [XRName]'s work belong to? (Select All That Apply)

1. Plantation
2. Forestry
3. Animal husbandry
4. Fishery
5. Professional or auxiliary agriculture, forestry, animal husbandry and fishery activities

997. Don't know

[conflict$(997, [997]^c)$]

FB003 What kind of work does [XRName] mainly do?

1. Manual labor
2. Management
3. Machine operation
4. Purchasing and sales
5. Others

FB005 How many months did [XRName] work for own household agriculture in the past year? ______ [hc$([1, 12], int, \varnothing)$] months

FB006 How many days did [XRName] work for own household agriculture per week on average during a normal month in the past year? ______ [hc$([1, 7], int, \varnothing)$] days

【**IWER**: If less than four days per month and the average is less than one day per week, enter "1".】

FB007 How many hours did [XRName] usually work for own household agriculture during a normal work day in the past year? ______ [hc$([1, 24], int, \varnothing)$], sc$([1, 16], int, \varnothing)$] hours

FC. Employed Work (1): General

【**INTRO**: Next, I would like to know more about [XRName]'s paid job that we mentioned earlier. If [XRName] is doing gig jobs and does not have a fixed employer, [XRName]'s "employer" relates to either the current job or the most recent job. 】

FC001 Does [XRName] receive wages from [XRName]'s current employer, or from a dispatch/contract company, or some individual? (Don't Read The Answers)

1. Workplace
2. Dispatch Company
3. Individual

FC002 What is the type of [XRName]'s workplace/employer? [XFC002]

1. Government
2. Public institution
3. NGO
4. Firm
5. Individual firm
6. Farmer
7. Individual household
8. Others, please specify ______ (**FC002_1**)

997. Don't know

FC003 At the time of last visit on [ZIWTime], [XRName]'s [XWorkplace] was [ZFD003], is it still [XR-Name]'s current [XWorkplace]?

【**IWER**: If the employer does not have department, enter "None" for the department name】

1. Yes. Name of department: ______ (**FC003_2**)
2. No

995. Deny the record (same [XWorkplace] but not recorded correctly)

FC003_1 What is the name of [XRName]'s [XFC003]?

Name of employer: ______ (**FC003_1**)

Name of department: ______ (**FC003_2**)

【**IWER**: If the employer does not have department, enter "None" for the department name】

FC004 Where is [XRName]'s employer located?

【**IWER**: If R does not know the street district and community, select as appropriate 】

1. The same village or community with the current residence: [XRResidenceFull]
2. The same county or district with the current residence, [XRResidenceCounty], but in other village or community: ______ (**FC004_1**) township/town/street district/village/community
3. Other county or district: ______ (**FC004_2**) province/city/county ______ (**FC004_3**) township/town/street district/village/community
4. Areas where none of the above options apply (Hong Kong, Macao, Taiwan, or abroad)

999. Refuse

FC005 What industry does [XRName]'s [XWorkplace] belong to? In other words, what product does [XWorkplace] make or what service does it provide? ______

【**IWER**: Please follow the Guide for Industry and Occupation】

FC006 Is [XRName] civil servant?

1. Yes
2. No

FC008 Is [XRName] in the establishment?

1. Yes

2. No

FC011 What sort of work does [XRName] do? ______

FC012 Is there anyone people under [XRName]'s supervision?

1. Yes
2. No

FC016 Did [XRName] sign a labor contract in written form with [XRName]'s [XEmployer] ?

1. Yes
2. No

FC017 What is the agreed period of employment (labor contract period)?

【**IWER**: If answered with defined period, but the exact year and month cannot be remembered, enter "-1" for year and month.】

1. Defined period ______ [hc$([0,50], int, -1)$] (**FC017_1**) years and ______ [hc$([0,12], int, -1)$] (**FC017_2**) months
2. Undefined period
3. Same as the term of the project

997. Don't know
999. Refuse

FC018 I remember that [XRName] started working for [XWorkplace] in the year of [ZFD011_1]. If this is incorrect or there was a break in employment, please tell us when this employment started.

【**IWER**: If year or month cannot be remembered, enter "-1".】

1. Correct
2. Incorrect, the work started since ______ [hc$([1950,$XIWYear$], int, -1)$] (**FC018_1**) Year ______ [hc$([1,12], int, -1)$] (**FC018_2**) Month

999. Refuse

FC019 When did [XRName] start working for [XWorkplace]? ______ [hc$([1950,$XIWYear$], int, -1)$] (**FC019_1**) Year ______ [hc$([1,12], int, -1)$] (**FC019_2**) Month

【**IWER**: If year or month cannot be remembered, enter "-1".】

FC025 Including paid vacations and sick leave fully paid, how many months did [XRName] work on this job in the past year? ______ [hc$([1,12], int, \varnothing)$] months

【**IWER**: SPECIAL CASE: For this job, if R has been on vacation for the past whole year and does not receive any pay, enter "1" for the number of months, days per week, and hours per week worked.】

FC026 How many days a week did [XRName] work on average in the past year? The weeks exclude paid vacations and sick leave fully paid. ______ [hc$([1,7], int, \varnothing)$] days

【**IWER**: If less than four days per month and the average is less than one day per week, enter "1".】

FC027 How many hours did [XRName] work per day on average in the past year, excluding meal breaks but including any paid or unpaid overtime? ______ [hc$([1,24], int, \varnothing)$], sc$([1,16], int, \varnothing)$] hours

FC. Employed Work (2): Wages

FC032 How is [XRName]'s wage paid mainly? Is it regularly paid, contract-based, performance-based, or other? If it is regularly paid, please tell me how often [XRName] receives wages. Is [XRName]'s wage receipt schedule yearly, monthly, weekly, daily, or hourly?
If the employer owes wages or [XRName] has just started the work, please tell us about the wage to be paid. (Don't Read The Answers)
【**IWER**: Wages owed but yet received are wages that can be received in the future. If the prospect of wages to be paid is very unlikely, they cannot be counted as wages owed.】

1. Yearly wages
2. Monthly wages
3. Weekly wages
4. Daily wages
5. Hourly wages
6. Contract-based
7. Performance-based
8. Other, please specify ______ (**FC032_1**)

997. Don't know
999. Refuse

FC033 You said that [XRName] has been working on this job in the past year for [FC025] months. Since [XRName]'s wages are paid annually and may not have been received yet, what should the amount of wages for this month from [XEmployer] be? Please include all kinds of earnings such as bonuses, tips, and gifts received at work. ______ [hc([0, 10000000], *int*, −1), sc([1000, 1000000], *int*, −1), ub([−1], [10000, 30000, 50000, 100000, 200000])] Yuan
【**IWER**: If R cannot answer, enter "-1".】

FC034 How much did [XRName] get paid from [XEmployer] last month? [XPrefixMonthlyWage], please include all kinds of earnings such as bonuses, tips, and gifts received at work. ______ [hc([0, 10000000], *int*, −1), sc([100, 100000], *int*, −1), ub([−1], [500, 1000, 2500, 5000, 10000])] Yuan
【**IWER**: If R cannot answer, enter "-1".】

FC035 How much did [XRName] get paid from [XEmployer] last week? Please include all kinds of earnings such as bonuses, tips, and gifts received at work. ______ [hc([0, 10000000], *int*, −1), sc([100, 100000], *int*, −1), ub([−1], [100, 300, 500, 1000, 2000])] Yuan
【**IWER**: If R cannot answer, enter "-1".】

FC036 How much did [XRName] get paid generally from [XEmployer] per day? Please include all kinds of earnings such as bonuses, tips, and gifts received at work. ______ [hc([1, 10000000], *int*, −1), sc([100, 100000], *int*, −1), ub([−1], [20, 50, 100, 200, 500])] Yuan
【**IWER**: If R cannot answer, enter "-1".】

FC037 How much did [XRName] get paid generally from [XEmployer] per hour? Please include all kinds of earnings such as bonuses, tips, and gifts received at work. ______ [hc([1, 10000000], *int*, −1), sc([10, 100000], *int*, −1), ub([−1], [10, 30, 50, 100, 200])] Yuan
【**IWER**: If R cannot answer, enter "-1".】

FC038 Then, how much did [XRName] get paid in total from [XEmployer] last month? [XPrefixMonthlyWage] Please include all kinds of earnings such as bonuses, tips, and gifts received at work. ______ [hc([0, 10000000], *int*, −1), sc([100, 100000], *int*, −1), ub([−1], [500, 1000, 2500, 5000, 10000])] Yuan

【IWER: If R cannot answer, enter “-1”.】

FC039 What is the amount of all [XRName]'s other bonuses (not paid regularly as [XRName]'s wages) received from [XEmployer] in the past year? ______ [hc([0, 10000000], *int*, −1), sc([0, 1000000], *int*, −1), ub([−1], [1000, 3000, 5000, 10000, 20000])] Yuan

【IWER: If R cannot answer, enter “-1”.】

FC040 Some people have to pay personal income tax, pension insurance, medical insurance, housing provident fund or other miscellaneous fees before they get their wages. Have these contributions been deducted from the wages mentioned above?

1. Payable, already deducted
2. Payable, not deducted
3. Not payable

997. Don't know
999. Refuse

FC041 How much [XPrefixFC041]?

【IWER: If R have difficulty reporting the exact amount of tax and insurance, you can enter a percentage for the first option, to the nearest ten digits.】

1. ______ [hc([1, 50], *int*, ∅)] (**FC041_1**) percent of wages
2. ______ [hc([100, 100000], *int*, ∅)] (**FC041_2**) Yuan/month
3. ______ [hc([100, 100000], *int*, ∅)] (**FC041_3**) Yuan/year

997. Don't know

FC042 What benefits does [XRName]'s [XEmployer] provide? (Select All That Apply) How much is each benefit worth per month?

【IWER: The benefits should be calculated on a net basis, which is the difference between the market price of the benefit item and the actual cost. For example, if an employee pays 500 Yuan per month to live in a unit dormitory and the market rent for the same condition is 2,000 Yuan, the net value is 1,500 Yuan.

If the net value cannot be estimated, you can enter “-1”.】

1. Meal benefits with net value ______ [hc([10, 10000], *int*, −1), ub([−1], [100, 500, 1000])] (**FC042_1**) Yuan
2. Transportation benefits with net value ______ [hc([10, 50000], *int*, −1), ub([−1], [100, 500, 1000])] (**FC042_2**) Yuan
3. Housing benefits with net value ______ [hc([10, 100000], *int*, −1), ub([−1], [500, 2000, 5000])] (**FC042_3**) Yuan
4. Other benefits not mentioned above with net value ______ [hc([10, 100000], *int*, −1), ub([−1], [100, 500, 1000])] (**FC042_4**) Yuan
5. No benefits (EXCLUSIVE)

999. Refuse

[conflict(5, 999, $[5, 999]^c$)]

FD. Non-Agricultural Self-Employed Work

【**INTRO**: Next I want to know about that [XSelfEmplType] we talked about.】

FD002 Where is [XRName]'s [XSelfEmplName] located?

【**IWER**: If R does not know the street district and community, select as appropriate 】

1. The same village or community with the current residence: [XRResidenceFull]
2. The same county or district with the current residence, [XRResidenceCounty], but in other village or community: ______ (**FD002_1**) township/town/street district/village/community
3. Other county or district: ______ (**FD002_2**) province/city/county ______ (**FD002_3**) township/town/street district/village/community
4. Areas where none of the above options apply (Hong Kong, Macao, Taiwan, or abroad)

999. Refuse

FD003 What industry does [XRName]'s [XSelfEmplName] belong to? In other words, what product does it make or what service does it provide? ______

FD004 How many people are hired in [XRName]'s [XSelfEmplName]? ______ [hc$([0, 9999], int, \varnothing)$] persons (Enter "0" if not hiring anyone.)

FD007 How many months did [XRName] [XSelfEmplVerb] in the past year? ______ [hc$([1, 12], int, \varnothing)$] months

FD008 How many days a week did [XRName] [XSelfEmplVerb] on average in the past year? ______ [hc$([1, 7], int, \varnothing)$] days

【**IWER**: Enter "1" if working no more than 4 days per month.】

FD009 How many hours did [XRName] [XSelfEmplVerb] per day on average in the past year, ______ [hc$([1, 24], int, \varnothing)$, sc$([1, 16], int, \varnothing)$] hours

FE. Side Jobs

【**INTRO**: [XPrefixMainJob] You said earlier that [XRName] has multiple jobs, that is, in addition to his/her main job, [XRName] has [XSuffixMainJob] other jobs.】

FE001 Now let's consider all other jobs together. How many days a week does [XRName] usually work? ______ [hc$([0, 7], real, \varnothing)$, sc$((0, 7], real, \varnothing)$] days

FE002 For these "all other jobs", how many hours does [XRName] usually work per day? ______ [hc$([1, 24], int, \varnothing)$, sc$([1, 16], int, \varnothing)$] hours

FF. Job Search

FF001 On how many days in the past year has [XRName] worked? Agricultural work, working for pay, running business, and helping with family business are all considered work. Here we are asking about the days [XRName] actually worked, not including days off.

______ [hc([0, 366], *int*, −1), ub([−1], [10, 50, 100, 150, 250])] days (If R cannot answer, enter "-1". If [XRName] didn't work, enter "0".)

【IWER:

1. If the distribution of days worked in a year was even, we might have an estimate that R is known to have worked approximately [XHhldFarmDays] days in agricultural self-employment, approximately [XMainJobDays] days in the main job, and approximately [XSideJobDays] days in side jobs. These estimates are for reference only and cannot be directly summed. Note that one may be working multiple jobs on the same day. There may be overlap in the number of days worked in different jobs. [XFF001]

2. Answers can be approximated to the nearest ten days.

3. If you find any problems with the previous answers for work hours, please go back to the corresponding sub-module to correct the answer.】

FF002 Is [XRName] currently engaged in any pastime work and still get some incomes from doing it?

1. Yes
2. No

FF003 Did [XRName] search for a new job during the past month?

1. Yes
2. No

FF004 At what age does [XRName] plan to stop working, i.e. stop earning wages or working for family business without pay or engaging in any other work more serious than pastime work?

【IWER:The age when [XRName] will stop working should be equal to or greater than R's current age.】

1. Stop working at the age of ______ [hc([XRAge, 120], *int*, −1), sc([60, 100], *int*, −1)] (**FF004_1**) years old
2. Stop working after ______ [hc([0, 50], *int*, −1)] (**FF004_2**) years
3. Work as long as health permits

997. Don't know
999. Refuse

FF005 Imagine that [XRName] might have an idea of how old he would stop working if the COVID-19 pandemic had not happened, but the situation might have changed because of the pandemic. So, was [XRName]'s planned time to stop working delayed by the pandemic, brought forward, or remained unchanged?

【IWER: If there is a change of a few months, but less than a year, it can be rounded up to a year. If R cannot answer the specific number of years, you can enter "-1". If R gives an answer of working non-stop regardless of the pandemic as long as health permits, select "Not affected" in this case.】

1. Brought forward by ______ [hc([1, 50], *int*, −1)] (**FF005_1**) years
2. Delayed by ______ [hc([1, 50], *int*, −1)] (**FF005_2**) years
3. Not affected

997. Don't know
999. Refuse

FG. COVID Impact

【**INTRO**: I would like to know the impact of the COVID-19 pandemic on [XRName]'s work.】

FG001 During the pandemic, some workers used remote work techniques such as web- or tele-conferencing. So during the pandemic, did [XRName] ever work remotely or work at home?

1. Yes
2. No

FG002 Was there a situation that [XRName] experienced during the pandemic that there was a period of time [XVCNotInQuarantine] when [XRName]'s boss or company was not open for work, but he/she could get paid for not working?

【**IWER**: If R cannot answer, enter "-1".】

1. Yes, the average wage received in this situation is about ______ [hc$([1, 100], int, -1)$] (**FG002_1**) % of the normal wage, for a total of ______ [hc$([1, 50], int, -1)$] (**FG002_2**) weeks
2. Yes, the average wage received in this situation is higher than the normal wage
3. No such situation

FG003 During the pandemic, some people were restricted from going to the field for agricultural work. Imagine that if the pandemic had not happened, [XRName] would have been doing agricultural work for own household, but because of the pandemic, the situation may have changed. So, has the number of days that [XRName] did agricultural work for own household increased, decreased, or not been affected by the pandemic since the Chinese New Year? Agricultural work were just described, including farming, forestry, fishing, animal production and selling home-grown agricultural products.

【**IWER**: If R cannot answer the number of weeks, enter "-1".】

1. Would not do agricultural work for own household with or without the pandemic
2. Worked less, for ______ [hc$([1, 40], int, -1)$] (**FG003_1**) weeks
3. Worked more, for ______ [hc$([1, 40], int, -1)$] (**FG003_2**) weeks
4. Not affected

999. Refuse

FG004 During the pandemic, some people who had been working delayed their return to work or lost their jobs. Imagine that if the pandemic had not happened, [XRName] would have gone to work for pay, but because of the pandemic, the situation may have changed. So, since the Chinese New Year, has the number of days [XRName] worked for pay increased, decreased, or not been affected by the pandemic?

【**IWER**: If R cannot answer the number of weeks, enter "-1".】

1. Would not go to work for pay with or without the pandemic
2. Worked less, for ______ [hc$([1, 40], int, -1)$] (**FG004_1**) weeks
3. Worked more, for ______ [hc$([1, 40], int, -1)$] (**FG004_2**) weeks
4. Not affected

999. Refuse

FG005 During the pandemic, some stores could not open, and when they did, they had no

business. Imagine that if the pandemic had not happened, [XRName] may have been running business, but because of the pandemic, the situation may have changed. So, has the number of days [XRName] running business increased, decreased, or not been affected by the pandemic since the Chinese New Year? Running business includes free-lancing and not getting paid to help out in a family business, and does not include agricultural work.

【**IWER**: If R cannot answer the number of weeks, enter “-1”.】

1. Would not run business with or without the pandemic
2. Worked less, for ______ [hc([1, 40], *int*, −1)] (**FG005_1**) weeks
3. Worked more, for ______ [hc([1, 40], *int*, −1)] (**FG005_2**) weeks
4. Not affected

999. Refuse

FG008 During the pandemic, some people worked only half days; if there had not been the pandemic, they might have been working full days. For [XRName], the situation may have changed because of the pandemic as well. So, from the Chinese New Year to now, in the months of working, has the pandemic increased, decreased, or not affected the number of hours worked per week for [XRName]?

【**IWER**: The days that R did not work should not be considered here; the decrease in days and the decrease in hours should not be double-counted. If the impact of the pandemic resulted in R not working a whole day, this situation should be recorded as a decrease in the number of days. If R is unable to answer a specific number of hours, enter “-1”.】

1. No effect
2. Worked less, for ______ [hc([1, 40], *int*, −1)] (**FG008_1**) hours
3. Worked more, for ______ [hc([1, 40], *int*, −1)] (**FG008_2**) hours
4. Hasn’t worked since the Chinese New Year

999. Refuse

FG009 Did [XRName] ever receive unemployment insurance benefits during the pandemic?

1. Yes
2. No

FG010 [XRName] received unemployment insurance benefits for ______ [hc([1, 10], *int*, −1)] (**FG010_1**) months. The average amount of unemployment insurance benefits received per month is ______ [hc([1, 10000], *int*, −1)] (**FG010_2**) Yuan.

【**IWER**: If R cannot answer, enter “-1”.】

FH. Retirement

FH001 Has [XRName] completed retirement procedures, including early retirement or internal retirement? (Note: Retirement refers to the pension-eligible retirement from a position in the government, public institutions, and firms, also including the procedure done by individuals from the informal sector who have contributed a lump sum to the Urban Employee Pension Insurance.)

【**IWER**: Starting to receive pensions from the Urban Resident Pension Insurance, the New Rural Pension Insurance, or the Urban and Rural Resident Pension Insurance is not considered to have

gone through retirement procedures.】

1. Yes
2. No

FH002 Is it normal retirement, early retirement, formal retirement after internal retirement, or formal retirement to be processed after internal retirement? (Don't Read The Answers)

1. Formal retirement
2. Early retirement
3. Internal retirement first then formal retirement
4. Internal retirement but not yet formal retirement

FH003 In which month and year was [XRName]'s formal retirement processed? ______ [hc([1950,XIWYear], *int*, −1), sc([ZIWYear,XIWYear], *int*, −1)] (**FH003_1**) Year ______ [hc([1, 12], *int*, −1)] (**FH003_2**) Month

【**IWER**: If R cannot answer, enter "-1".】

FH004 In which month and year will [XRName]'s formal retirement be processed? ______ [hc([XIWYear, 2050], *int*, −1)] (**FH004_1**) Year ______ [hc([1, 12], *int*, −1)] (**FH004_2**) Month

【**IWER**: If R cannot answer, enter "-1".】

Auxiliary Variable Definition

XWorking Whether working or not

```
if (equal("FA001", "1")  || equal("FA004", "1") || equal("FA007", "1") || equal("FA008", "1")) {
    add("XWorking", "1")
} else {
    add("XWorking", "0")
}
```

XEmployed Whether self-employed or not

```
if (equal("FA010", "2") || equal("FA010", "3") || equal("FA011", "2") || equal("FA011", "3")) {
    add("XEmployed", "0")
} else if (equal("FA010", "1") || equal("FA010", "4") || equal("FA011", "1") || selected("FA002", "2")) {
    add("XEmployed", "1")
}
```

XFGSample Whether to be asked employment questions during the pandemic

```
if (equal("XWorking","1")) {
    add("XFGSample","1")
} else if (equal("FF003","1")) {
    add("XFGSample","1")
} else if (equal("FF005","1") || equal("FF005","2")) {
    add("XFGSample","1")
} else if (equal("FA016_1","2020")) {
    add("XFGSample","1")
} else {
    add("XFGSample","0")
}
```

XPrefixMainJob Leading words for main job questions

```
if (selected("FA002","1")) {
    add("XPrefixMainJob", "Not considering agricultural work for own household,")
}
```

XSuffixMainJob Trailing words for main job questions

```
if (selected("FA002","2")) {
    add("XSuffixMainJob", "agricultural work for pay and")
}
```

XEmployer The actual employer from whom wages get paid

```
if (equal("FC001","2")) {
    add("XEmployer", "dispatch company")
} else if (equal("FC001","3")) {
    add("XEmployer", "contractor")
} else if (equal("FC001","1") && greater("FC002","4")) {
    add("XEmployer", "individual employer")
} else {
    add("XEmployer", "employer")
}
```

XWorkplace Employer WORDING

```
if (greater("FC002","4")) {
    add("XWorkplace", "individual employer")
} else {
    add("XWorkplace", "employer")
}
```

XSelfEmplName Name of self-employed business

```
if (equal("FA010","2") || equal("FA011","2") ) {
    add("XSelfEmplName","busines")
    add("XSelfEmplType","business")
    add("XSelfEmplVerb","run the busines")
} else {
    add("XSelfEmplName","family business")
    add("XSelfEmplType","family business")
    add("XSelfEmplVerb","help out the family business")
}
```

XSelfEmplType Type of self-employed business

```
see above
```

XSelfEmplVerb Verb for self-employed business

```
see above
```

XFC002 FC002 WORDING

```
if (equal("FC001","2")) {
    add("XFC002", "Note that it is the employer, not the dispatcher.")
} else if (equal("FC001","3")) {
    add("XFC002", "Note that it is the employer, not the contractor.")
}
```

XFC003 FC003 WORDING

```
if (greater("FC002","4")) {
    add("XFC003", "employer")
} else {
    add("XFC003", "individual employer")
}
```

XWorkJustStarted Whether the job for pay started no more than a year ago, or no more than a month ago.

```
if ((equal("FC019_1", "2020") && greater("FC019_2", value("XIWMonth")-2)) || (equal("FC018_1", "2020") &&
↪ greater("FC018_2", value("XIWMonth")-2))) {
    add("XWorkJustStarted", "1")
} else if ((equal("FC019_1", "2020") || (equal("FC019_1", "2019") && greater("FC019_2",
↪ value("XIWMonth")-1))) || (equal("FC018_1", "2020") || (equal("FC018_1", "2019") && greater("FC018_2",
↪ value("XIWMonth")-1)))) {
    add("XWorkJustStarted", "2")
```

```
} else {
    add("XWorkJustStarted", "0")
}
```

XMainJobDays Days in the main job

```
if (equal("XEmployed","1")) {
    add("XMainJobDays", value("FC026")*52)
} else if (equal("XEmployed","1")) {
    add("XMainJobDays", value("FD008")*52)
} else {
    add("XMainJobDays", "0")
}
```

XHhldFarmDays Days in agricultural self-employment

```
add("XHhldFarmDays", value("FB006")*value("FB005")*4)
if (empty("XHhldFarmDays")) {
add("XHhldFarmDays", "0")
}
```

XSideJobDays Days in side jobs

```
add("XSideJobDays", value("FE001")*52)
if (empty("XSideJobDays")) {
    add("XSideJobDays", "0")
}
```

XFF001 FF001 notice for IWER

```
if (equal("XWorking","0")) {
    add("XFF001", "Also note that R may have worked in a non-agricultural job earlier in the past year,
    ↪ although not currently working.")
} else if (empty("XEmployed")) {
    add("XFF001", "Also note that R may have worked in a non-agricultural job earlier in the past year,
    ↪ although not currently working in a non-agricultural job.")
} else if (greater("12", "FC025")) {
    add("XFF001", "Also note that although R is currently working in a non-agricultural job, he or she may
    ↪ have worked in that job for less than a month in the past year and may not have been working or
    ↪ may have been working in a different job the rest of the year.")
}
```

XGetJob Whether from non-working to working

```
if (equal("ZWorking","0") && equal("XWorking","1")) {
    add("XGetJob", "1")
} else {
    add("XGetJob", "0")
}
```

XQuitJob Whether from working to non-working

```
if (equal("ZWorking","1") && equal("XWorking","0")) {
    add("XQuitJob", "1")
}else {
    add("XQuitJob", "0")
}
```

XPrefixMonthlyWage WORDING for those who have just started working, when asked for last month's salary

```
if (equal("XWorkJustStarted", "1")) {
    add("XPrefixMonthlyWage", "If you have just started working and have not been paid yet, please tell us
    ↪ how much you will be paid next month.")
}
```

XPrefixFC041 FC041 WORDING

```
if (equal("FC040", "1")) {
    add("XPrefixFC041", "was deducted")
} else {
    add("XPrefixFC041", "will be deducted when wages received")
}
```

XWorkTypeFarmEmployed Answer choice of “being farm employed” in the main job question

```
if (selected("FA002","2")) {
    add("XWorkTypeFarmEmployed", "Farm employed")
}
```

XIWYear Year of this interview

XIWMonth Month of this interview

ZFD003 Employer name at the last interview

ZFD011_1 The year when R started working for R’s employer at the last interview

ZWorking Work status at the last interview

ZRRetired Retirement status at the last interview

G Income and Expenditure

G1 Household Income and Expenditure

G1. Proxy Mode Confirmation

proxy_12 Interviewer record: Enter proxy mode?

1. Yes
2. No

GB.Other Household Member's Wage Income and Individual-based Transfers

GB001 Please pick one member from the followings as financial respondent who would be most familiar with the financial condition of the household?

【**IWER**: If household income, expenditure and housing modules uses proxy, please apply/use [XFL-HHFinancialNameList] individual proxy, and select [XFLHHFinancialNameList] in this question.】

1-25. [XHHMemberName[i]]
26. [XMainR]
27. [XMainRS]

【**INTRO**: Make sure no others are at present. We'd like to ask you some questions regarding the incomes of OTHER members in your household. Your answers will be kept strictly confidential and will only be used for academic research.】

GB002[i] In the past year, did [XHHMemberName[i]] receive any wage and bonus income, excluding retirement, receding and internal retirement income? Here wage is from all employments.

1. Yes
2. No

997. Don't know
999. Refuse

GB003[i] How much did [XHHMemberName[i]] receive last year? ______ [hc$((0,\infty), int, -1)$, sc$((0,240000], int, -1)$, ub$([-1],[5000,10000,30000,50000,100000])$] Yuan

【**IWER**: Please enter "-1" if R cannot answer.】

GB004[i] Does the above mentioned wage [XHHMemberName[i]] exclude any insurance, income tax, public housing funds and other fees?

1. Yes
2. No

997. Don't know
999. Refuse

GB005[i] What is the total amount of [XHHMemberName[i]] [XGB005Text[i]]'s insurance, income tax, public housing funds and other fees?

【**IWER**: Please enter "-1" if R cannot answer.】

1. ______ [hc$((0,\infty), int, -1)$, sc$((0,10000], int, -1)$, ub$([-1],[300,500,1000,2000,3000])$] (**GB005_1[i]**) Yuan/Month

2. ______ [hc$((0,\infty), int, \varnothing)$, sc$((0,100000], int, \varnothing)$] (**GB005_2[i]**) Yuan/Year
3. About ______ [hc$((0,100], real, \varnothing)$] (**GB005_3[i]**) % of wage
4. None, 0 Yuan

GB006[i] Did [XHHMemberName[i]] receive any of the following types of individual transfer income in the past year? (select all that apply) Individual COVID-19 pandemic subsidies are also individual transfer incomes.

【**IWER**: Please enter "-1" if R cannot answer.】

1. Pensions, including pension incomes from governments, public institutions and firms, supplemental pension of the firms, receding and internal retirement compensation and incomes from such programs as rural pension insurance, Urban residents' pension, commercial pension insurance, new rural social pension insurance, life insurance and pension for land-expropriated farmers ______ [hc$((0,\infty), int, -1)$, sc$((0,100000], int, -1)$] (**GB006_1[i]**) Yuan
2. Unemployment compensation ______ [hc$((0,\infty), int, -1)$, sc$((0,50000], int, -1)$] (**GB006_2[i]**) Yuan
3. Pension voucher ______ [hc$((0,\infty), int, -1)$, sc$((0,50000], int, -1)$] (**GB006_3[i]**) Yuan
4. Pension subsidy for the oldest old ______ [hc$((0,\infty), int, -1)$, sc$((0,50000], int, -1)$] (**GB006_4[i]**) Yuan
5. Workers' compensation from industrial accident compensation insurance includes wage-replacement benefits, disability benefits, and survivors' benefits ______ [hc$((0,\infty), int, -1)$, sc$((0,50000], int, -1)$] (**GB006_5[i]**) Yuan
6. Elderly family planning subsidies ______ [hc$((0,\infty), int, -1)$, sc$((0,50000], int, -1)$] (**GB006_6[i]**) Yuan
7. Medical aid ______ [hc$((0,\infty), int, -1)$, sc$((0,100000], int, -1)$] (**GB006_7[i]**) Yuan
8. Other government subsidies, excluding Dibao, Wubao, tekun and poor subsidies, please specify ______ (**GB006_8_1[i]**), ______ [hc$((0,\infty), int, -1)$, sc$((0,100000], int, -1)$] (**GB006_8[i]**) Yuan
9. Other social income sources, please specify ______ (**GB006_9_1[i]**), ______ [hc$((0,\infty), int, -1)$, sc$((0,100000], int, -1)$] (**GB006_9[i]**) Yuan
10. None of the above

[conflict$(10, [10]^c)$]

GB007[i] Without COVID-19 pandemic, [XHHMemberName[i]] may/may not have wage. After breakout of COVID-19, thing may change. From Chinese New year to now, [XHHMemberName[i]]'s wages are increased, decreased or unaffacted because of COVID-19 pandemic?

【**IWER**: Please enter "-1" if R cannot answer.】

1. Decreased ______ [hc$((0,\infty), int, -1)$, sc$((0,50000], int, -1)$, ub$([-1], [1000, 3000, 5000, 10000, 20000])$] (**GB007_1[i]**) Yuan
2. Increased ______ [hc$((0,\infty), int, -1)$, sc$((0,50000], int, -1)$, ub$([-1], [1000, 3000, 5000, 10000, 20000])$] (**GB007_2[i]**) Yuan
3. Unaffected

GB008[i] Did [XHHMemberName[i]] receive any of the following types of individual transfer income in the past year? (select all that apply) Individual COVID-19 pandemic subsidies

are also individual transfer incomes.

【IWER: Please enter "-1" if R cannot answer.】

7. Medical aid ______ [hc($(0,\infty)$, int, -1), sc($(0,50000]$, int, -1)] (**GB008_7[i]**) Yuan
8. Other government subsidies, excluding Dibao, Wubao, tekun and poor subsidies, please specify ______ (**GB008_8_1[i]**), ______ [hc($(0,\infty)$, int, -1), sc($(0,50000]$, int, -1)] (**GB008_8[i]**) Yuan
9. Other social income sources, please specify ______ (**GB008_9_1[i]**), ______ [hc($(0,\infty)$, int, -1), sc($(0,50000]$, int, -1)] (**GB008_9[i]**) Yuan
10. None of the above

[conflict(10, $[10]^c$)]

GC.Household Agricultural Income and Expenditure

【INTRO: Next we will ask some questions about agricultural income and expenditure in your household. Agricultural work in individual questionnaire is about respondent, here is about household level. Even if respondent did not have agricultural work but other household members had, then household had agricultural work.】

GC001 In the past year, did [XMainR]'s household members, including [XFLHHNameList], engage in agricultural activities (including cropping, forestry, livestock, and fish), or selling agricultural products you produced at market?

1. Yes
2. No

GC002 Who engaged in agricultural work in the past year? (select all that apply)

1-25. [XHHMemberName[i]]
26. [XMainR]
27. [XMainRS]

GC003 Did [XMainR]'s household members, including [XFLHHNameList], engage in cropping or forestry last year, including planting flowers, vegetables, various crops, mushrooms, black fungi or collection of wild agricultural and forestry products?

1. Yes
2. No

GC004 What is the net income of all crops and forestry products produced and collection of wild agricultural and forestry products in the past year? Earn or loss money? Net income is total value of output minus input. Output includes selling, own consumption and storage. Input includes seeds, fertilizer, pesticide, plastic sheets, hiring labor, land rents, rents (excluding land rents), irrigation, fuel, transportation, processing, packaging and management fee.

【IWER: Please enter "-1" if R cannot answer.】

1. Earn money, net income ______ [hc($(0,\infty)$, int, -1), sc($(0,100000]$, int, -1), ub($[-1]$, $[2000, 5000, 10000, 50000, 100000]$)] (**GC004_1**) Yuan
2. Loss money, net income ______ [hc($(0,\infty)$, int, -1), sc($(0,100000]$, int, -1), ub($[-1]$, $[2000, 5000, 10000, 50000, 100000]$)] (**GC004_2**) Yuan
3. Break-even

GC005 Did [XMainR]'s household members, including [XFLHHNameList], raised any livestock or fish in the past year?

1. Yes
2. No

GC006 What is the net income of livestock and aquatic life in the past year? Earn or loss money? Net income is total value of output minus input. Output includes final and intermediate products that are sold, own consumed and stored. Input includes feeding fees, medical expenses, grazing fees, barn fence fees, and labor costs, etc.

【**IWER**: Please enter "-1" if R cannot answer.】

1. Earn money, net income ______ [hc$((0,\infty), int, -1)$, sc$((0,100000], int, -1)$, ub$([-1],[2000,5000,10000,50000,100000])$] (**GC006_1**) Yuan
2. Loss money, net income ______ [hc$((0,\infty), int, -1)$, sc$((0,100000], int, -1)$, ub$([-1],[2000,5000,10000,50000,100000])$] (**GC006_2**) Yuan
3. Break-even

GC007 Without COVID-19 pandemic, [XMainR]'s household members, including [XFLHHNameList], may/may not have agricultural incomes. After breakout of COVID-19, thing may change. From Chinese New year to now, the agricultural net incomes of [XMainR]'s household are increased, decreased or unaffacted because of COVID-19 pandemic?

【**IWER**: Please enter "-1" if R cannot answer.】

1. Decreased ______ [hc$((0,\infty), int, -1)$, sc$((0,10000], int, -1)$, ub$([-1],[200,500,1000,5000,10000])$] (**GC007_1**) Yuan
2. Increased ______ [hc$((0,\infty), int, -1)$, sc$((0,10000], int, -1)$, ub$([-1],[200,500,1000,5000,10000])$] (**GC007_2**) Yuan
3. Unaffected

GD.Self-Employed Activities

GD001 Did [XMainR]'s household members, including [XFLHHNameList], engage in any self-employed activities in the past year?

【**IWER**: In individual questionnaire, we ask question about self-employed activities for R. In household questionnaire, we ask question about self-employed activities for all household members.】

1. Yes
2. No

GD002 How many types of activities did [XMainR]'s household members, including [XFLHHNameList], participate in the past year? ______ [hc$([1,25], int, \varnothing)$] items

GD003 Who engaged in [GD002] items' self-employed activities in the past year? (select all that apply)

1-25. [XHHMemberName[i]]

26. [XMainR]

27. [XMainRS]

GD004 What is your best estimate of the net income earned from [GD002] items' self-employed activities in the past year? If the activity was conducted jointly with non-household

members, report only the net income earned by household members. Remember to consider the following types of costs: depreciation of fixed capital, energy, housing or equipment rental, raw materials, transportation, marketing, wages, taxes or fees. Do not include fixed capital investments in the past year.

【IWER: Please enter “-1” if R cannot answer.】

1. Earn money, net income ______ [hc$((0,\infty), int, -1)$, sc$((0,500000], int, -1)$, ub$([-1],[5000, 10000, 50000, 100000, 200000])$] (**GD004_1**) Yuan
2. Loss money, net income ______ [hc$((0,\infty), int, -1)$, sc$((0,500000], int, -1)$, ub$([-1],[5000, 10000, 50000, 100000, 200000])$] (**GD004_2**) Yuan
3. Break-even

999. Refuse

GD005 Without COVID-19 pandemic, [XMainR]’s household members, including [XFLHHNameList], may/may not have incomes from self-employed activities. After breakout of COVID-19, thing may change. From Chinese New year to now, the self-employed activity net incomes of [XMainR]’s household are increased, decreased or unaffacted because of COVID-19 pandemic?

【IWER: Please enter “-1” if R cannot answer.】

1. Decreased ______ [hc$((0,\infty), int, -1)$, sc$((0,50000], int, -1)$, ub$([-1],[500, 1000, 5000, 10000, 20000])$] (**GD005_1**) Yuan
2. Increased ______ [hc$((0,\infty), int, -1)$, sc$((0,50000], int, -1)$, ub$([-1],[500, 1000, 5000, 10000, 20000])$] (**GD005_2**) Yuan
3. Unaffected

GD006 Because of COVID-19 pandemic, has there been any change in the operating conditions of self-employed activities of [XMainR]’s household?

1. Do not have self-employed activity before and after COVID-19 pandemic
2. Have self-employed activity before and after COVID-19 pandemic
3. Do not have self-employed activity before COVID-19 pandemic and have after
4. Have self-employed activity before COVID-19 pandemic and do not have after

GD007 Because of COVID-19 pandemic, has [XMainR]’s household received any subsidy for self-employed activities from Chinese New year to now?

【IWER: Please enter “-1” if R cannot answer.】

1. Government load, borrow ______ [hc$((0,\infty), int, -1)$, sc$((0,500000], int, -1)$] (**GD007_1**) Yuan, interest rate ______ [hc$([0,100], real, -1)$] (**GD007_2**) % annual
2. Rent relief, reduce ______ [hc$((0,\infty), int, -1)$, sc$((0,50000], int, -1)$] (**GD007_3**) Yuan
3. Tax deduction, reduce ______ [hc$((0,\infty), int, -1)$, sc$((0,50000], int, -1)$] (**GD007_4**) Yuan
4. Other subsidies, please specify ______ (**GD007_5**) , reduce or receive ______ [hc$((0,\infty), int, -1)$, sc$((0,50000], int, -1)$] (**GD007_6**) Yuan
5. None of Above

[conflict$(5,[5]^c)$]

GE. Household Public Transfer Income

GE001 Did/Does [XMainR]'s household or [XMainR]'s household members, including [XFLHHNameList], received Wubao, Dibao, Tekun or poor household subsidies? (select all that apply)

1. Wubao subsidy
2. Dibao subsidy
3. Tekun subsidy
4. Jiandanglika poor household subsidy
5. Other poor household subsidy, please specify ______ (**GE001_1**)
6. None of Above

[conflict$(6, [6]^c)$]

GE002[i] Was [XMainR]'s household or household members once [XPoorHHName[i]] or is it now? From which year? If there is an interruption in the middle, it will be counted from the farthest time.

1. Now, begin time is ______ [hc$((1980, 2020], int, \varnothing)$] (**GE002_1[i]**) Year
2. Used to be, begin time is ______ [hc$((1980, 2020], int, \varnothing)$] (**GE002_2[i]**) Year

GE003[i] Who receive [XPoorHHName[i]]? If the subsidy was received jointly with non-household members, report only the household members.

1-25. [XHHMemberName[i]]
26. [XMainR]
27. [XMainRS]

GE004[i] How much did [XMainR]'s household members, including [XFLHHNameList], receive [XPoorHHName[i]]? If the subsidy was received jointly with non-household members, report only the amount received by household members. In kind should be converted into cash. ______ [hc$([0, \infty), int, -1)$, sc$((0, 20000], int, -1)$, ub$([-1], [500, 1000, 5000, 10000, 20000])$] Yuan

【**IWER**: Please enter "-1" if R cannot answer.】

GE006 Did [XMainR]'s household receive any of the following government subsidies, Social donations or compensations in the past year? (select all that apply) Excluding Wubao, Dibao, Tekun or poor household subsidies. COVID-19 pandemic related subsidies for household should be included in here. In kind should be converted into cash

【**IWER**: Please enter "-1" if R cannot answer.】

1. Reforestation: how much? ______ [hc$((0, \infty), int, -1)$, sc$((0, 20000], int, -1)$] (**GE006_1**) Yuan
2. Agricultural subsidies: how much? ______ [hc$((0, \infty), int, -1)$, sc$((0, 20000], int, -1)$] (**GE006_2**) Yuan
3. Work injury subsidies to the immediate family members: how much? ______ [hc$((0, \infty), int, -1)$, sc$((0, 20000], int, -1)$] (**GE006_3**) Yuan
4. Emergency or disaster relief (jiujikuan, jiuzaikuan) last year: how much? ______ [hc$((0, \infty), int, -1)$, sc$((0, 20000], int, -1)$] (**GE006_4**) Yuan
5. Social donations and subsidies, including food, clothes, school bags, crowdfunding medical expenses, and physical assistance: how much? ______ [hc$((0, \infty), int, -$

$1)$, $\texttt{sc}((0,20000], int, -1)$] (**GE006_5**) Yuan

6. Compensation for land seizure last: how much? ______ [$\texttt{hc}((0,\infty), int, -1)$, $\texttt{sc}((0,20000], int, -1)$] (**GE006_6**) Yuan
7. Compensation to pulling down your house or apartment last year: how much? ______ [$\texttt{hc}((0,\infty), int, -1)$, $\texttt{sc}((0,20000], int, -1)$] (**GE006_7**) Yuan
8. Other, please specify ______ (**GE006_8_1**): how much? ______ [$\texttt{hc}((0,\infty), int, -1)$, $\texttt{sc}((0,20000], int, -1)$] (**GE006_8**) Yuan
9. None

[$\texttt{conflict}(9,[9]^c)$]

GE007 How much did [XMainR]'s household receive any claims from productive insurance in past year? How much? (input 0 if not received). Productive insurance include agricultural insurance claims, etc. ______ [$\texttt{hc}([0,\infty), int, -1)$, $\texttt{sc}([0,20000], int, -1)$] Yuan

【**IWER**: Please enter "-1" if R cannot answer.】

【**INTRO**: We ask the public transfers received by the households (with household as the unit) and household members. Public transfers have characteristic of welfare, such as Wubao Subsidy and Tekun Subsidy given by government.】

GE008 Did [XMainR]'s household or household members, including [XFLHHNameList], receive any COVID-19 pandemic subsidies? Including COVID-19 pandemic subsidies for agricultural but not for self-employed activities. In kind should be converted into cash.

【**IWER**: Please enter "-1" if R cannot answer.】

1. Yes, total amount ______ [$\texttt{hc}((0,\infty), int, -1)$, $\texttt{sc}((0,20000], int, -1)$, $\texttt{ub}([-1],[500,1000,5000,10000,20000])$] (**GE008_1**) Yuan
2. No

GE009 Has [XMainR]'s household engaged in photovoltaic power generation to generate electricity for home use and national grid?

1. Yes
2. No

GE010 When did [XMainR]'s household install solar panels? ______ [$\texttt{hc}((1990,2020], int, -1)$] Year

【**IWER**: Please enter "-1" if R cannot answer.】

GE011 How much did [XMainR]'s household earn from photovoltaic power generation in the past year? Including values of home use and selling to national grid. ______ [$\texttt{hc}([0,\infty), int, -1)$, $\texttt{sc}((0,20000], int, -1)$] Yuan

【**IWER**: Please enter "-1" if R cannot answer.】

GE012 Does [XMainR]'s household have any collective distributing land? Did [XMainR]'s household rent out any of land in the past year? If rent out, How much rental income earned in the past year? If [XMainR]'s household does not have any land or did not rent out, please enter "0". ______ [$\texttt{hc}([0,\infty), int, -1)$, $\texttt{sc}([0,20000], int, -1)$, $\texttt{ub}([-1],[500,1000,5000,10000,20000])$] Yuan

【**IWER**: Please enter "-1" if R cannot answer.】

GE013 Does [XFamilyRAndS] have any house property? Did [XFamilyRAndS] rent out any of

house in the past year? If rent out, how much rental income earned in the past year? If [XFamilyRAndS] does not have any house or did not rent out, please enter "0". If there are many owners, please calculate the rental income of [XFamilyRAndS] based on property right. ______ [hc($[0, \infty), int, -1$), sc($[0, 20000], int, -1$), ub($[-1], [500, 1000, 5000, 10000, 20000]$)] Yuan

【**IWER**: Please enter "-1" if R cannot answer.】

GE014 How much rental income did [XMainR]'s household earn for any other household assets other than housing or land, such as trees, use of fixed capital, durables, or livestock?

【**IWER**: Please enter "-1" if R cannot answer.】

1. Ye, rental income ______ [hc($[0, \infty), int, -1$), sc($(0, 20000], int, -1$)] (**GE014_1**) Yuan
2. Not applicable

997. Don't know
999. Refuse

GE015[i] You said that [XMainR]'s household or household members was [XPoorHHName[i]]. The beginning time is [GE002_2[i]]. What is the end time? ______ [hc([GE002_2[i], 2020], $int, \varnothing$)] Year

GF.Household Living Expenditure

【**INTRO**: Please read aloud "This section is about your household living expenditure, including all your household members, [XFLHHNameList]".】

GF001 Generally, how much does [XMainR]'s household members, including [XFLHHNameList], spend a month? Including rent, food, clothing, communication expenses, water and electricity costs, fuel costs, service expenditures, entertainment expenditures, daily necessities and medical. ______ [hc($(0, \infty), int, \varnothing$), sc($(0, 20000], int, \varnothing$)] Yuan

GF002 We wish to know [XMainR]'s household food expenditure for the last week. Are you the primary person who purchases food for the household?

1. Yes
2. No

GF003 Who is the primary person purchasing food for [XMainR]'s household?

1-25. [XChildAliveName[i]]
26-35. [XHHOtherMemberName2[i]]
36. [XMainR]
37. [XMainRS]
38. Nanny
39. Neighbor
40. Other, please specify ______ (**GF003_1**)

【**IWER**: If possible, the primary person who purchases food for the household should answer the questions about expenditures GF004-GF010.】

GF004 In the past week, how many people usually ate meals together in your household, not including guests? ______ [hc$([0,\infty), int, \emptyset)$, sc$([1,10], int, \emptyset)$] Persons

GF005 Last week how many meals did [XMainR]'s household provide to guests? ______ [hc$([0,\infty), int, \emptyset)$, sc$([0,100], int, \emptyset)$] Meals

【**INTRO**: The next questions are about [XMainR]'s household living expenditure for all household members, including the expenditure happened outside, such as accommodation and meals fees.】

GF006 In the past week, how much did [XMainR]'s household spend on food, excluding banquet, wedding, eating out expenditure, alcohol, cigarettes, cigars and tobacco expenditure? ______ [hc$([0,\infty), int, \emptyset)$, sc$((0,6000], int, \emptyset)$] Yuan

GF007 Does [XMainR]'s household produce agricultural products, including plants, meat, eggs, aquatic lives, oil, vegetables and fruits, cigarettes and wine, drinks and milk products, produced food, seasonings, etc?

1. Yes
2. No

GF008 In the past week, what was the market value of the food that [XMainR]'s household members consumed that they grew? ______ [hc$([0,\infty), int, \emptyset)$, sc$((0,6000], int, \emptyset)$] Yuan

GF009 In the past week, how much did [XMainR]'s household members, including [XFLHHNameList], spend on eating out, excluding banquet and wedding? ______ [hc$([0,\infty), int, \emptyset)$, sc$([0,3000], int, \emptyset)$] Yuan

GF010 In the past week, how much did [XMainR]'s household members, including [XFLHHNameList], spend on alcohol, cigarettes, cigars and tobacc? ______ [hc$([0,\infty), int, \emptyset)$, sc$([0,3000], int, \emptyset)$] Yuan

GF011 Please tell me the expenditure on the following items last month for [XMainR]'s household, including [XFLHHNameList]

1.Communication fees, including post, internet usage, telephone and cell phone usage ______ [hc$([0,\infty), int, -1)$, sc$((0,5000], int, -1)$] (**GF011_1**) Yuan

2.Utilities: Water and electricity ______ [hc$([0,\infty), int, -1)$, sc$((0,5000], int, -1)$] (**GF011_2**) Yuan

3.Fuels, including gas, coal, etc. ______ [hc$([0,\infty), int, -1)$, sc$((0,5000], int, -1)$] (**GF011_3**) Yuan

4.Expenses for babysitters, housekeepers and servants ______ [hc$([0,\infty), int, -1)$, sc$((0,5000], int, -1)$] (**GF011_4**) Yuan

5.Local Transportation ______ [hc$([0,\infty), int, -1)$, sc$((0,5000], int, -1)$] (**GF011_5**) Yuan

6.Daily necessities, including toiletries, household items, kitchen supplies, decorative items, etc. ______ [hc$([0,\infty), int, -1)$, sc$((0,5000], int, -1)$] (**GF011_6**) Yuan

7.Entertainment, including expenses for books, newspapers, VCCs, DVDs, cinema tickets and bars ______ [hc$([0,\infty), int, -1)$, sc$((0,5000], int, -1)$] (**GF011_7**) Yuan

【**IWER**: Enter “0” if no corresponding expenditure; ask R to estimate a number if R can not remember clearly; enter “-1” if R cannot answer.】

GF012 Will the babysitters/hourly worker/servant employed by [XMainR]'s household take care of the following people? (select all that apply)

1. The elders in the household
2. The children in the household
3. Others, please specify ______ (**GF012_1**)
4. None of the above

[conflict(4, $[4]^c$)]

GF013 Please tell me the expenditure on the following items in the past year for [XMainR]'s household, including [XFLHHNameList].

1.Clothing and bedding ______ [hc($[0,\infty), int, -1$), sc($[0,100000], int, -1$)] (**GF013_1**) Yuan

2.Long distance traveling expenses, including expenses for traveling to and from home for work away, travel fares and hotel fees______ [hc($[0,\infty), int, -1$), sc($[0,100000], int, -1$)] (**GF013_2**) Yuan

3.Heating(centrally heated) ______ [hc($[0,\infty), int, -1$), sc($[0,100000], int, -1$)] (**GF013_3**) Yuan

4.Purchase, maintenance and repair costs of furniture, durable goods and electronics, includes refrigerator, washing machine, TV, computers and expensive instruments like pianio ______ [hc($[0,\infty), int, -1$), sc($[0,100000], int, -1$)] (**GF013_4**) Yuan

5.Education and training, including tuition, training fees, etc. ______ [hc($[0,\infty), int, -1$), sc($[0,100000], int, -1$)] (**GF013_5**) Yuan

6.Direct and indirect medical expenses. Note: Indirect medical expenses refer to transportation expenses, nutrition expenses, family expenses, etc. incurred due to medical treatment. It does not include the part already paid by Medicare ______ [hc($[0,\infty), int, -1$), sc($[0,100000], int, -1$)] (**GF013_6**) Yuan

7.Fitness expenditures, including fitness exercise and fitness equipment, health supplements ______ [hc($[0,\infty), int, -1$), sc($[0,100000], int, -1$)] (**GF013_7**) Yuan

8.Beauty, including make-ups, facials, massages, etc. ______ [hc($[0,\infty), int, -1$), sc($[0,100000], int, -1$)] (**GF013_8**) Yuan

9.Purchase, maintenance and repair costs of automobiles ______ [hc($[0,\infty), int, -1$), sc($[0,100000], int, -1$)] (**GF013_9**) Yuan

10.Purchase, maintenance and repair costs of transportation vehicles, appliances, communication products, etc.______ [hc($[0,\infty), int, -1$), sc($[0,100000], int, -1$)] (**GF013_10**) Yuan

11.Property management fees, including parking fee ______ [hc($[0,\infty), int, -1$), sc($[0,100000], int, -1$)] (**GF013_11**) Yuan

12.Taxes and fees turned over to the government, excluding income tax ______ [hc($[0,\infty), int, -1$), sc($[0,100000], int, -1$)] (**GF013_12**) Yuan

13.Donations to the society, including cash, and items like food, clothing, etc. ______ [hc($[0,\infty), int, -1$), sc($[0,100000], int, -1$)] (**GF013_13**) Yuan

14.The rent of the house or bed, including the accommodation expenses for household members, such as the campus dormitory fee, excluding the hotel fee for tourism ______ [hc($[0,\infty), int, -1$), sc($[0,100000], int, -1$)] (**GF013_14**) Yuan

15.Expenses for setting and holding banquets ______ [hc($[0,\infty), int, -1$), sc($[0,100000], int, -1$)] (**GF013_15**) Yuan

16.COVID-19 pandemic prevention spending, including masks, gowns and sanitizers ______ [hc($[0,\infty), int, -1$), sc($[0,100000], int, -1$)] (**GF013_16**) Yuan

【**IWER**: Enter "0" if no corresponding expenditure; ask R to estimate a number if R can not remem-

ber clearly; enter "-1" if R cannot answer.】

GF014 From the breakout of COVID-19 to now, can the income of [XMainR]'s household cover daily expenses?

1. Very difficult
2. Difficult
3. Easy
4. Very easy

GF015 From the breakout of COVID-19 to now, has [XMainR]'s household stopped paying rent, mortgage or utilities? (select all that apply)

1. Stop paying rent
2. Stop paying mortgage
3. Stop paying utilities
4. None of the above

[conflict$(4, [4]^c)$]

GF016 Since income cannot afford daily expenses, how did [XMainR]'s household pull through this hard time? The amounts of money in the following are from the breakout of COVID-19 to now. (select all that apply)

【**IWER**: Please enter "-1" if R cannot answer.】

1. Reduce spending, the amount reduced is ______ [hc$((0,\infty), int, -1)$, sc$((0, 50000], int, -1)$] (**GF016_1**) Yuan
2. Use savings, the amount is ______ [hc$((0,\infty), int, -1)$, sc$((0, 50000], int, -1)$] (**GF016_2**) Yuan
3. Sale of assets, such as ______ (**GF016_9**) , get ______ [hc$((0,\infty), int, -1)$, sc$((0, 50000], int, -1)$] (**GF016_3**) Yuan
4. Help from relatives and friends, the amount is ______ [hc$((0,\infty), int, -1)$, sc$((0, 50000], int, -1)$] (**GF016_4**) Yuan
5. Borrow money from relatives and friends, the amount is ______ [hc$((0,\infty), int, -1)$, sc$((0, 50000], int, -1)$] (**GF016_5**) Yuan
6. Borrow money from banks, the amount borrowed is ______ [hc$((0,\infty), int, -1)$, sc$((0, 50000], int, -1)$] (**GF016_6**) Yuan
7. Others, such as ______ (**GF016_8**), get ______ [hc$((0,\infty), int, -1)$, sc$((0, 50000], int, -1)$] (**GF016_7**) Yuan
8. Do nothing

[conflict$(8, [8]^c)$]

GF017 How often did the respondent receive assistance in answering section Household income and expenditure?

1. Never
2. A few times
3. Most or all of the time

I.Housing Characteristics

I001 What type of structure is [XFinancialResp]'s housing?

【**IWER**: Record if interviewer is in R's housing and clearly know the answer, otherwise, ask R.】

1. Concrete and steel/Bricks and wood
2. Adobe
3. Wood/Thatched
4. Cave dwelling
5. Mongolian yurt/Woolen felt/Tent
6. Stone
7. Other, please specify ______ (**I001_1**)

I002 When did [XFinancialResp] move into this housing? ______ [hc$([1900, 2020], int, -1)$] Year

【**IWER**: Please enter “-1” if R cannot answer.】

I003 If respondent is unclear about year, please choose among following items:

1. 0-5 years
2. 5-10 years
3. 10-20 years
4. 20-30 years
5. 30-40 years
6. More than 40 years

I004 Is [XFinancialResp]'s housing one story or multi-level building?

【**IWER**: Record if interviewer is in R's housing and clearly know the answer, otherwise, ask R.】

1. One-story building
2. Common multi-story building
3. Self-contained multi-story building

I005 Is the story independent or compound?

【**IWER**: Record if interviewer is in R's housing and clearly know the answer, otherwise, ask R.】

1. Independent story
2. Compound

I006 Which storey is this building on?

【**IWER**: If in the second basement, please select option 2 and enter “2”. Record if interviewer is in R's housing and clearly know the answer, otherwise, ask R.】

1. On the ground ______ [hc$([1, \infty), int, \emptyset)$, sc$([1, 20], int, \emptyset)$] (**I006_1**) Storey
2. Underground ______ [hc$([1, \infty), int, \emptyset)$, sc$([1, 5], int, \emptyset)$] (**I006_2**) Storey

I007 Does it have elevator?

【**IWER**: Record if interviewer is in R's housing and clearly know the answer, otherwise, ask R.】

1. Yes
2. No

I008 Was the elevator built with the building or was it added later?

【**IWER**: Please enter “-1” if R cannot answer.】

1. The same time as building
2. Later added, time for later added ______ [hc$([1900, 2020], int, -1)$] (**I008_1**) Year
3. Don't know

I009 Are there any handicapped facilities (e.g., non-stair ramp)?

【**IWER**: Record if interviewer is in R's housing and clearly know the answer, otherwise ask R.】

1. Yes
2. No
3. No steps on the flat ground, do not need handicapped facilities

I010 How many steps had to be climbed to get to the main entrance of the household's flat?

______ [hc$([0, \infty), int, \emptyset)$, sc$((0, 30], int, \emptyset)$] Steps

【**IWER**: Record if interviewer is in R's housing and clearly know the answer, otherwise ask R.】

I011 The [XFinancialResp]'s house has ______ [hc$([0, \infty), int, \emptyset)$, sc$((0, 20], int, \emptyset)$] (**I011_1**) bedrooms ______ [hc$([0, \infty), int, \emptyset)$, sc$((0, 20], int, \emptyset)$] (**I011_2**) living rooms ______ [hc$([0, \infty), int, \emptyset)$, sc$((0, 20], int, \emptyset)$] (**I011_3**) toilets ______ [hc$([0, \infty), int, \emptyset)$, sc$((0, 20], int, \emptyset)$] (**I011_4**) kitchens ______ [hc$([0, \infty), int, \emptyset)$, sc$((0, 20], int, \emptyset)$] (**I011_5**) balcony.

【**IWER**: Record if interviewer is in R's housing and clearly know the answer, otherwise ask R.】

I012 How far is the nearest toilet to [XFinancialResp]'s house? ______ [hc$([0, \infty), int, \emptyset)$, sc$((0, 100], int, \emptyset)$] Meters

I013 What is the type of toilet? Without a seat or with a seat? If both has, please select toilet with a sea.

【**IWER**: Record if interviewer is in R's housing and clearly know the answer, otherwise ask R.】

1. Toilet without a seat
2. Toilet with a seat

I014 Is the toilet flushable?

【**IWER**: Record if interviewer is in R's housing and clearly know the answer, otherwise ask R.】

1. Yes
2. No

I015 Does [XFinancialResp]'s house have electricity?

【**IWER**: Record if interviewer is in R's housing and clearly know the answer, otherwise ask R.】

1. Yes
2. No

I016 Does [XFinancialResp]'s house have tap water?

【**IWER**: Record if interviewer is in R's housing and clearly know the answer, otherwise ask R.】

1. Yes
2. No

I017 Is there in-house shower or bath facility? What type?

1. Hot water provided
2. Water heater installed by the household
3. No

I018 Does [XFinancialResp]'s house have coal gas or natural gas supply?

1. Yes
2. No

I019 Does [XFinancialResp]'s house have heating?

1. Yes
2. No

I020 What is the main heating energy source?

1. Solar
2. Coal
3. Natural gas
4. Liquefied Petroleum Gas
5. Electric
6. Crop residue/Wood burning
7. Other, please specify ______ (**I020_1**)
8. No heating

I021 What is the main source of cooking fuel?

1. Coal
2. Natural gas
3. Marsh gas
4. Liquefied petroleum gas
5. Electric
6. Crop residue/Wood burning
7. Solar
8. Other, please specify ______ (**I021_1**)
9. No cooking

I022 Does [XFinancialResp]'s house have a telephone connection?

【**IWER**: Record if interviewer is in R's housing and clearly know the answer, otherwise ask R.】

1. Yes
2. No

I023 Does [XFinancialResp]'s house have broad-band internet connection?

【**IWER**: Record if interviewer is in R's housing and clearly know the answer, otherwise ask R.】

1. Yes
2. No

I024 Does [XFinancialResp]'s house have an air cleaner?

【**IWER**: Record if interviewer is in R's housing and clearly know the answer, otherwise ask R.】

1. Yes
2. No

I025 How clear and tidy is in this household?

【**IWER**: Interviewer records it.】

1. Excellent
2. Very clear
3. Clear
4. Fair
5. Poor
6. Not applicable

I026 How is the temperature in this household?

【**IWER**: Interviewer records it.】

1. Very hot
2. Hot
3. Bearable
4. Cold
5. Very cold
6. Not applicable

I027 How is the floor in this household?

【**IWER**: Record if interviewer is in R's housing and clearly know the answer, otherwise ask R.】

1. Flat coverd, such as carpet, wooden floor, marble, ceramic tile, floor leather
2. Flat concrete
3. Uneven

I028 What is the size of [XFinancialResp]'s house? ______ [hc($(0, \infty), real, \varnothing$), sc($(10, 500], real, \varnothing$)] square meter

I029 Is tape water treated by waterworks?

1. Yes
2. No

997. Don't know

G2 Individual Income

G2.Proxy Mode Confirmation

proxy_8 Interviewer record: Enter proxy mode?

1. Yes
2. No

GA.Individual Income

【**IWER**: This section is asked of the main R and spouse respectively. Do not allow a proxy R to answer the entire section.】

GA001 In the past year, did [XRName] receive any wage and bonus income, excluding retirement, receding and internal retirement income? Here wage is from all employments.

1. Yes
2. No

GA002 How much did [XRName] receive last year? ______ [hc($(0, \infty), int, -1$), sc($(0, 240000], int, -1$), ub($[-1], [5000, 10000, 30000, 50000, 100000]$)] Yuan

【**IWER**: Please enter "-1" if R cannot answer.】

GA003 Does the above mentioned wage exclude any insurance, income tax, public housing funds and other fees?

1. Yes
2. No
997. Don't know
999. Refuse

GA004 What is the total amount of [XRName] [XGA004Text]'s insurance, income tax, public housing funds and other fees?

【**IWER**: Please enter "-1" if R cannot answer.】

1. ______ [hc$((0,\infty),int,-1)$, sc$((0,10000],int,-1)$, ub$([-1],[300,500,1000,2000,3000])$] (**GA004_1**) Yuan/Month
2. ______ [hc$((0,\infty),int,\varnothing)$, sc$((0,100000],int,\varnothing)$] (**GA004_2**) Yuan/Year
3. About ______ [hc$((0,100],real,\varnothing)$] (**GA004_3**) % of wage
4. None, 0 Yuan

GA005 Did [XRName] receive any of the following types of individual transfer income in the past year? (select all that apply) Individual COVID-19 pandemic subsidies are also individual transfer incomes.

【**IWER**: Please enter "-1" if R cannot answer.】

1. Pensions, including pension incomes from governments, public institutions and firms, supplemental pension of the firms, receding and internal retirement compensation and incomes from such programs as rural pension insurance, Urban residents' pension, commercial pension insurance, new rural social pension insurance, life insurance and pension for land-expropriated farmers ______ [hc$((0,\infty),int,-1)$, sc$((0,100000],int,-1)$, ub$([-1],[1000,3000,6000,10000,25000])$] (**GA005_1**) Yuan
2. Unemployment compensation ______ [hc$((0,\infty),int,-1)$, sc$((0,50000],int,-1)$] (**GA005_2**) Yuan
3. Pension voucher ______ [hc$((0,\infty),int,-1)$, sc$((0,50000],int,-1)$] (**GA005_3**) Yuan
4. Pension subsidy for the oldest old ______ [hc$((0,\infty),int,-1)$, sc$((0,50000],int,-1)$] (**GA005_4**) Yuan
5. Workers' compensation from industrial accident compensation insurance includes wage-replacement benefits, disability benefits, and survivors' benefits ______ [hc$((0,\infty),int,-1)$, sc$((0,50000],int,-1)$] (**GA005_5**) Yuan
6. Elderly family planning subsidies ______ [hc$((0,\infty),int,-1)$, sc$((0,50000],int,-1)$] (**GA005_6**) Yuan
7. Medical aid ______ [hc$((0,\infty),int,-1)$, sc$((0,100000],int,-1)$] (**GA005_7**) Yuan
8. Other government subsidies, excluding Dibao, Wubao, tekun and poor subsidies, please specify ______ (**GA005_8_1**), ______ [hc$((0,\infty),int,-1)$, sc$((0,100000],int,-1)$] (**GA005_8**) Yuan
9. Other social income sources, please specify ______ (**GA005_9_1**) , ______ [hc$((0,\infty),int,-1)$, sc$((0,100000],int,-1)$] (**GA005_9**) Yuan
10. None of the above

[conflict$(10,[10]^c)$]

GA006 Without COVID-19 pandemic, [XRName] may/may not have wage. After breakout of COVID-19, thing may change. From Chinese New year to now, [XRName]'s wages are

increased, decreased or unaffacted because of COVID-19 pandemic?

【**IWER**: Please enter "-1" if R cannot answer.】

1. Decreased ______ [hc$((0,\infty), int, -1)$, sc$((0,50000], int, -1)$, ub$([-1],[1000,3000,5000,10000,20000])$] (**GA006_1**) Yuan
2. Increased ______ [hc$((0,\infty), int, -1)$, sc$((0,50000], int, -1)$, ub$([-1],[1000,3000,5000,10000,20000])$] (**GA006_2**) Yuan
3. Unaffected

GA007 Did [XRName] make a will?

1. Yes
2. No

GA008 Have [XRName] made estate planning?

1. Have considered
2. Have not considered
3. No asset

GA009 [XGA009Text] if [XRName] dies, what are the details of estate planning?

【**IWER**: Please enter "-1" if R cannot answer.】

1. Spouse ______ [hc$((0,100], real, -1)$] (**GA009_1**) %
2. Children/Sons-in-law/Daughters-in-law
3. Sibling ______ [hc$((0,100], real, -1)$] (**GA009_3**) %
4. Other Relatives ______ [hc$((0,100], real, -1)$] (**GA009_4**) %
5. Parents/Parents-in-law ______ [hc$((0,100], real, -1)$] (**GA009_5**) %
6. Grandchildren
7. Friends ______ [hc$((0,100], real, -1)$] (**GA009_7**) %
8. Charity ______ [hc$((0,100], real, -1)$] (**GA009_8**) %
9. Others, please specify ______ (**GA009_9_1**) , ______ [hc$((0,100], real, -1)$] (**GA009_9**) %
10. Do not distribute

997. [XProxyText]

[conflict$(10, 997, [10, 997]^c)$]

GA010 To which children is the estate distributed? Children's spouse's portion is counted as children. If children are not in the list, please add children's name. (select all that apply)

1-25. [XChildAliveName[i]]

26-35. Other child, name is ______ (**GA010_1[i]**)

GA011[i] [XGAChildList1[i]] is distributed ______ [hc$((0,100], real, -1)$] %.

【**IWER**: Please enter "-1" if R cannot answer.】

GA014 Grandchildren are which children's children? If Grandchildren's parents are not in the list, please add children's name. (select all that apply)

1-25. [XChildAliveName[i]]

26-35. Other child, name is ______ (**GA014_1[i]**)

GA015[i] [XGAChildList2[i]] 's children are distributed ______ [hc$((0,100], real, -1)$] %.

【**IWER**: Please enter "-1" if R cannot answer.】

GA016 What is the actual allocation time?

1. [XRName] died
2. [XRName] alive

997. [XProxyText]

[conflict(997, [997]^c)]

GA017 Does [XRName]'s estate planning follow law of succession?

1. Yes
2. No, I have my plan

GA018 What is the actual allocation time?

1. [XRName] and spouse both died
2. [XRName] and spouse both alive
3. [XRName] or spouse died

997. [XProxyText]

[conflict(997, [997]^c)]

Auxiliary Variable Definition

XFLHHFinancialNameList connect main respondent's name and spouse's name with "or"

```
if (!empty("XMainRS")) {
        add("XFLHHFinancialNameList", value("XMainR")+"or"+value("XMainRS"))
} else {
        add("XFLHHFinancialNameList", value("XMainR"))
}
```

XFinancialResp financial respondent's name

```
for (var i1 = 1; i1 < 26; i1++) {
  if (equal("GB001", i1)) {
^^I^^Iadd("XFinancialResp", value("XHHMemberName[i1]"))
^^I}
}
if (equal("GB001", 26)) {
^^Iadd("XFinancialResp", value("XMainR"))
}
if (equal("GB001", 27)) {
^^Iadd("XFinancialResp", value("XMainRS"))
}
```

XGB005Text text for GB005

```
add("XGB005Text[i]", "")
if (equal("GB004[i]", "2") || equal("GB004[i]", "997")) {
^^Iadd("XGB005Text[i]", "shall")
}
```

XFLHHNameList connect household members' name with ","

```
if (!empty("XMainRS")) {
        add("XFLHHNameList", value("XMainR")+","+value("XMainRS"))
} else {
        add("XFLHHNameList", value("XMainR"))
}

for (var i1 = 1; greater("XHHMemberNum", i1, true) ; i1++){
        add("XFLHHNameList", value("XFLHHNameList")+","+value("XHHMemberName[i1]"))
}
```

XHHOtherMemberName2 Names of household members who are not children

```
for (var k = 1; k <= 10; k++) {
    add("XHHOtherMemberName2["+(k+25)+"]", value("XHHOtherMemberName[k]"))
}
```

XPoorHHName Name of government subsidies

```
add("XPoorHHName[1]", "Wubao subsidy")
add("XPoorHHName[2]", "Dibao subsidy")
add("XPoorHHName[3]", "Tekun subsidy")
add("XPoorHHName[4]", "Jiandanglika subsidy")
add("XPoorHHName[5]", "Other poor household subsidy, "+value("GE001_1"))
```

XPoorLstYr Did household get government subsidy [i] in the past year? 0 No, 1 Yes

```
add("XPoorLstYr[i]", "0")
if (equal("GE002[i]", "1")) {
    add("XPoorLstYr[i]", "1")
}
if (equal("GE002[i]", "2") && equal("GE015[i]", "2020")) {
    add("XPoorLstYr[i]", "1")
}
```

XFLHHNameListPoor String of household members' names who get government subsidy [i] in the past year

```
add("XFLHHNameListPoor[i]", "")
for (var i1 = 1; i1 < 26; i1++) {
  ^^Iif (selected("GE003[i]", i1)) {
^^I^^Iadd("XFLHHNameListPoor[i]", value("XHHMemberName[i1]")+", "+value("XFLHHNameListPoor[i]"))
^^I}
}

if (selected("GE003[i]", 26)) {
^^I^^Iadd("XFLHHNameListPoor[i]", value("XMainR")+", "+value("XFLHHNameListPoor[i]"))^^I
}

if (selected("GE003[i]", 27)) {
^^I^^Iadd("XFLHHNameListPoor[i]", value("XMainRS")+", "+value("XFLHHNameListPoor[i]"))^^I
}
```

XProxyText In the proxy mode, add option "Don't know" in some questions

```
add("XProxyText", "")
if (equal("proxy_8", "1")) {
^^Iadd("XProxyText", "Don't know")
}
```

XGA004Text Text for GA004

```
add("XGA004Text", "")
if (equal("GA003", "2") || equal("GA003", "997")) {
^^Iadd("XGA004Text", "shall")
}
```

XGA009Text Text for GA009

```
add("XGA009Text", "")
if (equal("GA007", "1")) {
^^Iadd("XGA009Text", "in the will")
}
```

XGAChildList1 1st children list in the GA section

```
for (var i1 = 1; i1 <=25 ; i1++) {
    add("XGAChildList1[i1]", value("XChildAliveName[i1]"))
}
for (var i1 = 26; i1 <=35 ; i1++) {
    add("XGAChildList1[i1]", value("GA010_1[i1]"))
}
```

XGAChildList2 2nd children list in the GA section

```
for (var i1 = 1; i1 <=25 ; i1++) {
    add("XGAChildList2[i1]", value("XChildAliveName[i1]"))
}
for (var i1 = 26; i1 <=35 ; i1++) {
    add("XGAChildList2[i1]", value("GA014_1[i1]"))
}
```

V COVID

V. Proxy Mode Confirmation

proxy_14 IWER: Enter proxy mode?

1. Yes
2. No

VA. Awareness

【**INTRO**: I would like to know the impact of the coronavirus pandemic on [XRName].】

VA001 Is [XRName] aware of any of the following practices to reduce the risk of coronavirus infection? (Select All That Apply)

【**IWER**: The COVID module only covers information about coronavirus prevention and control. Please refer to the other modules for questions related to the impact of the pandemic on health-care utilization, employment and income.】

1. Wash hands
2. Use alcohol and disinfectants
3. Avoid shaking hand
4. Wear masks and gloves
5. Avoid travel
6. Avoid gatherings
7. Social distancing
8. Other, please describe briefly ______ (**VA001_1**)
9. [XRName] did not know about the pandemic or any preventive measures (EXCLUSIVE)
10. [XRName] knows about the pandemic but any preventive measures (EXCLUSIVE)
11. [XRName] did not know about the pandemic and any preventive measures (EXCLUSIVE)

[conflict$(9, 10, 11, [9, 10, 11]^c)$]

VA002 Where did [XRName] learn how to reduce the risk of infection? (Select All That Apply. Don't Read The Answers)

1. TV news
2. Newspaper
3. Online media such as Internet news and WeChat
4. Radio broadcast
5. Family, friends, colleagues and the community
6. Healthcare practitioner
7. Village broadcast
8. Poster
9. Other
10. No external source, all from [XRName]'s own understanding and life experience. (EXCLUSIVE)

[conflict$(10, [10]^c)$]

VA003 During the pandemic, did [XRName] wear a mask when going out?

1. Always
2. Sometimes
3. Never
4. Didn't go out during the pandemic

VA004 Does [XRName] go out now? Wear a mask when going out? (Don't Read The Answers)

1. Wears a mask when going out
2. Doesn't wear a mask when going out
3. Doesn't go out at all

VA005 Does [XRName] wear a mask when going out now? If did wear before, but not now, when did [XRName] start not wearing a mask? (Don't Read The Answers)

【**IWER**: If [XRName] now wears a mask sometimes, please select the first answer. If R cannot answer the month, enter "-1".】

1. Still wears when going out
2. Did wear during the pandemic, but not now. Hasn't wore for ______ [hc([0, 14], *int*, −1), sc([0, 6], *int*, −1)] (**VA005_1**) months
3. Never wears a mask
4. Hasn't gone out since the pandemic

VA006 During the three days after Wuhan was closed due to the pandemic, that is, from the Chinese New Year's Eve to the second day of the first Lunar Month (from January 24 to January 26), did [XRName] buy more of the following things than usual to stock up because of the pandemic? (Select All That Apply)

1. Food, cooking oil and vegetables
2. Face masks, hand sanitizer and disinfectant
3. None of the above

[conflict(3, $[3]^c$)]

VA007 During the pandemic, our government took some measures to contain the spread of coronavirus, do you think it is too strict, appropriate or not strict enough? (Don't Read The Answers)

1. Too strict
2. Appropriate
3. Not strict enough

997. Don't know
999. Refuse

VB. Infection and Quarantine

VB001 Has [XRName], his/her neighbor, or acquaintance ever been identified as a confirmed or suspected case of coronavirus? (Select All That Apply. Don't Read The Answers)

1. [XRName]'s self
2. Household member: ______ (**VB001_1**)
3. Other relative (not living in the same house): ______ (**VB001_2**)
4. Acquaintance (e.g., friends, neighbors, colleagues, etc.) : ______ (**VB001_3**)

5. None
999. Refuse
[conflict$(5, 999, [5, 999]^c)$]

VB002 Are they all recovering well? Has anyone passed away? If yes, what is his/her relationship with [XRName]? (Select All That Apply. Don't Read The Answers)
1. Household member: ______ (**VB002_1**)
2. Other relative (not living in the same house): ______ (**VB002_2**)
3. Acquaintance (e.g., friends, neighbors, colleagues, etc.): ______ (**VB002_3**)
4. None
999. Refuse
[conflict$(4, 999, [4, 999]^c)$]

VB004 Was [XRName] hospitalized for treatment of coronavirus? How many days did [XRName] stay in the hospital? ______ [hc$([0, 250], int, -1)$] days? (Enter "0" if not hospitalized)
【**IWER**: If R cannot answer, enter "-1".】

VB005 Has [XRName] ever been placed under quarantine or medical observation for any of the following reasons [XVAHospitalizationExcluded]? Home isolation and building closure are both considered quarantine. (Select All That Apply)
1. Travel (including return to work after the Chinese New Year)
2. Close contact of a coronavirus case
3. Apartment or residential building was closed (excluding closure of the residential area)
4. Asked to be quarantined when going to hospital or after being discharged
5. Nucleic acid test shows positive
6. No quarantine experience (EXCLUSIVE)
997. Don't know
999. Refuse
[conflict$(6, 997, 999, [6, 997, 999]^c)$]

VB008 [XRName] Total number of days in quarantine? ______ [hc$([1, 250], int, -1)$], sc$([1, 14], int, -1)$] days
【**IWER**: For multiple quarantine experiences, please count all the days. If R cannot answer, enter "-1".】

VB009 Where was the place of [XRName]'s quarantine? (Select All That Apply)
1. Hospital
2. A centralized medical observation site such as a hotel
3. Own residence
4. Other: ______ (**VB009_1**)
997. Don't know
999. Refuse
[conflict$(997, 999, [997, 999]^c)$]

VB010 How much did [XRName] pay for the quarantine? Including charges for the accommodation and food. ______ [hc$([0, 100000], int, -1)$] yuan
【**IWER**: If R cannot answer, enter "-1".】

VB011 During [XRName]'s quarantine experience, was there someone else quarantined in the same site whom [XRName] could see and talk to?

1. Yes
2. No

997. Don't know
999. Refuse

VB012 Has [XRName] ever been tested for the coronavirus?

【**IWER**: If R cannot answer the month, please enter "-1".】

1. Yes, last time tested in ______ [hc([2020,XIWYear], *int*, −1)] (**VB012_1**) Year ______ [hc([1, 12], *int*, −1), sc([1,XIWMonth], *int*, −1)] (**VB012_2**) Month
2. No

997. Don't know
999. Refuse

VC. Activities

VC000 During the pandemic [XVCNotInQuarantine], did [XRName] ever stay home for several days for fear of infection? What was the longest period of time this happened? ______ [hc([0, 250], *int*, −1)] days (If no such case or going out every day, enter "0".)

【**IWER**: If R cannot answer, enter "-1".】

【**INTRO**: I would like to know [XRName]'s activities during the outbreak of the pandemic, that is, [XVCOutbreak] after the Chinese New Year, from around January 25 to February 22.】

VC001 During [XVCOutbreak], where did [XRName] generally live?

【**IWER**: If R does not know the street district or community, select as appropriate】

1. The same village or community with the current residence: [XRResidenceFull]
2. The same county or district with the current residence, [XRResidenceCounty], but in other village or community: ______ (**VC001_1**) township/town/street district/village/community
3. Other county or district: ______ (**VC001_2**) province/city/county ______ (**VC001_3**) township/town/street district/village/community
4. Areas where none of the above options apply (Hong Kong, Macao, Taiwan, or abroad)

999. Refuse

VC002 During [XVCOutbreak] [XVCNotInQuarantine], did [XRName]'s frequency of going outdoors each day increase, decrease, or remain unchanged compared to what would have been if the pandemic had not happened?

【**IWER**: The situation when the pandemic had not happened, cannot be interpreted as the situation in the first Lunar month of last year or now in normal times.

SPECIAL CASE: If R does not know anything about the pandemic so that the situation when the pandemic did not happen cannot be understood, you can interpret "compared to the situation if the pandemic had not happened" as "compared to the situation in the first Lunar month of last year, given current health condition". The same applies below.】

1. Increased a lot

2. Increased a little
3. Unchanged
4. Decreased a little
5. Decreased a lot

VC003 During [XVCOutbreak] [XVCNotInQuarantine], did the number of hours [XRName] spent outdoors each day increase, decrease, or remain unchanged compared to what would have happened if the pandemic had not happened?

【**IWER**: The situation when the pandemic had not happened, cannot be interpreted as the situation in the first Lunar month of last year or now in normal times.】

1. Increased a lot
2. Increased a little
3. Unchanged
4. Decreased a little
5. Decreased a lot

VC004 During [XVCOutbreak] [XVCNotInQuarantine], did the amount of time [XRName] spent in vigorous activities each day increase, decrease, or remain unchanged compared to what would have been if the pandemic had not happened? Vigorous activities are very physically demanding and can cause shortness of breath, such as heavy lifting, digging, plowing, aerobic exercise, fast cycling, and cycling with cargo.

【**IWER**: The situation when the pandemic had not happened, cannot be interpreted as the situation in the first Lunar month of last year or now in normal times.】

1. Increased a lot
2. Increased a little
3. Unchanged
4. Decreased a little
5. Decreased a lot

VC005 During [XVCOutbreak] [XVCNotInQuarantine], did the amount of time [XRName] spent in moderate activities each day increase, decrease, or remain unchanged compared to what would have been if the pandemic had not happened? Moderate activities include carrying light loads, mopping, biking at regular speed, and speed walking, etc.

【**IWER**: The situation when the pandemic had not happened, cannot be interpreted as the situation in the first Lunar month of last year or now in normal times.】

1. Increased a lot
2. Increased a little
3. Unchanged
4. Decreased a little
5. Decreased a lot

VC006 During [XVCOutbreak] [XVCNotInQuarantine], did the amount of time [XRName] spent in light activities such as walking and strolling each day increase, decrease, or remain unchanged compared to what would have been if the pandemic had not happened?

【**IWER**: The situation when the pandemic had not happened, cannot be interpreted as the situation in the first Lunar month of last year or now in normal times.】

1. Increased a lot

2. Increased a little
3. Unchanged
4. Decreased a little
5. Decreased a lot

VC007 During [XVCOutbreak] [XVCNotInQuarantine], did the frequency of [XRName] visiting neighbors increase, decrease, or remain unchanged compared to what would have been if the pandemic had not happened?

【**IWER**: The situation when the pandemic had not happened, cannot be interpreted as the situation in the first Lunar month of last year or now in normal times.】

1. Increased a lot
2. Increased a little
3. Unchanged
4. Decreased a little
5. Decreased a lot
6. Never done this

VC008 During [XVCOutbreak] [XVCNotInQuarantine], did the frequency of [XRName] playing mahjong, chess, and cards increase, decrease, or remain unchanged compared to what would have been if the pandemic had not happened?

【**IWER**: The situation when the pandemic had not happened, cannot be interpreted as the situation in the first Lunar month of last year or now in normal times.】

1. Increased a lot
2. Increased a little
3. Unchanged
4. Decreased a little
5. Decreased a lot
6. Never done this

VC009 During [XVCOutbreak] [XVCNotInQuarantine], did the frequency of [XRName] participating in square dancing increase, decrease, or remain unchanged compared to what would have been if the pandemic had not happened?

【**IWER**: The situation when the pandemic had not happened, cannot be interpreted as the situation in the first Lunar month of last year or now in normal times.】

1. Increased a lot
2. Increased a little
3. Unchanged
4. Decreased a little
5. Decreased a lot
6. Never done this

VC010 During [XVCOutbreak], did the frequency of [XRName] making phone calls or sending text messages increase, decrease, or remain unchanged compared to what would have been if the pandemic had not happened?

【**IWER**: The situation when the pandemic had not happened, cannot be interpreted as the situation in the first Lunar month of last year or now in normal times.】

1. Increased a lot

2. Increased a little
3. Unchanged
4. Decreased a little
5. Decreased a lot
6. No such devices in then residence
7. Never done this

VC011 During [XVCOutbreak], did the frequency of [XRName] using the Internet to communicate with friends and relatives, such as Wechat messaging and voice/video chat, increase, decrease, or remain unchanged compared to what would have been if the pandemic had not happened?

【**IWER**: The situation when the pandemic had not happened, cannot be interpreted as the situation in the first Lunar month of last year or now in normal times.】

1. Increased a lot
2. Increased a little
3. Unchanged
4. Decreased a little
5. Decreased a lot
6. No such devices in then residence
7. Never done this

VC012 During [XVCOutbreak], has [XRName] ever felt the fear of the pandemic or any fear related to the pandemic?

【**IWER**: The situation when the pandemic had not happened, cannot be interpreted as the situation in the first Lunar month of last year or now in normal times.】

1. Rarely or not at all
2. Not much
3. Sometimes or half of the time
4. Most of the time

997. Don't know
999. Refuse

VC013 During [XVCOutbreak], has [XRName] ever felt stressful or anxious about the pandemic or anything related to the pandemic?

【**IWER**: The situation when the pandemic had not happened, cannot be interpreted as the situation in the first Lunar month of last year or now in normal times.】

1. Rarely or not at all
2. Not much
3. Sometimes or half of the time
4. Most of the time

997. Don't know
999. Refuse

VC014 During [XVCOutbreak], did the amount of [XRName]'s smoking increase, decrease, or remaine unchanged compared to what would have been if the pandemic had not happened?

【**IWER**: "Never smoked" can mean having not smoked in recent years. The situation when the pan-

demic had not happened, cannot be interpreted as the situation in the first Lunar month of last year or now in normal times.】

1. Increased a lot
2. Increased a little
3. Unchanged
4. Decreased a little
5. Decreased a lot
6. Never smoked before

VC015 During [XVCOutbreak], did the amount of [XRName]'s alcohol drinking increase, decrease, or remaine unchanged compared to what would have been if the pandemic had not happened?

【**IWER**: "Never drank" can mean having not drank in recent years. The situation when the pandemic had not happened, cannot be interpreted as the situation in the first Lunar month of last year or now in normal times.】

1. Increased a lot
2. Increased a little
3. Unchanged
4. Decreased a little
5. Decreased a lot
6. Never drank before

VC016 During [XVCOutbreak], did the number of hours of [XRName] sleeping each night increase, decrease, or remain unchanged compared to what would have been if the pandemic had not happened? Sleep time refers to the time spent actually asleep, which may be shorter than the time spent lying in bed.

【**IWER**: The situation when the pandemic had not happened, cannot be interpreted as the situation in the first Lunar month of last year or now in normal times.】

1. Increased a lot
2. Increased a little
3. Unchanged
4. Decreased a little
5. Decreased a lot

VC017 During [XVCOutbreak], did the amount of food eaten by [XRName] each day increase, decrease, or remain unchanged compared to what would have been if the pandemic had not happened?

【**IWER**: The situation when the pandemic had not happened, cannot be interpreted as the situation in the first Lunar month of last year or now in normal times.】

1. Increased a lot
2. Increased a little
3. Unchanged
4. Decreased a little
5. Decreased a lot

VD. Restrictions in the Residence Area

VD001 I would like to learn from you whether the following types of restrictions on internal and external access have been imposed in the neighborhoods or villages where [XRName] has lived since the Chinese New Year due to the pandemic control? How many days did each of the restrictions last? The four types of restrictions are listed in the order of from tightest to loosest. The periods of restriction should not overlap, and the length of time combined should not exceed [XIWMonth] months. (Select All That Apply)

【**IWER**: Only restrictions experienced by [XRName] should be considered. The period of being restricted may be discontinuous due to residence changing or recurring pandemic outbreaks, and the discontinuous periods related to the same restriction need to be summed together.

Entry/exit passes and face recognition, means of identifying interior residents, are restrictions on visitors, not on residents.

If a restriction experience has not yet ended, the length of time ends on the day of this visit. You can ask for approximate start and end dates to assist R in the calculation.

If R cannot answer the number of restriction days, enter "-1".】

1. Closed to interior residents and visitors. Total days of restriction ______ [hc$([1,120], int, -1)$] (**VD001_1**) days
2. Semi-closed to interior residents, and no visitors are allowed, for example, daily pass for grocery shopping and essential work is required. Total days of restriction ______ [hc$([1,200], int, -1)$] (**VD001_2**) days
3. Entry and exit of interior residents are not restricted, and no visitors are allowed. Total days of restriction ______ [hc$([1,300], int, -1)$] (**VD001_3**) days
4. Entry and exit of interior residents are not restricted, and visitors are allowed under certain conditions, for example, green health codes. Total days of restriction ______ [hc$([1,400], int, -1)$, sc$([1,300], int, -1)$] (**VD001_4**) days
5. None of the above restriction types were experienced, and access to the residential area was the same as before the pandemic. (EXCLUSIVE)

997. Don't know about the above four types of control measures
999. Refuse

[conflict$(5, 997, 999, [5, 997, 999]^c)$]

VD002 I would like to learn from you whether square dancing activities have ever been cancelled in [XRName]'s residence since the Chinese New Year? Including all the cancellations, what is the total number of days until today? ______ [hc$([0,250], int, -1)$] days (Fill in "0" if not experienced)

【**IWER**: If R cannot answer, enter "-1".】

VD003 I would like to learn from you whether mahjong houses or community rooms playing chess and cards have ever been closed in [XRName]'s residence since the Chinese New Year? Including all the closures, what is the total number of days until today? ______ [hc$([0,250], int, -1)$] days (Enter "0" if not experienced)

【**IWER**: If R cannot answer, enter "-1".】

Auxiliary Variable Definition

XQuarantined Whether quarantined

```
if (selected("VB005", "1") || selected("VB005", "2") || selected("VB005", "3") || selected("VB005", "4")
↪  || selected("VB005", "5")) {
    add("XQuarantined", "1")
} else {
    add("XQuarantined", "0")
}
```

XVCOutbreak WORDING: the period of the coronavirus outbreak

```
add("XVCOutbreak", "the first Lunar month")
```

XVCNotInQuarantine WORDING: excluding quarantine

```
if (equal("XQuarantined", "1")) {
    add("XVCNotInQuarantine", ", excluding quarantine")
}
```

XVAHospitalizationExcluded WORDING: excluding hospitalization

```
if (greater("VB004", "0")) {
    add("XVAHospitalizationExcluded", ", if not considering hospitalization time")
}
```

EX Exit

EXB. Demographics

【**INTRO**: Sorry, the previous information is incomplete. I need to confirm with you again.】

EXB001 What was the date on which [XRName] died? ______ [hc([2011, 2020], *int*, ∅), sc([ZIWYear, 2020], *int*, ∅)] (**EXB001_1**) Year ______ [hc([1, 12], *int*, ∅)] (**EXB001_2**) Month ______ [hc([1, 31], *int*, ∅)] (**EXB001_3**) Day

【**IWER**: Mark the year using four digits.】

EXB002 Is the date of death based on the solar or the lunar calendar?

1. Solar calendar
2. Lunar calendar

EXB003 [XRName]'s residence address, before his/her death?

1. Chinese mainland ______ (**EXB003_1**) province/city/county/district ______ (**EXB003_2**) township village/neighborhood ______ (**EXB003_3**) building number
2. Hong Kong, China
3. Macao, China
4. Taiwan, China
5. Abroad: ______ (**EXB003_4**)

EXB004 What's the type of [XRName]'s address before his/her death?

1. Family housing
2. Workplace
3. Nursing home
4. Hospital
5. Other, please specify ______ (**EXB004_1**)

EXB005 In what province and county did [XRName] died?

1. [XAliveResidenceFull]
2. Chinese mainland: ______ (**EXB005_1**) province/city/county/district ______ (**EXB005_2**) township village/neighborhood ______ (**EXB005_3**) building number
3. Hong Kong, China
4. Macao, China
5. Taiwan, China
6. Abroad: ______ (**EXB005_4**)

EXB006 At the time of death, was [XRName] at home, at workplace, in a hospital, in a nursing home, in a hospice, or what?

1. At home
2. At workplace
3. In hospital
4. On the way to the hospital
5. In nursing home
6. In hospice
7. Other, please specify ______ (**EXB006_1**)

EXB007 Is the death expected at about the time it occurred, or is it unexpected?

1. Expected
2. Unexpected
3. Other, please specify ______ (**EXB007_1**)

EXB008 About how long was it between the start of the final illness and the death: was it one or two hours, less than a day, less than a week, less than a month, less than a year, or was it more than a year?

1. One or two hours (or no warning)
2. Less than a day
3. Less than a week
4. Less than a month
5. Less than a year
6. More than a year: ______ [hc([1,XRExitAge], *real*, ∅), sc([1, 20], *real*, ∅)] (**EXB008_1**) Year

EXB009 At the time [XRName] died, what is the marital status?

1. Married
2. Separated
3. Divorced
4. Widowed
5. Never married
6. Cohabitation

EXB010 Did [XRName] lived with his/her spouse/cohabitant when he/she died?

1. Yes
2. No

EXB011 Did [XRName] have the death certificate?

1. Yes
2. No

EXB012 In what province and county did [XRName]'s death certificate was filed?

【**IWER**: Please find the medical certificate of death as far as possible.】

1. [XAliveResidenceFull]
2. [XAliveResidenceFull] ______ (**EXB012_1**)
3. Other: ______ (**EXB012_2**) province/city/county/district ______ (**EXB012_3**) township village/neighborhood
4. Abroad: ______ (**EXB012_4**)

EXB013 Has [XRName]'s household registration cancelled?

1. Yes
2. No

EXB014 What's the reason of not cancel?

1. Time is too short to deal with the cancellation
2. The cancellation is not important
3. Other, please specify ______ (**EXB014_1**)

VA1A730 What was [XRName]'s ID card number of deceased before His/Her death? ______

【**IWER**: There is ID card number information on the death medical certificate.】

VA1A750 The cause of death on the death certificate: ______
Photos of medical certificate of death: ______ (**VA1A750_1**)
【**IWER**:Please explain to the respondent that the data is only used for scientific research and will not disclose any personal privacy.】

EXC. Family

【**INTRO**: In the following section, we would like to ask some questions about [XRName] 's children】

EXC001[i] Is [ZChildName[i]] (Gender: [XEChildGenderDis[i]]) still alive?
1. Yes
2. No

EXC003[i] When was [XEChildAliveName[i]] born? ______ [hc$([1910, 2018], int, \varnothing)$, sc$([1940, 2018], int, \varnothing)$] Year
【**IWER**: Mark the year using four digits. If the respondent does not remember the birth year of his/her child clearly, you could calculate it from information about his/her child's age now, his/her child's death year and the age of death, or age of the respondent when his/her child was born. 】

EXC004[i] What's [XEChildAliveName[i]] 's gender?
1. Male
2. Female

EXC005[i] Without taking continuing education into account, what's [XEChildAliveName[i]] 's highest achieved education?
1. No formal education (illiterate)
2. Did not finish elementary school
3. Sishu/home school
4. Elementary school
5. Middle school
6. High school
7. Vocational school
8. Two/Three-Year College/Associate degree
9. Four-Year College/Bachelor's degree
10. Master's degree
11. Doctoral degree/Ph.D
997. Don't know
999. Refuse

EXC006[i] The year before [XRName] passed away, how long had [XEChildAliveName[i]] lived with [XRName] ? ______ [hc$([0, 12], real, \varnothing)$] Month
【**IWER**: A short visit does not constitute "living together"; input 0 here if not living together at all and 12 if always living together】

EXC007[i] The year before [XRName] passed away, when [XRName] was not living with [XEChildAliveName[i]] , how often do [XRName] see [XEChildAliveName[i]] ?

1. Almost every day
2. 2-3 times a week
3. Once a week
4. Every two weeks
5. Once a month
6. Once every three months
7. Once every six months
8. Once a year
9. Almost never
10. Other

EXC008 How many grandchildren did [XRName] have before passing away? (Including children of biological children, stepchildren and adopted children) ______ [hc$([0, 25], int, \varnothing)$]

EXC009 How many great-grandchildren did [XRName] have before passing away? ______ [hc$([0, 25], int, \varnothing)$]

EXD. Health Status and Funtioning (1)

EXDA001[i] Since [ZIWTime], had a doctor diagnosed that [XRName] had [XEChroDisType[i]] ?

1. Yes
2. No

EXDA002[i] When was [XRName] first told by a doctor that he/she had [XEChroDisType[i]]?

【**IWER**: Mark the year using four digits.

Please enter "-1" if R cannot answer】

1. Year ______ [hc$([1900, 2020], int, -1)$, sc$([1920, 2020), int, -1)$] (**EXDA002_1[i]**) Year
2. Age ______ [hc$([0, 120], int, -1)$, sc$((0, 100], int, -1)$] (**EXDA002_2[i]**) Age

EXDA003 From [ZIWTime] to the death of [XRName], did he/she have a heart attack or myocardial infarction?

1. Yes
2. No

EXDA004 [XRName] When was his/her most recent heart attack?

【**IWER**: Mark the year using four digits.

Please enter "-1" if R cannot answer】

1. Year ______ [hc$([1900, 2020], int, -1)$, sc$([1920, 2020), int, -1)$] (**EXDA004_1**) Year
2. Age ______ [hc$([0, 120], int, -1)$, sc$((0, 100], int, -1)$] (**EXDA004_2**) Age

EXDA005 [XRName] In which organ or part of his/her body does he/she have cancer? Including the origins and metastasis of tumor. (Select All That Apply)

【**IWER**: We should still ask R even if he/she has already been cured.】

1. Brain
2. Oral cavity

3. Larynx
4. Other pharynx
5. Thyroid
6. Lung
7. Breast
8. Oesophagus
9. Stomach
10. Liver
11. Pancreas
12. Kidney
13. Prostate
14. Testicle
15. Ovary
16. Cervix
17. Endometrium
18. Colon or rectum
19. Bladder
20. Skin
21. Non-Hodgkin lymphoma
22. Leukemia
23. Other organ ______ (**EXDA005_1**)

EXDA006 Have [XRName] taken any of the following treatments to treat his/her cancer or relieve history/her symptoms (e.g., pain, nausea, etc.) in the past two years? (Select All That Apply)

【**IWER**: Read one by one.

(1) Chemotherapy is a method of treating diseases with chemosynthetic drugs. Chemotherapy is currently one of the main methods for treating tumors and some autoimmune diseases.

(2) Surgical therapy is the earliest applied method for treating cancer, and it is also the preferred treatment for many early cancer treatments.

(3) Radiotherapy is a method of treating tumors by irradiating them with various energies of radiation to suppress and kill cancer cells】

1. Taking Chinese traditional medicine
2. Taking Western morden medicine
3. Chemotherapy
4. Surgery
5. Radiation therapy
6. Other treatments, please spcify ______ (**EXDA006_1**)
7. None of the above

[conflict(7,[7]^c)]

EXDA007 Since [ZIWTime], did a doctor diagnosed that [XRName] had another stroke?

1. Yes
2. No

EXDA008 [XRName] In what year was his/her most recent stroke?

【**IWER**: Mark the year using four digits.
Please enter "-1" if R cannot answer】

1. Year ______ [hc($[1900, 2020], int, -1$), sc($[1920, 2020), int, -1$)] (**EXDA008_1**) Year
2. Age ______ [hc($[0, 120], int, -1$), sc($(0, 100], int, -1$)] (**EXDA008_2**) Age

EXDA009 Did [XRName] have memory problems as of one month before he/she died?

1. Yes
2. No

EXDA010 How old was [XRName] when memory problems became apparent?

【**IWER**: Mark the year using four digits.
Please enter "-1" if R cannot answer】

1. Year ______ [hc($[1900, 2020], int, -1$), sc($[1920, 2020), int, -1$)] (**EXDA010_1**) Year
2. Age ______ [hc($[0, 120], int, -1$), sc($(0, 100], int, -1$)] (**EXDA010_2**) Age

EXDA011 Did the memory problems begin suddenly or slowly?

1. Suddenly
2. Slowly

EXDA012 Did the memory problems get worse over time?

1. Yes
2. No

EXDA013 Since [ZIWTime], has [XRName] fallen down?

1. Yes
2. No

EXDA014 How many times has [XRName] fallen down seriously enough to need medical treatment? ______ [hc($[0, 99], int, \varnothing$)] Times

EXDA015 Since [ZIWTime], has [XRName] fractured his/her hip?

1. Yes
2. No

EXDA016 Was [XRName] often troubled with any body pains? Is if none, a little, somewhat, quite a bit, or very?

1. None
2. A little
3. Somewhat
4. Quite a bit
5. Very

EXDA017 Since [ZIWTime] to his/her death, Did [XRName] have any (other) major illnesses?

1. Yes
2. No

EXDA018 What illnesses? ______

EXDA019 Was there a period of at least one month during the last year of his/her life when [XRName] had severe fatigue or exhaustion?

1. Yes
2. No

EXDA020 Was there a period of at least one month during the last year of his/her life when [XRName] had loss of control of bowel or bladder?

1. Yes
2. No

EXD. Health Status and Funtioning (2)

EXDB001 Because of health or memory problem, did [XRName] have any difficulty with dressing in the last three months of his/her life? Dressing includes taking clothes out from a closet, putting them on, buttoning up, and fastening a belt?

1. Don't have any difficulty
2. Have difficulty but can still do it
3. Have difficulty and need help
4. Can not do it

EXDB002 Did anyone ever help R dress?

1. Yes
2. No

EXDB003 How long had he/she needed help with dressing?

1. ______ [hc$((0, 99], real, \emptyset)$] (**EXDB003_1**) Months
2. ______ [hc$((0, 99], real, \emptyset)$] (**EXDB003_2**) Years
3. Since ______ [hc$([1, 120], int, \emptyset)$, sc$((1, 100], int, \emptyset)$] (**EXDB003_3**) Years old
4. Since ______ [hc$([1900, 2020], int, \emptyset)$, sc$([1920, 2020], int, \emptyset)$] (**EXDB003_4**) Year

EXDB004 Because of health or memory problem, did [XRName] have any difficulty with bathing or showering in the last three months of his/her life?

1. Don't have any difficulty
2. Have difficulty but can still do it
3. Have difficulty and need help
4. Can not do it

EXDB005 Did anyone ever help R bathing or showering?

1. Yes
2. No

EXDB006 How long had he/she needed help with bathing or showering?

1. ______ [hc$((0, 99], real, \emptyset)$] (**EXDB006_1**) Months
2. ______ [hc$((0, 99], real, \emptyset)$] (**EXDB006_2**) Years
3. Since ______ [hc$([1, 120], int, \emptyset)$, sc$((1, 100], int, \emptyset)$] (**EXDB006_3**) Years old
4. Since ______ [hc$([1900, 2020], int, \emptyset)$, sc$([1920, 2020], int, \emptyset)$] (**EXDB006_4**) Year

EXDB007 Because of health or memory problem, did [XRName] have any difficulty with eating in the last three months of his/her life?

1. Don't have any difficulty
2. Have difficulty but can still do it
3. Have difficulty and need help
4. Can not do it

EXDB008 Did anyone ever help R eating?
1. Yes
2. No

EXDB009 How long had he/she needed help with eating?
1. ______ [hc$((0, 99], real, \emptyset)$] (**EXDB009_1**) Months
2. ______ [hc$((0, 99], real, \emptyset)$] (**EXDB009_2**) Years
3. Since ______ [hc$([1, 120], int, \emptyset)$, sc$((1, 100], int, \emptyset)$] (**EXDB009_3**) Years old
4. Since ______ [hc$([1900, 2020], int, \emptyset)$, sc$([1920, 2020], int, \emptyset)$] (**EXDB009_4**) Year

EXDB010 Because of health or memory problem, did [XRName] have any difficulty with getting in or out of bed in the last three months of his/her life?
1. Don't have any difficulty
2. Have difficulty but can still do it
3. Have difficulty and need help
4. Can not do it

EXDB011 Did anyone ever help R getting in or out of bed?
1. Yes
2. No

EXDB012 How long had he/she needed help with getting in or out of bed?
1. ______ [hc$((0, 99], real, \emptyset)$] (**EXDB012_1**) Months
2. ______ [hc$((0, 99], real, \emptyset)$] (**EXDB012_2**) Years
3. Since ______ [hc$([1, 120], int, \emptyset)$, sc$((1, 100], int, \emptyset)$] (**EXDB012_3**) Years old
4. Since ______ [hc$([1900, 2020], int, \emptyset)$, sc$([1920, 2020], int, \emptyset)$] (**EXDB012_4**) Year

EXDB013 Because of health or memory problem, did [XRName] have any difficulty with using the toilet in the last three months of his/her life?
1. Don't have any difficulty
2. Have difficulty but can still do it
3. Have difficulty and need help
4. Can not do it

EXDB014 Did anyone ever help R using the toilet?
1. Yes
2. No

EXDB015 How long had he/she needed help with using the toilet?
1. ______ [hc$((0, 99], real, \emptyset)$] (**EXDB015_1**) Months
2. ______ [hc$((0, 99], real, \emptyset)$] (**EXDB015_2**) Years
3. Since ______ [hc$([1, 120], int, \emptyset)$, sc$((1, 100], int, \emptyset)$] (**EXDB015_3**) Years old
4. Since ______ [hc$([1900, 2020], int, \emptyset)$, sc$([1920, 2020], int, \emptyset)$] (**EXDB015_4**) Year

EXDB016 Because of health and memory problems, did [XRName] have any difficulties with preparing hot meals in the last three months of his/her life? (Definition: By preparing hot meals, we mean preparing ingredients, cooking, and serving food)

1. Don't have any difficulty
2. Have difficulty but can still do it
3. Have difficulty and need help
4. Can not do it

EXDB017 Did anyone ever help R preparing hot meals?

1. Yes
2. No

EXDB018 How long had he/she needed help with preparing hot meals?

1. ______ [hc$((0,99],real,\varnothing)$] (**EXDB018_1**) Months
2. ______ [hc$((0,99],real,\varnothing)$] (**EXDB018_2**) Years
3. Since ______ [hc$([1,120],int,\varnothing)$, sc$((1,100],int,\varnothing)$] (**EXDB018_3**) Years old
4. Since ______ [hc$([1900,2020],int,\varnothing)$, sc$([1920,2020],int,\varnothing)$] (**EXDB018_4**) Year

EXDB019 Because of health and memory problems, did [XRName] have any difficulties with shopping for groceries in the last three months of his/her life?

1. Don't have any difficulty
2. Have difficulty but can still do it
3. Have difficulty and need help
4. Can not do it

EXDB020 Did anyone ever help R shopping for groceries?

1. Yes
2. No

EXDB021 How long had he/she needed help with shopping for groceries?

1. ______ [hc$((0,99],real,\varnothing)$] (**EXDB021_1**) Months
2. ______ [hc$((0,99],real,\varnothing)$] (**EXDB021_2**) Years
3. Since ______ [hc$([1,120],int,\varnothing)$, sc$((1,100],int,\varnothing)$] (**EXDB021_3**) Years old
4. Since ______ [hc$([1900,2020],int,\varnothing)$, sc$([1920,2020],int,\varnothing)$] (**EXDB021_4**) Year

EXDB022 Because of health and memory problems, did [XRName] have any difficulties with making phone calls in the last three months of his/her life?

【**IWER**: If R never called,and the family members assured that R can not do this because of health or memory problems based on R's condition,select "Have difficulty and need help" or "Can not do it" accordingly.】

1. Don't have any difficulty
2. Have difficulty but can still do it
3. Have difficulty and need help
4. Can not do it

EXDB023 Did anyone ever help R making telephone calls?

【**IWER**: If R had never making telephone calls, select NO】

1. Yes

2. No

EXDB024 How long had he/she needed help with making telephone calls?

1. ______ [hc((0, 99], *real*, ∅)] (**EXDB024_1**) Months
2. ______ [hc((0, 99], *real*, ∅)] (**EXDB024_2**) Years
3. Since ______ [hc([1, 120], *int*, ∅), sc((1, 100], *int*, ∅)] (**EXDB024_3**) Years old
4. Since ______ [hc([1900, 2020], *int*, ∅), sc([1920, 2020], *int*, ∅)] (**EXDB024_4**) Year

EXDB025 Because of health and memory problems, did [XRName] have any difficulties with taking medications in the last three months of his/her life? By taking medications, we mean taking the right portion of medication right on time.

1. Don't have any difficulty
2. Have difficulty but can still do it
3. Have difficulty and need help
4. Can not do it

EXDB026 Did anyone ever help R taking medications?

1. Yes
2. No

EXDB027 How long had he/she needed help with mtaking medications?

1. ______ [hc((0, 99], *real*, ∅)] (**EXDB027_1**) Months
2. ______ [hc((0, 99], *real*, ∅)] (**EXDB027_2**) Years
3. Since ______ [hc([1, 120], *int*, ∅), sc((1, 100], *int*, ∅)] (**EXDB027_3**) Years old
4. Since ______ [hc([1900, 2020], *int*, ∅), sc([1920, 2020], *int*, ∅)] (**EXDB027_4**) Year

EXDB028 Because of health and memory problems, did [XRName] have any difficulties with managing your money in the last three months of his/her life, such as paying your bills, keeping track of expenses, or managing assets?

1. Don't have any difficulty
2. Have difficulty but can still do it
3. Have difficulty and need help
4. Can not do it

EXDB029 Did anyone ever help R managing money?

1. Yes
2. No

EXDB030 How long had he/she needed help with managing money?

1. ______ [hc((0, 99], *real*, ∅)] (**EXDB030_1**) Months
2. ______ [hc((0, 99], *real*, ∅)] (**EXDB030_2**) Years
3. Since ______ [hc([1, 120], *int*, ∅), sc((1, 100], *int*, ∅)] (**EXDB030_3**) Years old
4. Since ______ [hc([1900, 2020], *int*, ∅), sc([1920, 2020], *int*, ∅)] (**EXDB030_4**) Year

EXD. Health Status and Funtioning (3)

EXDB031 Who most often helped [XRName] with (dressing, bathing, eating, getting out of bed, using the toilet, controlling urination and defecation, doing chores, preparing hot meals, shopping, managing money, making phone calls, taking medications)? (Select All That Apply)

【**IWER**: "employee(s) of facility" only for R who was living in a nursing home or hospice when he/she died 】

1. Spouse
2. Father, Mother, Father-in-law, Mother-in-law
3. Children, Children's spouses, Grandson, Granddaughter
4. Sibling, Brother-in-law, Sister-in-law, Sibling of spouse, Children of sibling, Brother-in-law of spouse, Sister-in-law of spouse, Children of brother-in-law, Children of sister-in-law
5. Other relative
6. Paid helper (such as nanny), in total ______ [hc$((0,99),int,-1)$, sc$([1,10),int,-1)$] (**EXDB031_1**) persons
7. Volunteer
8. Employee(s) of facility
9. Employee(s) of homed-based elderly care institutions
10. Community
11. Other, please specify ______ (**EXDB031_2**)

EXDB032 Father, mother, father-in-law, mother-in-law, Who helped [XRName] most? (Select All That Apply)

1. Father
2. Mother
3. Father-in-law
4. Mother-in-law

EXDB033 For the children, children-in-law, grandson, granddaughter who helped [XRName], which children's family are they from? (Select All That Apply)

1-25. [XEChildAliveName[i]]

26-35. Other child, name ______ (**EXDB033_1[i]**)

EXDB034[i] For the family members of [XEHelperChild[i]], who helps [XRName] in person? (Select All That Apply)

1. [XEHelperChild[i]] himself/herself
2. [XEHelperChild[i]] his/her spouse
3. [XEHelperChild[i]] his/her children.How many helped [XRName] in person? ______ [hc$((0,99),int,-1)$, sc$([1,10),int,-1)$] (**EXDB034_1[i]**)

EXDB035 For the siblings, spouse and children of siblings, siblings of your spouse, spouse and children of siblings of your spouse who helped [XRName], which siblings' family are they from? (Select All That Apply)

1-30. [XESibName[i]]

31-40. Other sibling, name ______ (**EXDB035_1[i]**)

EXDB036$[i]$ For the family members of [XEHelperSib[i]],who help [XRName] in person? (Select All That Apply)

1. [XEHelperSib[i]] himself/herself
2. [XEHelperSib[i]] his/her spouse
3. [XEHelperSib[i]] his/her children. how many helped [XRName] in person? ______ [hc($(0,99), int, -1$), sc($[1,10), int, -1$)] (**EXDB036_1[i]**)

EXDB037 The number of the relatives who helped [XRName] in person ______ [hc($(0,99), int, -1$), sc($[1,10), int, -1$)], What's their relationships with [XRName]? ______ (**EXDB037_1**)

【**IWER**: Please enter "-1" if R cannot answer】

EXDB038 The number of the others who helped [XRName] in person ______ [hc($(0,99), int, -1$), sc($[1,10), int, -1$)], What's their relationships with [XRName]? ______ (**EXDB038_1**)

【**IWER**: Please enter "-1" if R cannot answer】

EXDB039 From all the helpers list below, please select the most important 7 helpers for [XRName].

1-99. [XEHelper[i]]

EXDB040$[i]$ During the last month before the death of [XRName],on about how many days did [XEHelpList[i]] helped [XRName]? ______ [hc($[0,31], int, -1$)] Days

【**IWER**: Please enter "-1" if R cannot answer】

EXDB041$[i]$ On the days [XEHelpList[i]] helped [XRName],about how many hours per day is that? ______ [hc($[0,24], int, -1$)] Hours

【**IWER**: less than an hour, enter 1;
Please enter "-1" if R cannot answer】

EXDB042$[i]$ Was [XEHelpList[i]] living in [XRName]'s home when provinded help?

1. Yes
2. No

EXDB043 Did [XRName] use the following auxiliary? (Select All That Apply)

1. Walking stick
2. Travel device
3. Manual wheelchair
4. Electric Wheelchair
5. Catheter, urine collection bag
6. Toilet Series
7. None of the above

[conflict($7, [7]^c$)]

EXE. Health Care and Insurance(1)

【**INTRO**: Now we would like to know about health insurance or benefits that [XRName] might have.】

EXEA001 Was [XRName] covered by any of the types of health insurance listed below at the time of his/her death? (Select All That Apply)

1. Urban employee medical insurance (yi-bao)
2. Urban and rural resident medical insurance
3. Urban resident medical insurance
4. New cooperative medical insurance (he-zuo-yi-liao)
5. Government medical insurance (gong-fei)
6. Medical aid
7. Private medical insurance: purchased by work unit
8. Private medical insurance: purchased by individual
9. Urban non-employed person's health insurance
10. Long-term care insurance
11. Other medical insurance (specify) ______ (**EXEA001_1**)
12. No insurance

[conflict(12, $[12]^c$)]

EXEA001_verify Was [XRName] covered by any of the types of health insurance listed below at the time of his/her death?

【**IWER**:If R participated in some health insurance, return to the previous question for modification.】

1. [XRName] has no insurance

EXEA002 Does [XRName] have supplemental insurance to this plan?

【**IWER**: Supplemental insurance is relative to basic health insurance, includes private medical insurance and so on.】

1. Yes
2. No

EXEA003[i] Where did [XRName] set up his/her [XEXMIns[i]] insurance account/policy?

1. Residence before death: [EXB003_1]
2. The place of R's Hukou (If it is not in this county)
3. Other ______ (**EXEA003_1[i]**) province/city/county/district

EXEA008[i] When did [XRName] covered by [XEXMIns[i]] this benefit? ______ [hc([1900, 2020], int,)] (**EXEA008_1[i]**) year ______ [hc([1, 12], int, -1)] (**EXEA008_2[i]**) month

【**IWER**:If the R is not clear about the month, please enter "-1", and enter the year in 4-digit.】

EXEA009 What is [XRName]'s main reason for not having health insurance before his/her death? (Select All That Apply)

1. Do not need it
2. Cannot afford it
3. Do not know where or from whom to get it
4. Do not trust the institutions that offer health insurance
5. Do not have suitable programs for me to buy
6. Never thought of it
7. Others ______ (**EXEA009_1**)

EXEB001 Is [XRName] the policy holder/primary beneficiary of any of the types of health insurance listed below BEFORE? (Select All That Apply)

1. Urban employee medical insurance (yi-bao)

2. Urban and rural resident medical insurance
3. Urban resident medical insurance
4. New cooperative medical insurance (he-zuo-yi-liao)
5. Government medical insurance (gong-fei)
6. Medical aid
7. Private medical insurance: purchased by work unit
8. Private medical insurance: purchased by individual
9. Urban non-employed person's health insurance
10. Long-term care insurance
11. Other medical insurance (specify) ______ (**EXEB001_1**)
12. No insurance

[conflict$(12, [12]^c)$]

EXEB003[i] When did [XRName] lose it ([XEXPMIns[i]] ?) ______ [hc$([1900, 2020], int,)$] (**EXEB003_1[i]**) Year ______ [hc$([1, 12], int, -1)$] (**EXEB003_2[i]**) Month

【**IWER**: Mark the year using four digits. Please enter "-1" if R cannot answer the month and day.】

EXEB004[i] Why did [XRName] lose [XEXPMIns[i]] ?
1. Work unit no longer exists
2. This insurance is no longer available locally
3. I was dismissed by the unit or i quit my job
4. I don't want to participate
5. My family is not willing to pay for me
6. The insurance is too expensive
7. Other, please specify ______ (**EXEB004_1[i]**)

EXEB005 Did [XRName] 's family or friend received money from any health insurance?
1. Yes
2. No

EXEB006 How much was it? ______ [hc$([0, \infty), real, -1)$, sc$([0, 50000), real, -1)$]

EXE. Health Care and Insurance(2)

EXEC001 When did [XRName] take the last physical examination? Between last interview and his/her death.

【**IWER**: Physical examination is a comprehensive health examination to understand your health status. Mark the year using four digits. Please enter "-1" if R cannot answer the month.】
1. ______ [hc$([1900, 2020], int,)$] (**EXEC001_1**) Year ______ [hc$([1, 12], int, -1)$] (**EXEC001_2**) Month
2. Didn't take physical examination since last interview

EXED030 Aside from any hospital stays, how many times did [XRName] visited a public hospital, private hospital, public health center, clinic, or health worker's or doctor's practice, or been visited by a health worker or doctor for outpatient care in the one month before his/her death? ______ [hc$([0, \infty), int,)$, sc$([0, 10], int,)$] Times

EXED031 How much did all the cost? ______ [hc$([0,\infty),real,-1)$, sc$([0,30000],real,-1)$, ub$([-1],[50,100,200,500,1000])$] Yuan

【**IWER**:Please enter "-1" if R cannot answer.】

EXED032 Self-paid part?

【**IWER**:Please enter "-1" if R cannot answer.】

1. Self-paid part ______ [hc$((0,\infty),real,-1)$, sc$((0,30000],real,-1)$, ub$([-1],[50,100,250,500,800])$] (**EXED032_1**) Yuan
2. Didn't pay anything

EXED033 What insurance did [XRName] use or will use? (Select All That Apply)

1-11. [XEXMIns[i]]
12. Reimbursed by [XRName]'s union
13. No insurance
14. Not revelent to [XRName]

EXEF001 Did [XRName] Take any purchased medicine in the month before his/her died?

【**IWER**:It does not include the medicine bought at the drugstore with the doctor's prescription, but taking the medicine you have saved or the medicine given by others is also considered as buying medicine by yourself.】

1. Yes
2. No

EXEF002 How much did all the cost? ______ [hc$([0,\infty),real,-1)$, sc$([0,2000],real,-1)$, ub$([-1],[10,30,100,200,300])$] Yuan

【**IWER**:Please enter "-1" if R cannot answer.】

EXEF003 Self paid part?

【**IWER**:Please enter "-1" if R cannot answer.】

1. Self paid part: ______ [hc$((0,\infty),real,-1)$, sc$((0,2000],real,-1)$, ub$([-1],[10,30,70,100,200])$] (**EXEF003_1**) Yuan
2. Didn't pay anything

EXEF005 What insurance did R use or will you use?

1-11. [XEXMIns[i]]
12. Reimbursed by [XRName]'s union
13. No insurance
14. Not revelent to [XRName]

EXEG000 Was [XRName] dead in hospital?

1. Yes
2. No

EXEG001 Earlier you told me that [XRName] died while in a hospital. How long had he/she been a patient in that hospital before his/her death?

【**IWER**: Enter "1 hour" if less than one hour.】

1. ______ [hc$([0,24),real,-1)$] (**EXEG001_1**) Hours
2. ______ [hc$([1,7),real,-1)$] (**EXEG001_2**) Days

3. ______ [hc([1, 5), *real*, −1)] (**EXEG001_3**) Weeks
4. ______ [hc([1, 12), *real*, −1)] (**EXEG001_4**) Months
5. ______ [hc([1, ∞), *real*, −1)] (**EXEG001_5**) Years

EXEG002 Why had he/she been admitted to the hospital? Was it to have surgery, receive other treatments, relieve his/her symptoms, or what?

1. Surgery
2. Receive other treatments
3. Relieve symptoms
4. Others ______ (**EXEG002_1**)

EXEG003 In addition to that hospital stay, in the one month before his/her death had he/she been a patient in a hospital overnight?

1. Yes
2. No

EXEG004 In the one year before his/her death had he/she been a patient in a hospital overnight?

1. Yes
2. No

EXEG005 How many different times was he/she a patient in a hospital overnight in the one year before his/her death (including his/her final hospitalization)? ______ [hc((0, ∞), *int*,)] Times

EXEG006 During any of those hospital stays did [XRName] spend any time in an intensive care unit?

1. Yes, ______ [hc([1, 366], *int*, −1) , sc([1, 180], *int*, −1)] (**EXEG006_1**) Days
2. No

EXEG007 During any of those hospital stays did [XRName] use life support equipment, such as a respirator? (Select All That Apply)

1. Respirator
2. Artificial liver
3. Artificial lung
4. None of above

[conflict(4, $[4]^c$)]

EXEG008 During any of those hospital stays did [XRName] use kidney dialysis services?

1. Yes
2. No

EXEG009 During any of those hospital stays did [XRName] receive antibiotics to treat pneumonia or other infection?

1. Yes
2. No

EXEG010 About how much did [XRName] pay out-of-pocket for hospital bills in the one year before his/her death (Only include fees paid to the hospital, including ward fees but excluding wages paid to a hired nurse, transportation costs, and accommodation costs

for yourself or family members) ? ______ [hc($[0,\infty)$, *int*, −1), sc($[0,300000]$, *int*, −1), ub($[-1], [1500,3000,7000,15000,30000]$)] Yuan

【**IWER**:Please enter “-1” if R doesn't clear about it.】

EXEG011 Self-paid part?

【**IWER**:Please enter “-1” if R doesn't clear about it.】

1. Self-paid part: ______ [hc($(0,\infty)$, *real*, −1), sc($(0,300000]$, *real*, −1), ub($[-1], [1000,2000, 5000,10000,20000]$)] (**EXEG011_1**) Yuan
2. Didn’t pay anything

EXEG012 What insurance did you use or will you use? (Select All That Apply)

1-11. [XEXMIns[i]]
12. Reimbursed by [XRName]’s union
13. No insurance
14. Not revelent to [XRName]

EXF. Work and Retirement

EXF001 According to our records, in the last interview on [ZIWTime], [XRName] was working, in what month and year did he/she stop working?

【**IWER**: If R cannot answer year and month, enter “-1”.】

1. ______ [hc([ZIWYear,XIWYear], *int*, −1)] (**EXF001_1**) Year ______ [hc($[1,12]$, *int*, −1)] (**EXF001_2**) Month
2. [XRName] was working until death

995. [XRName] was not working in the last interview
997. Don’t know
999. Refuse

EXF002 In the week before [XRName] stopped working, how many days was he/she working? ______ [hc($[1,7]$, *int*, −1)] days

【**IWER**: If R cannot answer, please enter “-1”. 】

EXF003 In the week before [XRName] stopped working, on average, how many hours was [XRName] working? ______ [hc($[1,24]$, *int*, ∅), sc($[1,16]$, *int*, ∅)] (**EXF003_1**) hours per day, in which how many hours were for agricultural activities? ______ [hc([0,EXF003_1], *int*, −1)] (**EXF003_2**) hours per day, and how many hours were for non-agricultural activities? ______ [hc([0,EXF003_1], *int*, −1)] (**EXF003_3**) hours per day.

【**IWER**: The sum of hours spent on agricultural and non-agricultural activities should be equal to the total of working hours.
If R cannot answer, enter “-1”. 】

EXF004 According to our records, in the last interview, [XRName] had not processed retirement, had he/she processed retirement before he/she died?

1. Yes
2. No

997. Don’t know
999. Refuse

EXFN. Pension

EXF005 How much have been inherited from pensions? ______ [hc$([0, 10000000], int, -1)$] Yuan

【**IWER**:Please enter "-1" if R doesn't clear about it.】

EXF006 Did [XRName] receive a lump-sum compensation from pensions?

1. Yes
2. No

EXF007 How much was it? ______ [hc$([10, 10000000], int, -1)$] Yuan

【**IWER**:Please enter "-1" if R doesn't clear about it.】

EXF008 Did [XRName] receive a lump-sum compensation from other places?

1. Yes
2. No

EXF009 How much was it? ______ [hc$([10, 10000000], int, -1)$] Yuan

【**IWER**:Please enter "-1" if R doesn't clear about it.】

EXF010 Was [XRName] insured by life insurance?

1. Yes
2. No

EXF011 How much did the life insurance pay upon the death of [XRName]? ______ [hc$([10, 10000000], int, -1)$] Yuan

【**IWER**:Please enter "-1" if R doesn't clear about it.】

EXF012 Did [XRName] have commercial pension?

1. Yes
2. No

EXF013 How much have been inherited from commercial pension? ______ [hc$([10, 10000000], int, -1)$] Yuan

【**IWER**:Please enter "-1" if R doesn't clear about it.】

EXG. Incomes, Expenditures and Assets

EXG001 When [XRName] died, did [XRName] have house property? If had, what was the total value of all houses? Just calculate the value of the part of the property right [XRName] owned.

【**IWER**: Please enter "-1" if R cannot answer.】

1. Had house property, total value is ______ [hc$((0, \infty), int, -1)$, sc$((0, 1000000], int, -1)$] (**EXG001_1**) Yuan
2. Did not have house property

EXG002 How was [XRName]'s house disposed? What is the relationship between inheritors and [XRName], how many percent did them inherit respectively? If house was sold, please also report the inheritors' percentage.

【**IWER**: Please enter “-1” if R cannot answer.】

1. Spouse ______ [hc$((0,100], real, -1)$] (**EXG002_1**) %
2. Child, son-in-law, daughter-in-law
3. Sibling ______ [hc$((0,100], real, -1)$] (**EXG002_3**) %
4. Other relative ______ [hc$((0,100], real, -1)$] (**EXG002_4**) %
5. Parents/parents-inlaw ______ [hc$((0,100], real, -1)$] (**EXG002_5**) %
6. Grandchildren
7. Friends ______ [hc$((0,100], real, -1)$] (**EXG002_7**) %
8. Charity ______ [hc$((0,100], real, -1)$] (**EXG002_8**) %
9. Others, please specify ______ (**EXG002_9_1**) , ______ [hc$((0,100], real, -1)$] (**EXG002_9**) %
10. Do not distribute

[conflict$(10,[10]^c)$]

EXG003 Which child inherited the house? Children's spouse's portion is counted as children. If children are not in the list, please add children's name. (Select All That Apply)

1-25. [ZChildName[i]]
26-35. Other child, the name is ______ (**EXG003_1[i]**)

EXG004[i] [XEXGChildList1[i]] inherited ______ [hc$((0,100], real, -1)$] %.

【**IWER**: Please enter “-1” if R cannot answer.】

EXG007 Which child's children inherited the house? If Grandchildren's parents are not in the list, please add children's name. (Select All That Apply)

1-25. [ZChildName[i]]
26-35. Other child, the name is ______ (**EXG007_1[i]**)

EXG008[i] [XEXGChildList4[i]]'s children inherited ______ [hc$((0,100], real, -1)$] %.

【**IWER**: Please enter “-1” if R cannot answer.】

EXG009 Excluding house property, medical insurance account, pension and compensation, other compensation,life insurance payment and commercial pension, did [XRName] leave any cash?

1. Yes
2. No

EXG010 When [XRName] died, how much was the cash? ______ [hc$((0,\infty), int, -1)$, sc$((0,100000), int, -1)$] Yuan

【**IWER**: Please enter “-1” if R cannot answer.】

EXG011 Excluding house property, medical insurance account, pension and compensation, other compensation, life insurance payment and commercial pension, did [XRName] leave deposit?

1. Yes
2. No

EXG012 When [XRName] died, how much was the deposit? ______ [hc$((0,\infty), int, -1)$, sc$((0,100000), int, -1)$] Yuan

【IWER: Please enter “-1” if R cannot answer.】

EXG013 Excluding house property, medical insurance account, pension and compensation, other compensation,life insurance payment and commercial pension, did [XRName] leave financial assets, such as stock, fund, treasury bill?

1. Yes
2. No

EXG014 When [XRName] died, how much was the financial assets? ______ [hc$((0,\infty), int, -1)$, sc$((0,100000), int, -1)$] Yuan

【IWER: Please enter “-1” if R cannot answer.】

EXG015 Excluding house property, medical insurance account, pension and compensation, other compensation, life insurance payment and commercial pension, did [XRName] leave in-kind?

1. Yes
2. No

EXG016 When [XRName] died, what was the value of in-kind? ______ [hc$((0,\infty), int, -1)$, sc$((0,100000), int, -1)$] Yuan

【IWER: Please enter “-1” if R cannot answer.】

EXG017 Excluding house property, medical insurance account, pension and compensation, other compensation, life insurance payment, commercial pension, cash, deposit, financial assets and in-kind, did [XRName] leave other legacy? If had, what was the value? ______ [hc$([0,\infty), int, -1)$, sc$([0,100000), int, -1)$] Yuan

【IWER: Please enter “-1” if R cannot answer.】

EXG018 Excluding house property, [XRName]'s legacy, such as medical insurance account, pension and compensation, other compensation, life insurance payment, commercial pension, cash, deposit, financial assets, in-kind and others, was the total value [XEXGTotalValue] correct?

【IWER: Please enter “-1” if R cannot answer.】

1. Yes
2. No, the true value was ______ [hc$([0,\infty), int, -1)$, sc$([0,200000], int, -1)$] (**EXG018_1**) Yuan

EXG019 Did [XRName] make a will before his/her death?

1. Yes
2. No

EXG020 Was [XRName]'s will notarized?

1. Yes
2. No

EXG021 In [XRName]' will, how to assign his/her legacy? Excluding house. (Select All That Apply)

【IWER: Please enter “-1” if R cannot answer.】

1. Spouse ______ [hc$((0,100], real, -1)$] (**EXG021_1**) %

2. Child, son-in-law, daughter-in-law
3. Sibling ______ [hc((0, 100], *real*, −1)] (**EXG021_3**) %
4. Other relative ______ [hc((0, 100], *real*, −1)] (**EXG021_4**) %
5. Parents/Parents-in-law ______ [hc((0, 100], *real*, −1)] (**EXG021_5**) %
6. Grandchildren
7. Friends ______ [hc((0, 100], *real*, −1)] (**EXG021_7**) %
8. Charity ______ [hc((0, 100], *real*, −1)] (**EXG021_8**) %
9. Others, please specify ______ (**EXG021_9_1**) , ______ [hc((0, 100], *real*, −1)] (**EXG021_9**) %
10. Do not distribute

[conflict(10, $[10]^c$)]

EXG022 Which child inherited the legacy? Children's spouse's portion is counted as children. If children are not in the list, please add children's name. (Select All That Apply)

1-25. [ZChildName[i]]
26-35. Other child, the name is ______ (**EXG022_1[i]**)

EXG023[i] [XEXGChildList2[i]] inherited ______ [hc((0, 100], *real*, −1)] %.

【**IWER**: Please enter "-1" if R cannot answer.】

EXG026 Which child's child inherited the legacy? If Grandchildren's parents are not in the list, please add children's name. (Select All That Apply)

1-25. [ZChildName[i]]
26-35. Other child, the name is ______ (**EXG026_1[i]**)

EXG027[i] [XEXGChildList5[i]]'s children inherited ______ [hc((0, 100], *real*, −1)] %.

【**IWER**: Please enter "-1" if R cannot answer.】

EXG028 In the will, did siblings share equally for inheritance to siblings?

1. Yes
2. No

EXG029 How much is the value of inheritance excluding funeral expenses? Excluding house.

【**IWER**: Please enter "-1" if R cannot answer.】

1. Still inheritance, the value is ______ [hc((0, ∞), *int*, −1) , sc((0, 200000], *int*, −1)] (**EXG029_1**) Yuan
2. No inheritance

EXG030 Did people disposal [XRName]'s inheritance according to his/her will?

1. Yes
2. No

EXG031 How was [XRName]'s legacy disposed? (Select All That Apply)

【**IWER**: Please enter "-1" if R cannot answer.】

1. Spouse ______ [hc((0, 100], *real*, −1)] (**EXG031_1**) %
2. Child, son-in-law, daughter-in-law
3. Sibling ______ [hc((0, 100], *real*, −1)] (**EXG031_3**) %
4. Other relative ______ [hc((0, 100], *real*, −1)] (**EXG031_4**) %

5. Parents/Parents-in-law ______ [hc((0, 100], *real*, −1)] (**EXG031_5**) %
6. Grandchildren
7. Friends ______ [hc((0, 100], *real*, −1)] (**EXG031_7**) %
8. Charity ______ [hc((0, 100], *real*, −1)] (**EXG031_8**) %
9. Others, please specify ______ (**EXG031_9_1**) , ______ [hc((0, 100], *real*, −1)] (**EXG031_9**) %
10. Do not distribute

[conflict(10, $[10]^c$)]

EXG032 Which child inherited the legacy? Children's spouse's portion is counted as children. If children are not in the list, please add children's name. (Select All That Apply)

1-25. [ZChildName[*i*]]

26-35. Other child, the name is ______ (**EXG032_1[i]**)

EXG033[*i*] [XEXGChildList3[*i*]] inherited ______ [hc((0, 100], *real*, −1)] %.

【**IWER**: Please enter "-1" if R cannot answer.】

EXG036 Which child's child inherited the legacy? If Grandchildren's parents are not in the list, please add children's name. (Select All That Apply)

1-25. [ZChildName[*i*]]

26-35. Other child, the name is ______ (**EXG036_1[i]**)

EXG037[*i*] [XEXGChildList6[*i*]]'s children inherited ______ [hc((0, 100], *real*, −1)] %.

【**IWER**: Please enter "-1" if R cannot answer.】

EXK. Funeral

K01 What kind of funeral did [XRName] use after his/her death?

1. Cremation
2. Ground burial
3. Sky burial
4. Water burial
5. Other, please specify ______ (**K01_1**)

K02 Where was [XRName] buried?

1. Contracted land
2. Collective burial ground
3. Commercial burial ground
4. Waster land
5. Ashes kept in funeral home
6. Other, please specify ______ (**K02_1**)

K03 How much does the burial ground cost?

1. Total ______ [hc([0, ∞), *real*, −1) , sc([0, 50000), *real*, −1)] (**K03_1**) Yuan
2. ______ [hc([0, ∞), *real*, −1) , sc([0, 30000), *real*, −1)] (**K03_2**) Yuan/Year

K04 In which year was the burial ground purchased? ______ [hc([1900, 2020], *int*,)] Year

K06 Who paid for the burial ground or funeral home? (Select All That Apply)

1. [XRName]'s spouse
2-26. [XKChildName[i]]
27-51. [XKChildAndS[i]]
52. Grandson
53. Granddaughter
54. Relatives
55. [XRName]'s union
56. [XRName]'s insurance
57. Other people

K07 Which child was the parent of the grandchild?

1-25. [ZChildName[i]]

K08 What is that person's relationship with [XRName] ? ______

K09 How much did the whole funeral cost (including costs of coffin/cinerary casket, portrait and funeral ceremony, etc, excluding grave cost)? Total ______ [hc($[0,\infty), real, -1$), sc($[0, 100000), real, -1$), ub($[-1], [1000, 3000, 5000, 8000, 10000]$)] Yuan

K10 Who paid for the money? (Select All That Apply)

1. [XRName]'s spouse
2-26. [XKChildName[i]]
27-51. [XKChildAndS[i]]
52. Grandson
53. Granddaughter
54. Relatives
55. [XRName]'s union
56. [XRName]'s insurance
57. Other people

K11 Which child was the parent of the grandchild?

1-25. [ZChildName[i]]

K12 What is that person's relationship with [XRName]? ______

K13 How much money did [XRName]'s family receive from the whole funeral? Total ______ [hc($[0,\infty), int, -1$), sc($[0, 50000], int, -1$), ub($[-1], [1000, 3000, 5000, 8000, 10000]$)] Yuan

EXV. COVID

EXV001 Was [XRName] ever identified as a confirmed case of COVID-19 prior to his death?

1. Yes
2. No
999. Refuse

EXV002 Was [XRName] ever identified as a suspected case of COVID-19 prior to his death?

1. Yes

2. No
999. Refuse

EXV003 Was [XRName] hospitalized for treatment of COVID-19? Total days of stay in hospital ______ [hc([0, 250], *int*, −1)] days?

【**IWER**: Enter "0" if not hospitalized. If R cannot answer, enter "-1". 】

EXV004 Has the [XRName] ever been placed under quarantine or medical observation for any of the following reasons, if not considering hospitalization time? Home isolation and building closure both count as quarantine. (Select All That Apply)

1. Travel (including return to work after the Chinese New Year)
2. Close contact of a COVID-19 case
3. Apartment or residential building was closed (excluding closure of the residential area)
4. Asked to be quarantined when going to hospital or after being discharged
5. Nucleic acid test shows positive
6. No quarantine experience (EXCLUSIVE)
997. Don't know
999. Refuse

[conflict(6, 997, 999, $[6, 997, 999]^c$)]

EXV005 [XRName] Total days of quarantine: ______ [hc([1, 250], *int*, −1), sc([1, 14], *int*, −1)] days

【**IWER**: For multiple quarantine experiences, please count all the days. If R cannot answer, enter "-1". 】

EXV006 Where was the place of [XRName]'s quarantine? (Select All That Apply)

1. Hospital
2. A centralized medical observation site such as a hotel
3. Own residence
4. Other: ______ (**EXV006_1**)
999. Refuse

[conflict(999, $[999]^c$)]

EXV007 During [XRName]'s quarantine, was [XRName] ever accompanied by another person living together?

【**IWER**: In the case of multiple quarantine experiences, answer "yes" as long as there was once a quarantine with another person.】

1. Yes
2. No
999. Refuse

EXV008 Has [XRName] ever been tested for the coronavirus?

【**IWER**: If R cannot answer the month, enter "-1". 】

1. Yes, last time tested in ______ [hc([1, 12], *int*, −1)] (**EXV008_1**) Month
2. No
999. Refuse

EXV009 During the pandemic, [XEXVCNotInQuarantine], did [XRName] ever stay home for sev-

eral days for fear of infection? What was the longest period of time this happened? ______ [hc([0, 250], *int*, −1)] days (If no such case or going out every day, enter “0”.)
【**IWER**: If R cannot answer, please enter “-1”. 】

EXV010 During the pandemic, did [XRName] ever need to go to see a doctor, including a dental appointment, but was forced to postpone or cancel it due to the pandemic?
1. Yes
2. No

EXV011 Why was [XRName]’s plan to see a doctor postponed or cancelled? (Select All That Apply; Don’t Read The Answers)
1. No appointment could be made or regular hospital appointments had to be cancelled
2. Hospitals rescheduled all regular appointments
3. [XRName] decided to wait
4. [XRName] was afraid of going to the hospital
5. Other, please specify ______ (**EXV011_1**)

EXV012 Can you specify what treatment or medical care [XRName] was trying to get during the pandemic, but the plan ended being postponed or cancelled? (Select All That Apply)
1. Major surgery requiring hospitalization
2. Minor surgery that can be done on an outpatient basis or in a day
3. To see a doctor for a regular outpatient visit
4. To get prescription drugs
5. To see a dentist for oral treatment
6. Other, please specify ______ (**EXV012_1**)

EXV013 Was [XRName]’s intention of going to an outpatient clinic during the pandemic to treat a new symptom or disease, to treat a disease [XRName] already had, or to have routine medical screening? (Select All That Apply)
1. A new symptom or disease
2. Treatment of an existing condition
3. Routine medical screening

EXV014 In your opinion, was [XRName]’s death related to COVID-19 or the pandemic? (Select All That Apply)
1. Due to confirmed infection with COVID-19
2. Unable to have timely access to routine medical care
3. Unable to receive emergency treatment in time
4. Unable to receive care
5. Accident caused by the pandemic
6. Other reasons, please specify ______ (**EXV014_1**)
7. Not related to the pandemic

[conflict(7, $[7]^c$)]

VA. Cause of Death

EX004 Name of verbal autopsy respondent ______

EX005 What is your relationship to the deceased?

1. Father
2. Mother
3. Spouse
4. Sibling
5. Other relative ______ (**EX005_1**)
6. No relation

EX006 Phone number of verbal autopsy respondent: ______

VAS41 Could you tell me about the illness/events that led to her his/death? ______
Take photos of medical documents such as medical records: ______ (**VAS41_1**)

VAS42 Could you tell me about the cause of death? ______
Take photos of medical documents such as medical records: ______ (**VAS42_1**)

VAS43 Could you tell me about the cause of death? ______
【**IWER**:Please enter “-1” if R cannot answer.】

Auxiliary Variable Definition

XRDeathTime Date of death

```
if (!empty("XRDeathYear") && !empty("XRDeathMonth") && !empty("XRDeathDate") &&
    !equal("XRDeathMonth", "-1") && !equal("XRDeathDate", "-1")) {
    add("XRDeathTime",
    ↪  value("XRDeathYear")+"Year"+value("XRDeathMonth")+"Month"+value("XRDeathDate")+"Day")
}

if (empty("XRDeathYear") || empty("XRDeathMonth") || empty("XRDeathDate") || equal("XRDeathMonth", "-1")
↪  ||
    equal("XRDeathDate", "-1")) {
    add("XRDeathTime", value("EXB001_1")+"Year"+value("EXB001_2")+"Month"+value("EXB001_3")+"Day")
}
```

XAliveResidenceFull Residencial address

```
if (equal("EXB003", "1")) {
    add("XAliveResidenceFull",value("EXB003_1")+value("EXB003_2")+value("EXB003_3"))
}
```

XEZDisease Generate Chronic Disease in Last Wave

```
if (equal("ZDisease[4]", "1")) {
    add("XEZDisease[1]", "1")
}
if (equal("ZDisease[5]", "1")) {
    add("XEZDisease[2]", "1")
}
if (equal("ZDisease[7]", "1")) {
    add("XEZDisease[3]", "1")
}
if (equal("ZDisease[8]", "1")) {
    add("XEZDisease[4]", "1")
}
if (equal("ZDisease[11]", "1")) {
```

```
    add("XEZDisease[5]", "1")
}
```

XEChroDisType Types of Chronic Disease

```
add("XEChroDisType", ["Cancer or malignant tumor (excluding minor skin cancers)", "Chronic lung diseases,
↪  such as chronic bronchitis , emphysema ( excluding tumors, or cancer)", "Heart attack, coronary heart
↪  disease, angina, congestive heart failure, or other heart problems", "Stroke", "Emotional, nervous, or
↪  psychiatric problems"])
```

XEDisease[3]Heart Diseases

```
if ( equal("EXDA001[3]", "1") || equal("XEZDisease[3]", "1") ) {
    add("XEDisease[3]", "1")
} else {
    add("XEDisease[3]", "2")
}
```

XEDisease[1]Cancer

```
if (equal("EXDA001[1]", "1") || equal("XEZDisease[1]", "1")) {
    add("XEDisease[1]", "1")
} else {
    add("XEDisease[1]", "2")
}
```

XEDisease[4]Stroke

```
if (equal("EXDA001[4]", "1") || equal("XEZDisease[4]", "1")) {
    add("XEDisease[4]", "1")
} else {
    add("XEDisease[4]", "2")
}
```

XEHelperSelect Has Anyone Helped

```
if (equal("EXDB002", "1") || equal("EXDB005", "1") || equal("EXDB008", "1") || equal("EXDB011", "1") ||
↪  equal("EXDB014", "1") || equal("EXDB017", "1") || equal("EXDB020", "1") || equal("EXDB023", "1") ||
↪  equal("EXDB026", "1") || equal("EXDB029", "1") ) {
    add("XEHelperSelect", "1")
} else {
    add("XEHelperSelect", "0")
}
```

XEHelperChild Names of Children Who Provide Help

```
for (var i1 = 1; i1 <= 25; i1++) {
    add("XEHelperChild[i1]", value("XEChildAliveName[i1]"))
}
for (var i1 = 26; i1 <= 35; i1++) {
    add("XEHelperChild[i1]", value("EXDB033_1[i1]"))
}
```

XESibName Names of Siblings Who Provide Help Names of Siblings Who Provide Help

```
for (var i1 = 1; i1 <= 15; i1++) {
    if (!empty("ZSibName[i1]")) {
        add("XESibName[i1]", pre("XRName")+"his/her siblings"+pre("ZSibName[i1]"))
    }
}
for (var i1 = 1; i1 <= 15; i1++) {
    if (!empty("ZSibNameS[i1]")) {
        add("XESibName["+(i1+15)+"]", pre("XRName")+"his/her spouse's siblings"+pre("ZSibNameS[i1]"))
    }
}
```

XEHelperSib Names of Siblings Who Provide Help

```
for (var i1 = 1; i1 < 31; i1++) {
    add("XEHelperSib[i1]", value("XESibName[i1]"))
}
for (var i1 = 31; i1 <= 40; i1++) {
    add("XEHelperSib[i1]", value("EXDB035_1[i1]"))
}
```

XEHelperNum Numbers of Helpers

```
add("XEHelperNum", "0")
if (selected("EXDB031", "1")) {
    add("XEHelperNum", value("XEHelperNum")+1)
    add("XEHelper["+value("XEHelperNum")+"]", "Spouse")
}
if (selected("EXDB031", "5")) {
    add("XEHelperNum", value("XEHelperNum")+1)
    add("XEHelper["+value("XEHelperNum")+"]", "Other relative")
}

if (selected("EXDB031", "6")) {
    add("XEHelperNum", value("XEHelperNum")+1)
    add("XEHelper["+value("XEHelperNum")+"]", "Paid helper")
}

if (selected("EXDB031", "7")) {
    add("XEHelperNum", value("XEHelperNum")+1)
    add("XEHelper["+value("XEHelperNum")+"]", "Volunteer")
}

if (selected("EXDB031", "8")) {
    add("XEHelperNum", value("XEHelperNum")+1)
    add("XEHelper["+value("XEHelperNum")+"]", "Employee(s) of facility")
}

if (selected("EXDB031", "9")) {
    add("XEHelperNum", value("XEHelperNum")+1)
    add("XEHelper["+value("XEHelperNum")+"]", "Employee(s) of home-based elderly care institutions")
}

if (selected("EXDB031", "10")) {
    add("XEHelperNum", value("XEHelperNum")+1)
    add("XEHelper["+value("XEHelperNum")+"]", "Community")
}

if (selected("EXDB031", "11")) {
    add("XEHelperNum", value("XEHelperNum")+1)
    add("XEHelper["+value("XEHelperNum")+"]", "Others")
}
if (selected("EXDB032", "1")) {
    add("XEHelperNum", value("XEHelperNum")+1)
    add("XEHelper["+value("XEHelperNum")+"]", "Father")
}
if (selected("EXDB032", "2")) {
    add("XEHelperNum", value("XEHelperNum")+1)
    add("XEHelper["+value("XEHelperNum")+"]", "Mother")
}
if (selected("EXDB032", "3")) {
    add("XEHelperNum", value("XEHelperNum")+1)
    add("XEHelper["+value("XEHelperNum")+"]", "Father-in-law")
}
if (selected("EXDB032", "4")) {
    add("XEHelperNum", value("XEHelperNum")+1)
    add("XEHelper["+value("XEHelperNum")+"]", "Mother-in-law")
}
for (var i1 = 1; i1 < 26; i1++) {
    if (selected("EXDB033", i1) && selected("EXDB034[i1]", "1")) {
        add("XEHelperNum", value("XEHelperNum")+1)
        add("XEHelper["+value("XEHelperNum")+"]", value("XEChildAliveName[i1]")+"himself/herself")
    }
    if (selected("EXDB033", i1) &&  selected("EXDB034[i1]", "2")) {
        add("XEHelperNum", value("XEHelperNum")+1)
        add("XEHelper["+value("XEHelperNum")+"]", value("XEChildAliveName[i1]")+"his/her spouse")
    }
    if (selected("EXDB033", i1) &&  selected("EXDB034[i1]", "3")) {
        add("XEHelperNum", value("XEHelperNum")+1)
        add("XEHelper["+value("XEHelperNum")+"]", value("XEChildAliveName[i1]")+"his/her children")
    }
}
for (var i1 = 26; i1 <= 35; i1++) {
    if (selected("EXDB033", i1) && selected("EXDB034[i1]", "1")) {
        add("XEHelperNum", value("XEHelperNum")+1)
        add("XEHelper["+value("XEHelperNum")+"]", value("EXDB033_1[i1]")+"himself/herself")
```

```
        }
        if (selected("EXDB033", i1) &&  selected("EXDB034[i1]", "2")) {
            add("XEHelperNum", value("XEHelperNum")+1)
            add("XEHelper["+value("XEHelperNum")+"]", value("EXDB033_1[i1]")+"his/her spouse")
        }
        if (selected("EXDB033", i1) &&  selected("EXDB034[i1]", "3")) {
            add("XEHelperNum", value("XEHelperNum")+1)
            add("XEHelper["+value("XEHelperNum")+"]", value("EXDB033_1[i1]")+"his/her children")
        }
    }
    for (var i1 = 1; i1 < 31; i1++) {
        if (selected("EXDB035", i1) &&  selected("EXDB036[i1]", "1")) {
            add("XEHelperNum", value("XEHelperNum")+1)
            add("XEHelper["+value("XEHelperNum")+"]", value("XESibName[i1]")+"himself/herself")
        }
        if (selected("EXDB035", i1) &&  selected("EXDB036[i1]", "2")) {
            add("XEHelperNum", value("XEHelperNum")+1)
            add("XEHelper["+value("XEHelperNum")+"]", value("XESibName[i1]")+"his/her spouse")
        }
        if (selected("EXDB035", i1) &&  selected("EXDB036[i1]", "3")) {
            add("XEHelperNum", value("XEHelperNum")+1)
            add("XEHelper["+value("XEHelperNum")+"]", value("XESibName[i1]")+"his/her children")
        }
    }
    for (var i1 = 31; i1 <= 40; i1++) {
        if (selected("EXDB035", i1) &&  selected("EXDB036[i1]", "1")) {
            add("XEHelperNum", value("XEHelperNum")+1)
            add("XEHelper["+value("XEHelperNum")+"]", value("EXDB035_1[i1]")+"himself/herself")
        }
        if (selected("EXDB035", i1) &&  selected("EXDB036[i1]", "2")) {
            add("XEHelperNum", value("XEHelperNum")+1)
            add("XEHelper["+value("XEHelperNum")+"]", value("EXDB035_1[i1]")+"his/her spouse")
        }
        if (selected("EXDB035", i1) &&  selected("EXDB036[i1]", "3")) {
            add("XEHelperNum", value("XEHelperNum")+1)
            add("XEHelper["+value("XEHelperNum")+"]", value("EXDB035_1[i1]")+"his/her children")
        }
    }
```

XEHelper Names or Identities of Helpers

```
    see above
```

XEHelpList Main Helpers Been Select

```
    for (var i1 = 1; i1 < value("XEHelperNum")+1; i1++) {
        if ( greater("XEHelperNum", "7") && !equal("XEHelperNum", "7") && selected("EXDB039", i1) ) {
            add("XEHelpList[i1]", value("XEHelper[i1]"))
        }
    }
    for (var i1 = 1; i1 < value("XEHelperNum")+1; i1++) {
        if ( !greater("XEHelperNum", "7") ) {
            add("XEHelpList[i1]", value("XEHelper[i1]"))
        }
    }
```

XESelectNum XSelectNum Chose More Than 7 Helpers or Not

```
    add("XESelectNum", "0")
    for (var i1 = 1; i1 < 99; i1++) {
        if (selected("EXDB039", i1)) {
            add("XESelectNum", value("XESelectNum")+1)
        }
    }
```

XEChildAndS If missing child's name, display his/her spouse

```
    if (empty("ZChildName[i]")) {
        add("XEChildAndS[i]", "")
    } else {
        add("XEChildAndS[i]", value("ZChildName[i]")+"的配偶")
    }
```

XEChildGenderDis Gender preloaded

```
if (equal("ZChildGender[i]", "1")) {
    add("XEChildGenderDis[i]", "Male")
} else if (equal("ZChildGender[i]", "2")) {
    add("XEChildGenderDis[i]", "Female")
} else {
    add("XEChildGenderDis[i]", "Missing")
}
```

XEChildAlive Whether the child is alive or not

```
if (equal("EXC001[i]", "1")) {
    add("XEChildAlive[i]", "1")
} else if (equal("EXC001[i]", "2")) {
     add("XEChildAlive[i]", "0")
}
```

XEChildAliveName Names of surviving children

```
if (equal("XEChildAlive[i]", "1")) {
    add("XEChildAliveName[i]", value("ZChildName[i]"))
}
```

XEChildBirth Child's year of birth

```
if (!empty("ZChildBirth[i]")) {
    add("XEChildBirth[i]", value("ZChildBirth[i]"))
} else {
    add("XEChildBirth[i]", value("EXC003[i]"))
}
```

XEChildGender Gender of child

```
if (!empty("ZChildGender[i]")) {
    add("XEChildGender[i]", value("ZChildGender[i]"))
} else {
    add("XEChildGender[i]", value("EXC004[i]"))
}
```

XEChildEdu Maximum education level of childrenc

```
if (!empty("ZChildEdu[i]")) {
    add("XEChildEdu[i]", value("ZChildEdu[i]"))
} else {
    add("XEChildEdu[i]", value("EXC005[i]"))
}
```

XEXMIns Type of health insurance

```
add("XEXMIns[1]", "Urban employee medical insurance:yi-bao")
add("XEXMIns[2]", "Urban and rural resident medical insurance")
add("XEXMIns[3]", "Urban resident medical insurance")
add("XEXMIns[4]", "New cooperative medical insurance:he-zuo-yi-liao")
add("XEXMIns[5]", "Government medical insurance:gong-fei")
add("XEXMIns[6]", "Medical aid")
add("XEXMIns[7]", "Private medical insurance: purchased by work unit")
add("XEXMIns[8]", "Private medical insurance: purchased by individual")
add("XEXMIns[9]", "Urban non-employed persons's health insurance")
add("XEXMIns[10]", "Long term health insurance")
add("XEXMIns[12]", "No insurance")
if (selected("EXEA001", "11")){
    add("XEXMIns[11]", value("EXEA001_1"))
}
```

XEXHaveMIns Covered by any of health insurance at the time of his/her death

```
if (selected("EXEA001", "1") || selected("EXEA001", "2") || selected("EXEA001", "3") ||
↪   selected("EXEA001", "4") || selected("EXEA001", "5") || selected("EXEA001", "6") ||
↪   selected("EXEA001", "7") || selected("EXEA001", "8") || selected("EXEA001", "9") ||
↪   selected("EXEA001", "10")|| selected("EXEA001", "11")) {
    add("XEXHaveMIns", "1")
}
```

```
if (selected("EXEA001", "12")) {
    add("XEXHaveMIns", "0")
}
```

XEXMInsPrivate Only covered by commercial insurance at the time of his/her death

```
if (!empty("EXEA001")){
add("XEXMInsPrivate", "0")
}
if ((!selected("EXEA001", "1")) && (!selected("EXEA001", "2")) && (!selected("EXEA001", "3")) &&
↪  (!selected("EXEA001", "4")) && (!selected("EXEA001", "5")) && (!selected("EXEA001", "6")) &&
↪  (!selected("EXEA001", "9")) && (!selected("EXEA001", "10")) && (!selected("EXEA001", "11")) &&
↪  (selected("EXEA001", "7") || selected("EXEA001", "8")))  {
    add("XEXMInsPrivate", "1")
}
```

XEXPMIns The types of health insurance before

```
add("XEXPMIns[1]", "Urban employee medical insurance :yi-bao")
add("XEXPMIns[2]", "Urban and rural resident medical insurance")
add("XEXPMIns[3]", "Urban resident medical insurance")
add("XEXPMIns[4]", "New cooperative medical insurance :he-zuo-yi-liao")
add("XEXPMIns[5]", "Government medical insurance :gong-fei")
add("XEXPMIns[6]", "Medical aid")
add("XEXPMIns[7]", "Private medical Insurance: Purchased by R's union")
add("XEXPMIns[8]", "Private medical Insurance: Purchased by Individual")
add("XEXPMIns[9]", "Urban non-employed persons's health insurance")
add("XEXPMIns[10]", "Long term health insurance")
add("XEXPMIns[12]", "No insurance")
if (selected("EXEB001", "11")){
    add("XEXPMIns[11]", value("EXEB001_1"))
}
```

XEXQuarantined Whether ever in quarantine

```
if (selected("EXV004", "1") || selected("EXV004", "2") || selected("EXV004", "3") || selected("EXV004",
↪  "4")|| selected("EXV004", "5")) {
    add("XEXQuarantined", "1")
} else {
    add("XEXQuarantined", "0")
}
```

XEXVAHospitalizationExcluded Wording: excluding days of hospital stay for COVID-19 treatment

```
if (greater("EXV003", "0")) {
    add("XEXVAHospitalizationExcluded", "Excluding days of hospital stay for COVID-19 treatment,")
}
```

XVCNotInQuarantine Wording: excluding days of quarantine during the pandemic

```
if (equal("XEXQuarantined", "1")) {
    add("XEXVCNotInQuarantine", ", excluding days of quarantine")
}
```

XKChildName Gen child list 2-26

```
for (var k = 1; k <= 25; k++) {
        add("XKChildName["+(k+1)+"]", value("ZChildName[k]"))
}
```

XKChildAndS Gen child's spouse list 27-51

```
for (var j = 1; j <= 25; j++) {
        add("XKChildAndS["+(j+26)+"]", value("XEChildAndS[j]"))
}
```

XEXGChildList1 Based on the answer of EXG003, generate first child name list

```
for (var i1 = 1; i1 <=25 ; i1++) {
    add("XEXGChildList1[i1]", value("ZChildName[i1]"))
}
```

```
for (var i1 = 26; i1 <=35 ; i1++) {
    add("XEXGChildList1[i1]", value("EXG003_1[i1]"))
}
```

XEXGChildList4 Based on the answer of EXG007, generate fourth child name list

```
for (var i1 = 1; i1 <=25 ; i1++) {
    add("XEXGChildList4[i1]", value("ZChildName[i1]"))
}
for (var i1 = 26; i1 <=35 ; i1++) {
    add("XEXGChildList4[i1]", value("EXG007_1[i1]"))
}
```

XEXGChildList2 Based on the answer of EXG022, generate second child name list

```
for (var i1 = 1; i1 <=25 ; i1++) {
    add("XEXGChildList2[i1]", value("ZChildName[i1]"))
}
for (var i1 = 26; i1 <=35 ; i1++) {
    add("XEXGChildList2[i1]", value("EXG022_1[i1]"))
}
```

XEXGChildList5 Based on the answer of EXG026, generate fifth child name list

```
for (var i1 = 1; i1 <=25 ; i1++) {
    add("XEXGChildList5[i1]", value("ZChildName[i1]"))
}
for (var i1 = 26; i1 <=35 ; i1++) {
    add("XEXGChildList5[i1]", value("EXG026_1[i1]"))
}
```

XEXGChildList3 Based on the answer of EXG032, generate third child name list

```
for (var i1 = 1; i1 <=25 ; i1++) {
    add("XEXGChildList3[i1]", value("ZChildName[i1]"))
}
for (var i1 = 26; i1 <=35 ; i1++) {
    add("XEXGChildList3[i1]", value("EXG032_1[i1]"))
}
```

XEXGChildList6 Based on the answer of EXG036, generate sixth child name list

```
for (var i1 = 1; i1 <=25 ; i1++) {
    add("XEXGChildList6[i1]", value("ZChildName[i1]"))
}
for (var i1 = 26; i1 <=35 ; i1++) {
    add("XEXGChildList6[i1]", value("EXG036_1[i1]"))
}
```

XEXGTotalValue Check the total value of legacy excluding house. If any item is missing or "-1", replace with "0".

```
add("XCK_EXF005", value("EXF005"))
if (empty("XCK_EXF005") || equal("XCK_EXF005", "-1")) {
  add("XCK_EXF005", "0")
}

add("XCK_EXF007", value("EXF007"))
if (empty("XCK_EXF007") || equal("XCK_EXF007", "-1")) {
  add("XCK_EXF007", "0")
}

add("XCK_EXF009", value("EXF009"))
if (empty("XCK_EXF009") || equal("XCK_EXF009", "-1")) {
  add("XCK_EXF009", "0")
}

add("XCK_EXF011", value("EXF011"))
if (empty("XCK_EXF011") || equal("XCK_EXF011", "-1")) {
  add("XCK_EXF011", "0")
}

add("XCK_EXF013", value("EXF013"))
```

```
if (empty("XCK_EXF013") || equal("XCK_EXF013", "-1")) {
  add("XCK_EXF013", "0")
}

add("XCK_EXEB006", value("EXEB006"))
if (empty("XCK_EXEB006") || equal("XCK_EXEB006", "-1")) {
  add("XCK_EXEB006", "0")
}

add("XCK_EXG010", value("EXG010"))
if (empty("XCK_EXG010") || equal("XCK_EXG010", "-1")) {
  add("XCK_EXG010", "0")
}

add("XCK_EXG012", value("EXG012"))
if (empty("XCK_EXG012") || equal("XCK_EG012", "-1")) {
  add("XCK_EXG012", "0")
}

add("XCK_EXG014", value("EXG014"))
if (empty("XCK_EXG014") || equal("XCK_EXG014", "-1")) {
  add("XCK_EXG014", "0")
}

add("XCK_EXG016", value("EXG016"))
if (empty("XCK_EXG016") || equal("XCK_EXG016", "-1")) {
  add("XCK_EXG016", "0")
}

add("XCK_EXG017", value("EXG017"))
if (empty("XCK_EXG017") || equal("XCK_EXG017", "-1")) {
  add("XCK_EXG017", "0")
}

add("XEXGTotalValue", value("XCK_EXF005")+value("XCK_EXF007")+value("XCK_EXF009")+value("XCK_EXF011")+value("XCK_EXF013")+value("XCK_EXEB006")+value("XCK_EXG010")+value("XCK_EXG012")+value("XCK_EXG014")+value("XCK_EXG016")+value("XCK_EXG017"))
```

ZChildName Name of respondent’s child

Appendix Function Descriptions

A. Functions in the Questions

Hard Check The hard check function `hc(a,b,c)` is used to avoid logically impossible records for numerical variables. If the record for the corresponding question does not meet the conditions specified in hc, the interviewer is required to return and modify the answer.
Parameters:

- a: Range of values
- b: *int* indicates that only integers are allowed; *real* indicates that decimals are allowed
- c: Symbol used when the respondent refuses to answer or does not know the answer to the corresponding question; $\varnothing$ indicates that such records are not allowed

Soft Check The soft check function `sc(a,b,c)` is used to alert for outliers in numerical variables. If the record for the corresponding question does not meet the conditions specified in sc, the interviewer is required to confirm again.
The parameters of `sc(a,b,c)` are identical to those of the hard check function.

Unfolding Brackets The unfolding brackets function `ub(a,b)` is used to obtain range information for a specific amount (e.g., income) when respondents refuse to answer or are not very clear about it.
Parameters:

- a: Array representing the triggering conditions for unfolding brackets. If the record for the corresponding question is within this array, the unfolding brackets are triggered.
- b: Array representing the breakpoints for unfolding brackets.

In questions involving unfolding brackets, the code for refusal to answer or not knowing is marked as "-1."

Conflict The conflict check function `conflict(a,b,…,c)` is used to examine whether the input answers contain logical errors. If the last option, c, is selected simultaneously with any of the previous options, an error message is triggered. Interviewers must reconfirm and update the answers.

Transfer Picture The picture transfer function `transferPic()` is used to display images.

B. Functions in Auxiliary Variables

Auxiliary variables in the questionnaire are calculated from original variables based on predefined rules. The construction of these auxiliary variables involves some basic control structures, such as `if-else-if` and `for` loops, and also includes some custom functions. The current questionnaire involves the following custom functions:

- `add(x,y)`: Sets the value of variable x to y.
- `value(x)`: Returns the value of variable x.
- `selected(x,y)`: Determines if y is in the array x, typically used to check if an option is se-

lected in a corresponding multiple-choice question. Returns `true` if selected, otherwise `false`.

- `count(x)`: Returns the size of array `x` (i.e., the number of elements), typically used to determine the number of selected options in a multiple-choice question.
- `range(x,min,max,decimal,special)`: Checks if variable `x` falls within the specified range. `min` indicates the lower bound of the range, `max` indicates the upper bound, `decimal` indicates whether decimals are allowed (`true` allows decimals, `false` does not), and `special` indicates values that are specially allowed outside the range (missing indicates none).
- `greater(x,y,equal)`: Checks if variable `x` is greater than or equal to `y`. `equal` controls whether the comparison is greater than or greater than or equal to (`true` indicates greater than or equal to, missing indicates greater than).
- `equal(x,y)`: Checks if variable `x` is equal to `y`. Returns `true` if equal.
- `empty(x)`: Checks if variable `x` is empty or nonexistent. Returns `true` if empty or nonexistent.
